# *o*‐Terphenyl‐Based Family of Conjugated Macrocycles: Selective Recognition of Phenylalanine in Water and Interaction With Insulin

**DOI:** 10.1002/anie.202525972

**Published:** 2026-03-18

**Authors:** Swapnil Ghule, Sayan Sarkar, Maria Eugenia Pérez‐Ojeda Rodriguez, Mark Spector, Frank Hampel, Evgeny A. Kataev

**Affiliations:** ^1^ Department of Chemistry and Pharmacy Friedrich Alexander University of Erlangen‐Nürnberg Erlangen Germany

**Keywords:** amino acid, macrocycles, molecular recognition, pi‐systems, terphenyl

## Abstract

The design of supramolecular receptors for amino acids presents a fundamentally challenging yet highly promising avenue of research. Phenylalanine attracts special attention because of its multifaceted role in living organisms. Although many synthetic hosts have been explored for recognition of phenylalanine (Phe), besides cucurbiturils, there are no receptors that show selectivity for Phe over other aromatic amino acids and related aromatic neurotransmitters in water. Toward addressing this challenge, we explored pi‐conjugated water‐soluble macrocycles with hydrophobic pockets. A new family of *o*‐terphenyl‐based macrocycles, TP[n] (*n* = 2‐8) was synthesized using the Yamamoto reaction. Macrocycles up to the octamer, TP[2]‐TP[8], were isolated and fully characterized. X‐ray studies reveal that these macrocycles can form folded conformations stabilized by stacking interactions. TP[3] was identified as the most selective host for Phe with an affinity of 7 x 10^3^ M^−1^ in water. The hosts, composed of 2–5 subunits were found to effectively inhibit protein aggregation according to the fluorescence assay using thioflavin T. The discovery of the new family of macrocycles paves the way for designing hosts with diverse architectures, precisely tailored geometries, and optimized binding properties capable of targeting not only individual amino acids but also entire protein surfaces.

## Graphical abstract

This study presents the synthesis and binding properties of a new family of *o*‐terphenyl‐based macrocycles, TP[n] (*n* = 2‐8). The trimer variant efficiently binds phenylalanine in water with a 10‐fold selectivity compared to other amino acids and aromatic neurotransmitters. This distinctive trait allows its application in inhibiting protein aggregation. The larger macrocycles exhibit similar properties, likely due to their ability to recognize protein surfaces.

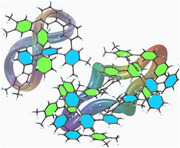

## 1 Introduction

Engineering synthetic receptors to recognize and bind specific amino acids with high selectivity in water or biological fluids is a highly appealing research area [1, 2, 3, 4, 5, 6, 7]. Such receptors can mimic natural molecular recognition by enzymes and antibodies. Achieving considerable binding selectivity is highly challenging, yet it is crucial for applications like sensing and diagnostics [8, 9, 10], targeted drug delivery, and understanding protein interactions [9, 11] for the purification and functionalization of amino acids.

Phenylalanine is a particularly interesting target for supramolecular chemistry because it is an essential amino acid with a hydrophobic residue that helps to stabilize protein structures via dispersion interactions. It is a key precursor for tyrosine and neurotransmitters, as well as hormones such as dopamine, norepinephrine, and adrenaline. Studies have shown that at high (pathological) concentrations, phenylalanine aggregates into ordered fibrillar structures that closely resemble classic amyloid seen in Alzheimer's disease [12]. Targeting phenylalanine in protein structure is a significant challenge. Artificial hosts designed for this purpose can work as inhibitors, influence protein aggregation, and modulate protein‐protein interactions [13, 14]. Selective binding of an amino acid residue requires an appropriately sized inner cavity in the supramolecular host and specific binding sites for distinguishing it from other residues [15, 16]. The recognition of a larger part of the protein arranged in a specific three‐dimensional structure is even more challenging because of the lack of any major binding pocket or well‐defined binding sites. Therefore, the design of such systems involves many parameters, such as the shape of the sequence, type of amino acids involved and etc.

The obvious solution to the problem is to construct receptors with a preorganized hydrophobic cavity. Recently, several rigid aromatic systems bearing a hydrophobic cavity and functionalized with negatively or positively charged groups have been reported. For instance, charged naphthalene‐based macrocycles [17], naphthotubes [18, 19, 20, 21, 22], methylene‐bridged macrocycles [23], metallocages [24, 25, 26, 27], conjugated macrocycles with deep hydrophobic cavities that show strong affinity for steroids [28], have been recently designed [22, 29]. Calixarene derivatives with attached charged groups have been identified as promising hosts for the recognition of aromatic residues. They were utilized to deliver drugs [30] and inhibit amyloid fibrillation [31, 32]. Some of the newly developed macrocycles can surpass the affinities of cucurbiturils and cyclodextrins for aromatic guests. These achievements have been possible thanks to the rigid hydrophobic pocket and attached solubilizing groups serving as additional binding sites for supramolecular recognition [20, 33, 34, 35, 36]. Water solubility, a hydrophobic cavity, and a rigid structure are the features that are essential for an artificial host intended to interact with biologically important molecules [21, 37, 38]. Many of these new systems operate in water and exhibit high‐affinity binding, but none demonstrate selective binding of phenylalanine [39]. It appears to be challenging to achieve selectivity for phenylalanine over other amino acids.

Knowing that C─H⋯π interactions significantly contribute to the overall stability of the protein [40] and that the most prominent interactions occur between CH‐donors and aromatic π‐acceptors, we turned to investigate the receptors based on conjugated aromatic rings [41]. The size of such receptors can be varied to optimize the conformation and binding sites. To address this challenge, we proposed generating a family of macrocycles of different sizes starting from one building block via the Yamamoto homocoupling reaction. The rigid structure and non‐planarity of the macrocycles can be beneficial for optimizing affinity for a specific residue or a particular surface of the protein. The structure of such macrocycles can resemble peptide loops and can potentially demonstrate antibody‐like affinity.

In this work, we introduce a new family of *o*‐terphenyl macrocyclic receptors, TP[n]COOH, which contain carboxylic groups, and explore their binding properties with amino acids and proteins. We demonstrate that π‐conjugated macrocycles can recognize aromatic amino acids and neurotransmitters in aqueous solutions. The macrocycle with three subunits, TP[3]COOH, was found to bind phenylalanine with an affinity of 7 x 10^3^ M^−1^ (pH 7.4, buffer), which is about 10 times stronger than binding constants for other structurally similar guests, such as tryptophan, tyrosine, and neurotransmitters. Experimental and theoretical studies show that a new family of receptors, starting from the trimer, is relatively flexible and can adopt cavitand‐like or folded helical conformations. They can effectively bind and light up thioflavin T (ThT) by restricting its conformation, similar to how this dye detects proteins or DNA. Using the ThT assay, we found out that TP[2]COOH‐TP[5]COOH can interact with phenylalanine residues in peptide sequences, thereby inhibiting protein aggregation. Induced CD bands in circular dichroism and ITC (isothermal titration calorimetry) measurements confirm a strong interaction between insulin and macrocycles TP[3]COOH and TP[5]COOH.

## 2 Results and Discussion

### 2.1 Synthesis of Macrocycles

**FIGURE 1:**
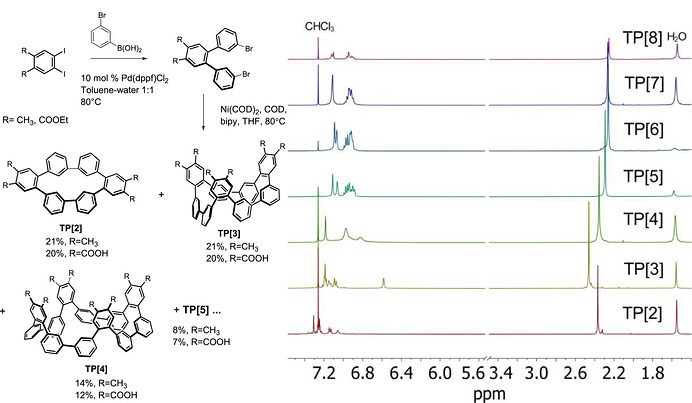
Synthesis of a family of macrocycles TP[2]‐TP[8]. Dppf ‒ 1,1′‐bis(diphenylphosphino)ferrocen, bipy ‒ 2,2′‐bipyridin. Stacked ^1^H NMR (CDCl_3_) spectra of isolated macrocycles bearing R = CH_3_.

We selected *o*‐diphenylbenzene (*o*‐terphenyl) [42] as a simple starting building block to synthesize a family of macrocycles via coupling reactions. We introduced methyl and ester groups into the structures. The ester groups can be converted to carboxylic acid groups in the final step, thereby imparting water solubility and multiple negative charges to the macrocyclic receptors. Previous reports indicated that terphenyl can undergo oligomerization in the Scholl reaction [43]. However, planar oligomeric products are formed without any structured cavity or controlled connectivity. Oligo‐ and polyphenylenes [44, 45] can also be synthesized using various catalyst–oxidant combinations [46, 47] or via the Yamamoto reaction [48, 49, 50, 51, 52]. Additionally, the platinacycle method has been shown to be very effective, beginning with metallocycle intermediates that undergo reductive elimination to yield a macrocyclic structure [48, 53, 54]. In this work, we utilized the Yamamoto reaction to synthesize a family of macrocycles TP[2]‐TP[8] that bear substituents on the central benzene ring (Figure 1). By maintaining the starting material (dibromide) concentration below 2 mM, TP[2] is produced as the main product with a 30% yield. Higher oligomers are generated in yields of less than 1%. When the concentration is increased to about 10 mM, additional oligomeric macrocycles, reaching up TP[8], can be detected by mass spectrometry and isolated. The optimum concentration for the synthesis of a trimer macrocycle TP[3] is 80 mM, at which the macrocycle can be isolated with 21% yield. Other macrocycles are also formed with increased yields ranging from 14% for TP[4] to 0.5% for TP[8]. Using higher reagent concentrations led to a significant decrease in yields and the formation of undesired polymeric products.

The structure of TP[2] with R = H has been previously reported and was prepared using Grignard reagents and copper(II) chloride [47]. While TP[2] is rigid, larger macrocycles can display greater flexibility due to free rotation along the C─C bond. According to the NMR data, TP[2]CH_3_ and TP[3]CH_3_ contain magnetically equivalent terphenyl fragments, which is consistent with free rotation along this bond. In contrast, ^1^H NMR of TP[4]COOEt displays two sets of signals corresponding to two pairs of nonequivalent terphenyls. It is likely that the rotational freedom in TP[4]COOEt is limited, and several conformers exist [55]. Dynamic ^1^H NMR studies (Figure S27) of TP[4]COOEt show that at elevated temperatures, the signals begin to coalesce, indicating the overcoming of the rotational barrier. For higher‐order oligomers, starting from TP[5], the signals are significantly broadened, indicating a dynamic behavior of the structures at the NMR timescale (Figure 1). It should be noted that aromatic protons shift upfield as the ring size of TP[n] increases.

**FIGURE 2:**
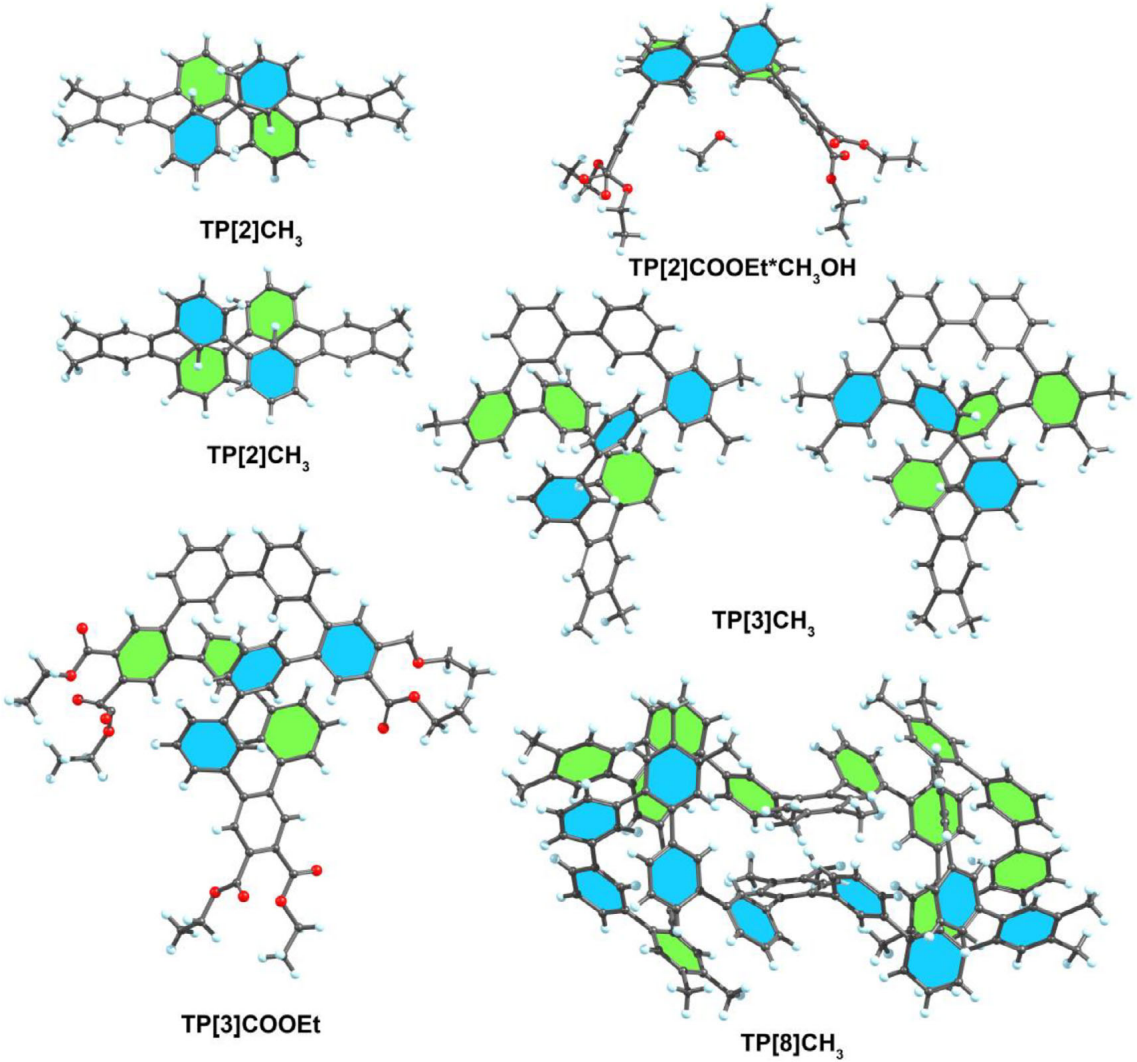
X‐ray crystal structures of macrocycles. Blue‐painted rings are located above the green‐painted.

The variety of conformations was indeed observed in the molecular structures of free receptors obtained by a single crystal X‐ray analysis (Figure 2). Two distinct conformations, helical and cavitand‐like, were observed for TP[2]CH_3_ from crystallizations conducted in different solvents. Two enantiomers were identified in the crystal of TP[2]CH_3_ formed from a methanol‐toluene mixture. Crystallization of TP[2]COOEt from methanol resulted in a cavitand‐like conformation with methanol coordinated via four C─H⋯O hydrogen bonds in the center of the cavity.

The difference in conformations of TP[2]CH_3_ and TP[2]COOEt suggests that benzene rings can rotate freely, with the resulting conformation in the solid state determined by intermolecular and solvent interactions. Crystallographic data for TP[3] were obtained for both derivatives with R = CH_3_ and COOEt. Both structures display a similar figure‐eight shape, which also exists as two enantiomers in the solid state. Mixing TP[3]COOH with chiral (S)‐(−)‐α‐methylbenzylamine in methanol did not lead to diastereoisomer formation. Therefore, TP[3]COOH has enough conformational flexibility to allow rapid interconversion of enantiomers even at room temperature. A quite unusual structure was observed for TP[8]CH_3_, stabilized within the crystal lattice by multiple intramolecular and intermolecular π–π interactions. These interactions create a densely packed double‐stranded structure resembling the quaternary structure of proteins. Such multiple interactions may also occur in solution and could explain the broad signals seen in the ^1^H NMR spectrum (Figure 1).

### 2.2 Binding Studies With Amino Acids

The presence of a hydrophobic pocket is crucial for binding aromatic amino acids, while carboxylate groups can provide electrostatic interactions and hydrogen bonds. Based on these molecular recognition principles, we focused on acid‐functionalized macrocycles that are soluble in aqueous solutions. Although the macrocycles are fluorescent with an emission at 430 nm, the excitation range overlaps with the absorption of aromatic acids, complicating the assessment of binding data by fluorescence spectroscopy. Therefore, binding constants were determined through ^1^H NMR titration in a buffered solution at pH 7.4. According to the pH‐dependent fluorescence studies, the p*K_a_* values of the carboxylate groups are around 5, ensuring full deprotonation at neutral pH in water.

**FIGURE 3:**
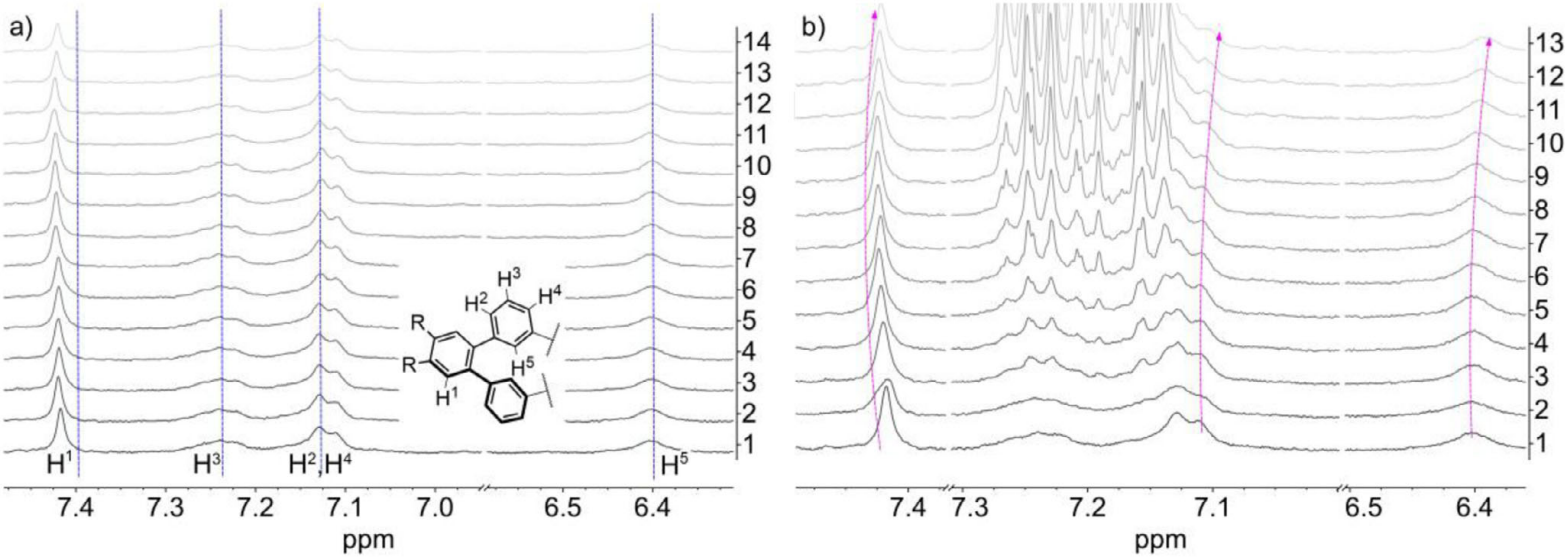
^1^H NMR titration of TP[3]COOH with (a) alanine and (b) phenylalanine in 50 mM MOPSO buffer (pH 7.4). The lines and arrows highlight chemical shifts as the concentration of amino acids increases.

To understand the binding properties of macrocycles, we selected a series of aromatic amino acids: alanine as a non‐aromatic amino acid, and neurotransmitters with one or two positive charges at neutral pH. The results of the binding studies are shown in Table 1. Due to broad signals in ^1^H NMR for larger macrocycles, we only performed titrations with TP[2]COOH, TP[3]COOH, and TP[4]COOH functionalized with carboxylate groups to ensure a negative charge and good solubility in water. The macrocycles vary in the number of negatively charged carboxylate groups and geometry, which likely influence their selectivity for aromatic amino acids. As seen from the titration results, TP[2]COOH has only moderate affinity for dopamine, arginine, and lysine, and no detectable affinity for amino acids. The addition of excess amino acids resulted in no observable proton shifts. In contrast to the dimer, TP[3]COOH exhibited the strongest affinity for phenylalanine. Overall, TP[3]COOH demonstrates significant selectivity over other aromatic amino acids, lysine, arginine, and neurotransmitters. For example, for titration with alanine, the shifts of aromatic protons are small (<0.05 ppm). In several ^1^H NMR titrations with amino acids, an inflection point in the binding isotherm suggests a stepwise 1:1 and 1:2 binding stoichiometry (Figure 3b). However, the fitting analysis shows that the binding of a second amino acid is much weaker. A similar result was obtained from ITC data for Phe and TP[3]COOH, which showed *K_11_* = 15000 M^−1^ and *K_12_* = 680 M^−1^. We also tested dipeptide L‐γ‐glutamyl‐L‐phenylalanine that models phenylalanine in the peptide chain. According to the ^1^H NMR data, the determined affinity constant exceeds 10^4^ M^−1^, suggesting a high degree of complementarity of the dipeptide and the host (cf. SI). While the trimer host binds amino acids, TP[4]COOH showed selectivity for dopamine but no detectable affinity for amino acids. This is likely due to its higher conformational flexibility and the lack of a preorganized cavity.

**TABLE 1:** Binding constants *K_11_* (M^−1^) and *K_12_* (if present) obtained from ^1^H NMR titrations of macrocycles with amines and amino acids. Conditions: 50 mM MOPSO buffer in D_2_O, pH 7.4, 20% DMSO‐*d_6_*.

Guest	TP[2]COOH	TP[3]COOH	TP[4]COOH
Histamine	^a^	122 ± 3	45 ± 5
Serotonin	^a^	154 ± 2	46 ± 5
Dopamine	157 ± 5	116 ± 5	496 ± 65; 232 ± 18
L‐arginine	79 ± 5	136 ± 6	^b^
L‐lysine	95 ± 6	100 ± 2	16 ± 3
L‐Dopa	^b^	134 ± 22	^b^
L‐Histidine	^b^	388 ± 26; 492 ± 11	4 ± 0.5
L‐Tyrosine	^b^	1015 ± 41, <1	^b^
L‐Alanine	^b^	182 ± 24; 111 ± 14	^b^
L‐Phenylalanine	^b^	7107 ± 128; <1	^b^
L‐Tryptophan	^b^	722 ± 25; 349 ± 10	72 ± 12

^a^ Signals overlap with the signals of the receptor.

^b^ No proton shifts were detected.

**FIGURE 4:**
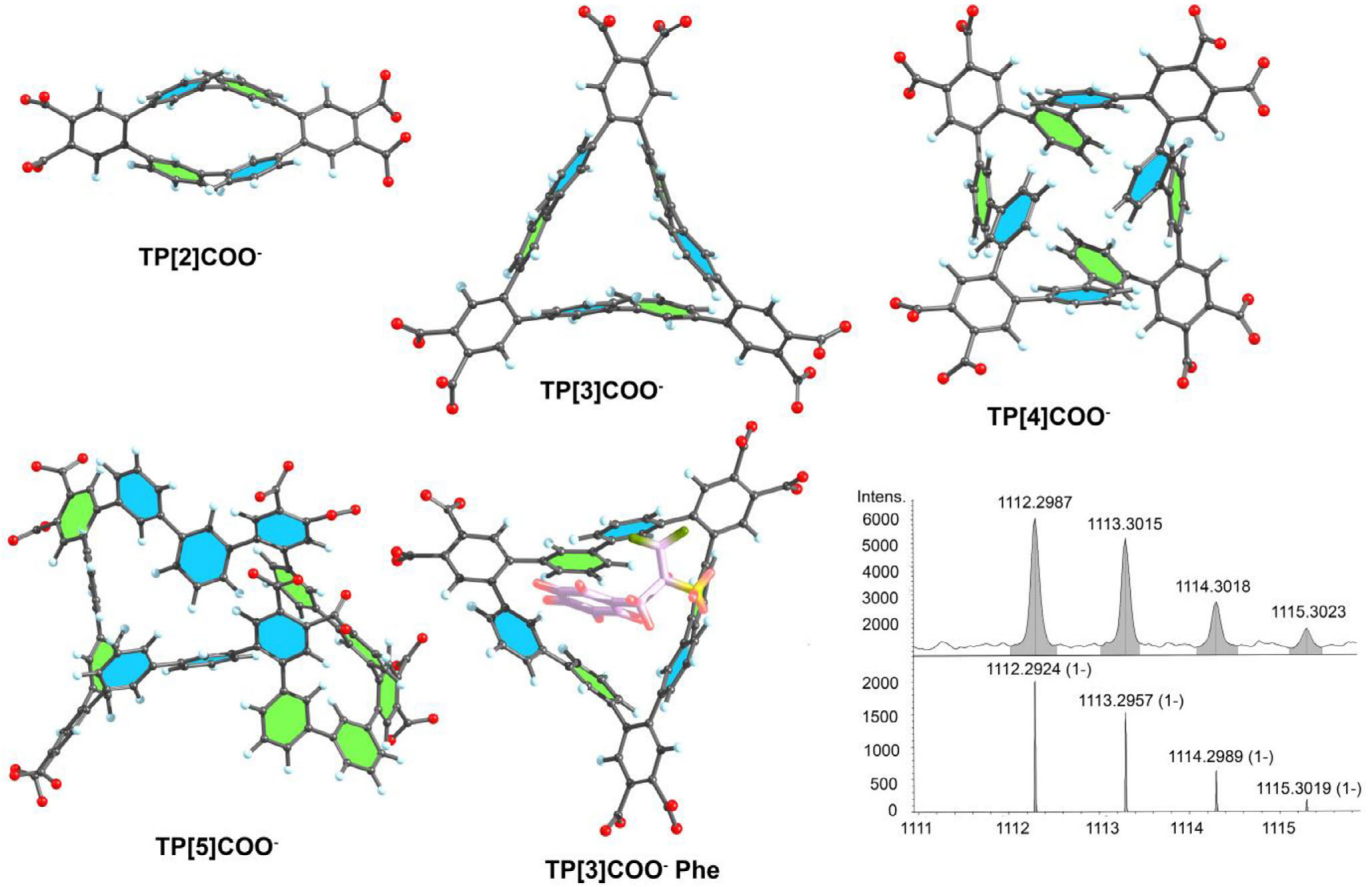
Structure of TP[2]–TP[5] according to conformational analysis and DFT calculations. Optimized structure of the phenylalanine‐TP[3]COOH complex TP[3]COO‐Phe, where phenylalanine is in a zwitterion form. Comparison of the observed and calculated *m/z* signal in ESI‐MS (‐), which corresponds to the negatively charged complex [TP[3]COOH∙Phe─H^+^]^−^.

The X‐ray structures shown in Figure 2 reveal that TP[3]COOH has a hydrophobic cavity provided by aromatic rings and sufficient flexibility to undergo conformational rearrangement. To find the most favorable conformation of TP[3]COOH in water, we have conducted a global optimization algorithm (GOAT) [56] by using the software package Orca 6.2.2 [57, 58] followed by the r2SCAN‐3c [59] geometry optimization utilizing the SMD solvation module for water [60]. The most energetically favorable structure resembled a triangle forming a hydrophobic cavity inside the macrocycle with negatively charged carboxylate groups located on the periphery. The optimized structure with phenylalanine (Figure 4b) indicates a perfect cavity for the benzene ring of the guest. This benzene ring forms π–π interactions with one of the biphenyl fragments of the macrocycles, with the shortest distance 3.4 Å, and two CH–π interactions with the other two such side‐walls 2.4 and 2.5 Å. The results of the calculations confirm that TP[3]COO^−^ can adopt a conformation different from that observed in X‐ray data. The hydrophobic cavity is perfectly suited for the encapsulation of a single benzene ring. We thus suggested that a similar binding mode could occur during the recognition of polypeptide sequences containing phenylalanine residues. 2D ROESY experiments confirmed the absence of a folded conformation in solution. ESI (‐) mass spectrometry of a 1:1 mixture of TP[3]COOH and phenylalanine produced an intense signal corresponding to the host‐guest complex [TP[3]COOH∙Phe‐H^+^]^−^ (Figure 4). A similar conformational analysis was performed for dimer, tetramer, and pentamer macrocycles. The dimer results show a structure similar to that seen in X‐ray analysis. TP[4]COO^−^ exhibits an interesting bowl‐like structure with benzene rings packed inside through π–π and H–π interactions, with the carboxylate groups pointing outward. Due to intramolecular interactions between the benzene rings, the structure lacks a cavity. According to the calculations, TP[5]COO^−^ does not have a symmetrical structure like other macrocycles and likely can adopt many possible conformations in solution.

### 2.3 Inhibition of Protein Aggregation

Selective recognition of phenylalanine is a key property of supramolecular hosts that enable interaction with aromatic residues in proteins and has been effectively used to inhibit protein aggregation [61]. This process is associated with amyloidosis, such as Parkinson's and Alzheimer's diseases. Cucurbiturils [62] are one of the most efficient binders of aromatic guests, thanks to their appropriately‐sized hydrophobic cavity. For instance, CB[7] can bind phenylalanine residues in β‐amyloids [63] and in insulin [64] with *K_a_* up to 10^7^ M^−1^ in an aqueous solution. These residues are responsible for initiating the aggregation of proteins via hydrophobic clustering, and hence, their encapsulation has proven to be an efficient strategy to inhibit the aggregation. Macrocycles TP[n] (R = COOH) possess a different structure and may enable different modes of interaction with the protein surface. Unlike cucurbituriles, TP[n] consist solely of benzene rings, which could enhance π–π interactions with aromatic guests. They carry multiple negative charges, preventing host–guest complexes from aggregating. The structures exhibit a certain degree of flexibility, allowing them to adapt to the geometry of the protein surface.

To study protein aggregation in the presence of TP[n]COOH, we investigated insulin fibrillation by changing the insulin:TP[n]COOH ratio. In these and the following experiments, we used TP[n]COOH with *n* = 2–5, which can be synthesized in sufficient amounts. According to our screening experiments, a 1:1 ratio was sufficient to achieve remarkable inhibition of aggregation at 60°C. The fluorescence assay for aggregation detection utilizes thioflavin T (ThT). Upon binding of ThT to cross‐β structures, the rotation of the C─C bond linking the benzothiazole and aniline rings is restricted, which in turn reduces non‐radiative energy loss, and fluorescence enhancement is observed. Negatively charged molecules are also known to inhibit amyloid formation [65, 66, 67]. Therefore, we initially tested phthalic acid as a control compound, which had no effect on insulin aggregation.

**FIGURE 5:**
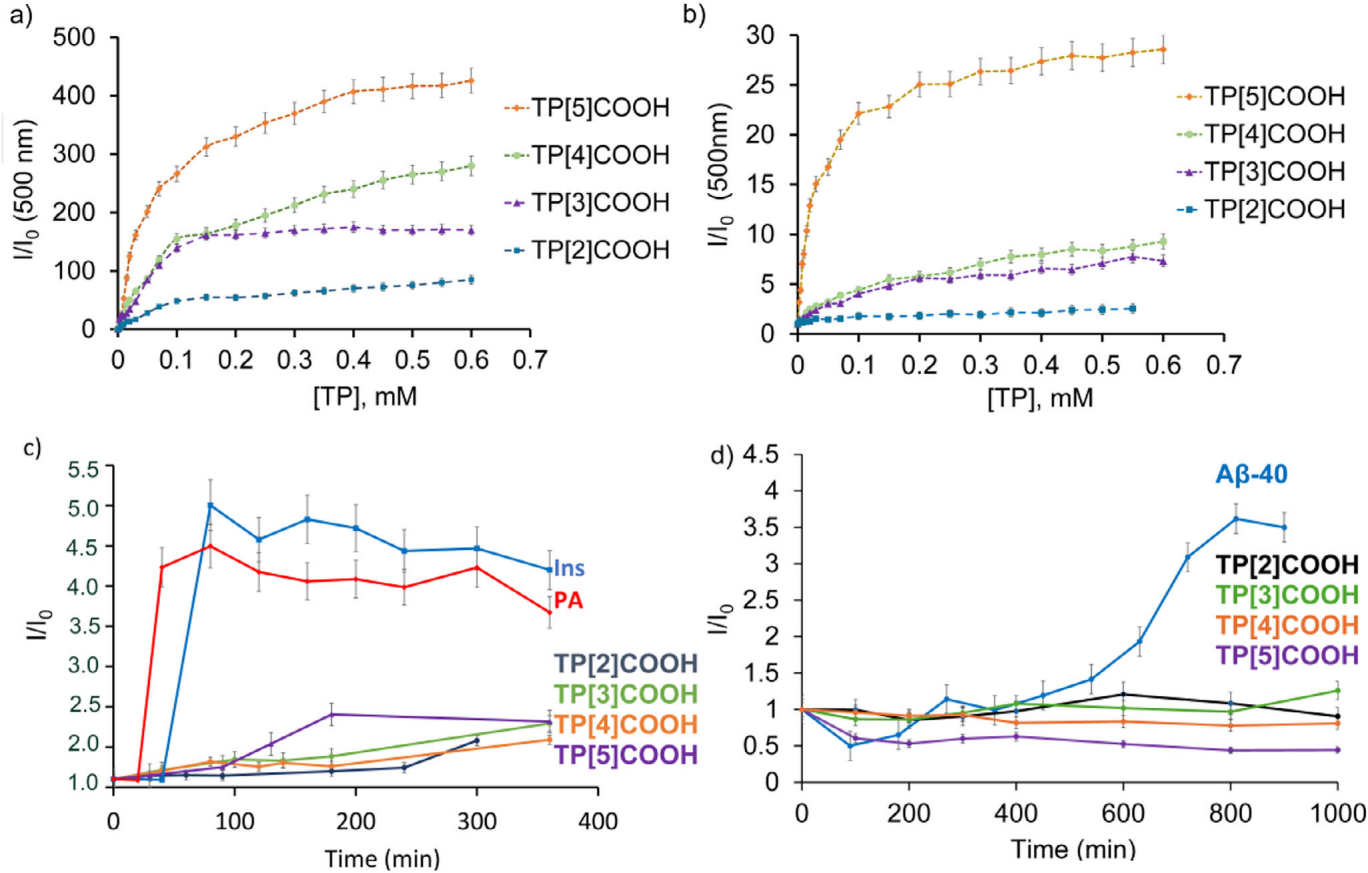
Fluorescence titration of ThT (10 µM) with macrocycles in (a) 25 mM HCl, 0.1 M NaCl, pH 2.0 (5% DMSO, ex. 415 nm); (b) in a 20 mM phosphate buffer, pH 7.4 (5% DMSO, ex. 415 nm). (c) Insulin (50 µM) aggregation kinetics as determined by fluorescence assay with ThT (2 mM) at 60°C in the absence of additives (Ins, 50 µM) and in presence of phthalic acid (PA) or macrocycles TP[2]COOH‐TP[5]COOH (50 µM) at pH 2.0 (20 mM HCl, 0.1 M NaCl, 5% DMSO, 60°C). (d) Aggregation of Aβ40 (5 µM) in the presence of macrocycles at 37°C (10 equiv.) and ThT (0.1 mM) in a 20 mM phosphate buffer (pH 7.4, 30 mM NaCl).

Next, we examined our family of macrocycles to determine whether they can bind ThT in a buffered solution at two different pH levels (pH 2.0 and pH 7.4), where insulin and Aβ40 amyloid aggregation were studied, respectively. Adding macrocycles to the ThT solution resulted in a strong fluorescence enhancement, indicating stabilization of ThT's conformation within the host‐guest complex. Notably, a 400‐fold enhancement after saturation was seen with TP[5]COOH at pH 2. The host‐guest complex with the dye is likely to be favorable under these conditions (Figure 5a,b). The determined binding constants of ThT with macrocycles at pH 2 were in the range 10^3^–10^4^ M^−1^: TP[2]COOH 6600 M^−1^, TP[3]COOH 16,980 M^−1^, TP[4]COOH 5120 M^−1^ and TP[5]COOH 12,000 M^−1^. The overall fluorescence enhancement at pH 7.4 was approximately 30‐times lower. However, the binding was more selective for TP[5]COOH, with values: TP[2]COOH 790 M^−1^, TP[3]COOH 4360 M^−1^, TP[4]COOH 2880 M^−1^ and TP[5]COOH 28840 M^−1^. These binding studies confirm that macrocycles can bind ThT similarly to aggregated proteins or nucleic acids, leading to strong fluorescence enhancement. Given that ThT binds to both TP[n] and aggregated insulin, thereby enhancing fluorescence, we conducted several control experiments to prove the inhibition efficiency of macrocycles. The kinetic studies involved mixing insulin and macrocycle in equal amounts, with ThT concentrations being 10 times lower or higher than that of the macrocycle. In another control experiment, insulin and macrocycles were combined and incubated for 5 h before the addition of excess ThT and fluorescence measurements. In all these experiments, significant inhibition of aggregation was observed when comparing ThT fluorescence intensity added to free insulin and mixtures with macrocycles. Additionally, ThT was mixed with aggregated insulin, followed by the addition of macrocycles TP[2]COOH‐TP[5]COOH. The macrocycle displaced ThT from the complex, causing fluorescence quenching (Figure S48). This indicates that the observed fluorescence enhancement results from insulin aggregation rather than the interaction of macrocycles with ThT.

First, we studied insulin aggregation at pH 2. As shown in Figure 5c, insulin begins to aggregate after 1 h of incubation. While phthalic acid had no effect, all macrocycles were effective in inhibiting insulin fibrillation. The inhibition mechanism is proposed to be similar to that of CB[7], where binding occurs at the terminal phenylalanine residue in insulin [63]. Although larger macrocycles did not show detectable affinity for phenylalanine, they may bind to insulin via a different mechanism involving protein surface recognition. We also tested sequence Aβ40, a 40‐amino acid peptide derived from amyloid precursor protein, which is more abundant in cerebrospinal fluid. The fibrillation experiment conducted in a phosphate buffer at pH 7.4 and 37°C also demonstrated the effectiveness of our macrocycles in inhibiting protein aggregation. Under the selected conditions, the fluorescence of ThT and Aβ40 sample reaches a plateau after 14 h (Figure 5d). When Aβ40 and macrocycles were mixed in a 1:10 ratio, suppression of amyloid fibrillation was observed. Combining the results of experiments with insulin and Aβ40, we can conclude that all macrocycles efficiently inhibit aggregation and suggest potential use for therapeutic purposes.

**FIGURE 6:**
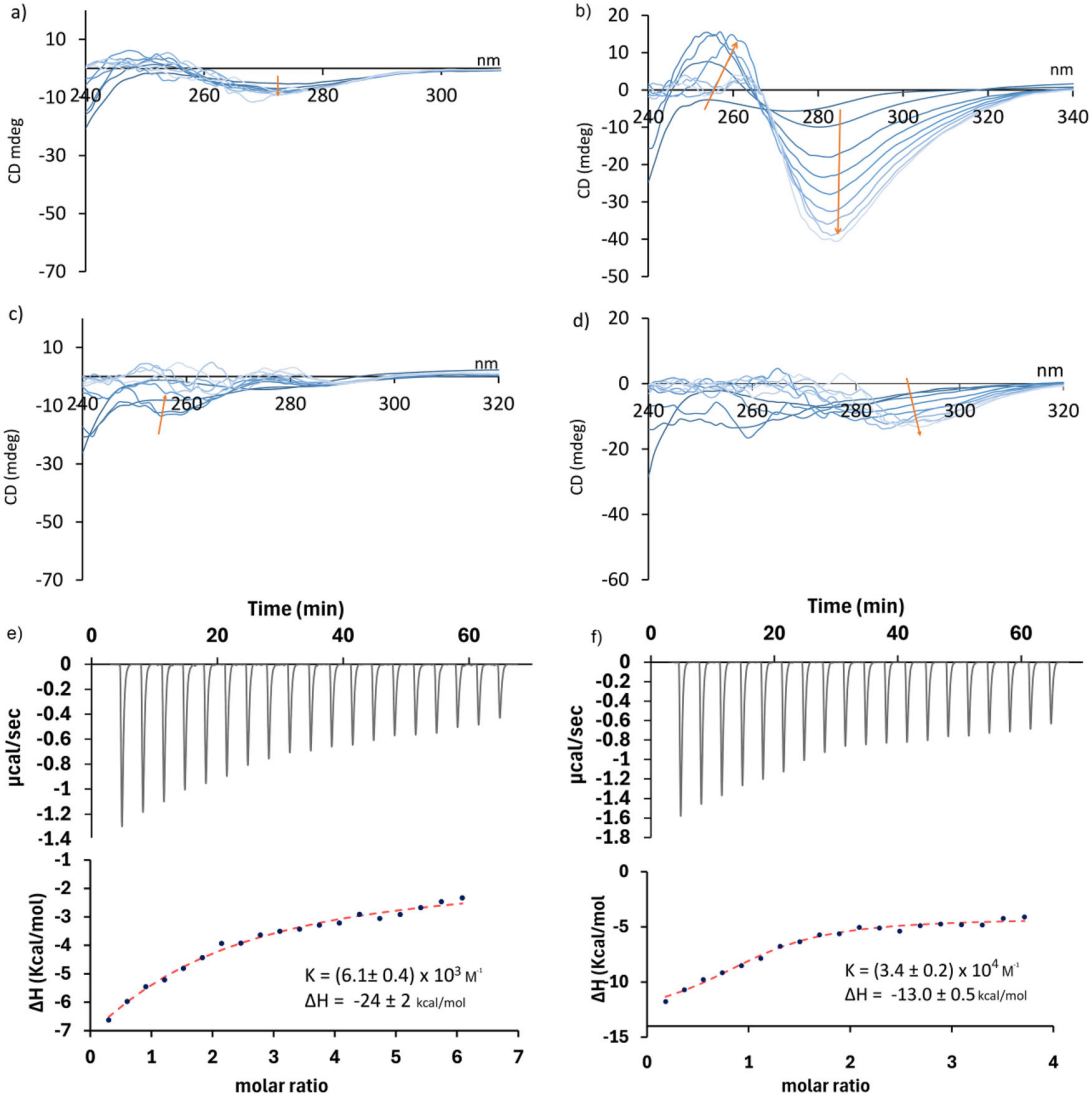
CD spectra of insulin (0.05 mM) in the presence of increasing equivalents of (a) TP[2]COOH, (b) TP[3]COOH, (c) TP[4]COOH, and (d) TP[5]COOH. ITC titration profiles for (e) TP[3]COOH and (f) TP[5]COOH at pH 7.4 in a phosphate buffer.

Since macrocycles TP[2]COOH‐TP[5]COOH can interact with insulin and inhibit aggregation, we aimed to study these interactions using CD spectroscopy. When macrocycles specifically interact with insulin, strong changes in CD spectra were expected. The macrocycles were added to the insulin solution in increments up to 2.5 equiv., and the spectra were recorded. Titration with TP[2]COOH and TP[4]COOH caused a decrease in CD band intensities, while adding TP[3]COOH and TP[5]COOH resulted in the appearance of quite intense induced CD bands (Figure 6b,d). The most significant effect was observed with TP[3]COOH─ the receptor with the highest affinity for phenylalanine. This fact suggests that TP[3]COOH likely interacts with phenylalanine residues, inducing electronic changes in the macrocycle due to the chiral environment. In contrast, TP[2]COOH and TP[4]COOH interact non‐specifically, and no new ICD bands were observed. Notably, the CD spectrum of TP[4] lacks sharp features indicative of the loss of ordered secondary structures. According to the isothermal titration calorimetry (ITC, Figure 6), all macrocycles were bound to insulin with affinities ranging from 10^3^ to 10^4^ M^−^^1^: TP[2]COOH, *K_a_* < 10^3^; TP[3]COOH, *K_a_* = (6.1 ± 0.4) × 10^3^ M^−^^1^; TP[4]COOH, *K_a_* = (1.3 ± 0.3) × 10^3^ M^−^^1^; TP[5]COOH, *K_a_* = (3.4 ± 0.2) × 10^4^ M^−^^1^. Interestingly, the binding constant for TP[5]COOH has the highest value, likely due to the greater number of interaction sites. These results suggest that larger macrocyclic receptors can interact with a larger surface of the protein and thus show enhanced affinity.

## 3 Conclusion

In summary, we have developed a new family of *o*‐terphenyl‐based conjugated macrocycles TP[2]‐TP[8] that can be prepared from simple building blocks *via* Yamamoto coupling. The macrocycles functionalized with carboxylic acid groups are water‐soluble hosts with inner cavities that vary in size and shape. X‐ray crystal structure analysis has revealed various conformations of the macrocycles resulting from rotation along the C─C bond. Experimental and theoretical modeling data show that TP[3]COOH is the only member with an appropriate cavity to bind phenylalanine with the highest affinity. NMR measurements demonstrated the ability of TP[3] to selectively recognize phenylalanine in a buffered solution (pH 7.4) with an affinity of 7 x 10^3^ M^−1^, as compared to other amino acids and neurotransmitters. DFT modeling of the host–guest complex confirmed the coordination of the benzene ring within the macrocycles' cavity via π–π and H–π interactions. The capability to bind phenylalanine was further tested in amyloid aggregation experiments involving TP[2]COOH‐TP[5]COOH using the ThT assay. All macrocycles have been shown to inhibit the aggregation of insulin (pH 2.0) and amyloid Aβ40 (pH 7.4). This behavior suggests that our hosts can interact with peptides containing multiple phenylalanine residues. A strong induced CD band has been observed in the presence of TP[3]COOH. The interaction between insulin and o‐TP[n] has been further confirmed by ITC measurements. The new family of *o*‐TP[n] hosts provides clear evidence that π‐conjugated systems can be optimized to function in water and bind aromatic amino acids. This property is key to tackling the challenges in inhibiting protein aggregation. The most promising TP[3] member offers numerous opportunities for further functionalization, optimization of its binding properties, cavity design, and applications in biology. Larger macrocycles display interesting folded conformations and are highly promising as hosts for protein surface recognition, potentially affecting protein function through folding and unfolding strategies. This research is currently ongoing.

## Conflicts of Interest

The authors declare no conflicts of interest.

## Supporting information




**Supporting File 1**: Experimental details, NMR spectra of all described compounds, NMR titration experiments, ITC data, DFT calculations data are given. Deposition Number(s) 2480349, (for TP[3]CH_3_), 2480350 (for TP[2]COOEt), 2480351 (for TP[8]CH_3_), 2480352 (for TP[3]COOEt) and 2494955 (for TP[2]CH_3_) contain the supplementary crystallographic data for this paper. These data are provided free of charge by the joint Cambridge Crystallographic Data Centre and Fachinformationszentrum Karlsruhe http://www.ccdc.cam.ac.uk/structures.

**Supporting File 2**: anie71719‐sup‐0002‐Data.zip.

## Data Availability

The data that support the findings of this study are available in the supplementary material of this article.
