# Supplementary material for: o‐Terphenyl‐Based Family of Conjugated Macrocycles: Selective Recognition of Phenylalanine in Water and Interaction With Insulin
Source: Angew Chem Int Ed Engl. 2026 Mar 18;65(24):e25972. doi: 10.1002/anie.202525972 (PMC13245601; doi:10.1002/anie.202525972)
Supplement: Supplementary file 1 — Supporting File 1: Experimental details, NMR spectra of all described compounds, NMR titration experiments, ITC data, DFT calculations data are given. Deposition Number(s) 2480349, (for TP[3]CH3), 2480350 (for TP[2]COOEt), 2480351 (for TP[8]CH3), 2480352 (for TP[3]COOEt) and 2494955 (for TP[2]CH3) contain the supplementary crystallographic data for this paper. These data are provided free of charge by the joint Cambridge Crystallographic Data Centre and Fachinformationszentrum Karlsruhe http://www.ccdc.cam.ac.uk/structures. [file ANIE-65-e25972-s001.pdf]

Supporting Information

Table of Contents

1. Materials and Methods 2

2. Synthesis 3

3. NMR Spectra 8

4. Mass Spectra 27

5. 1H NMR Titrations 32

6. Thioflavin T Assay 39

7. Isothermal Titration Calorimetry (ITC) 43

8. Crystal Data and Experimental 46

9. DFT Calculations 46

8. References 55

1. Materials and Methods

All chemicals and solvents were purchased from commercial sources (Sigma-Aldrich, abcr, Fisher Scientific, TCI and Chem. Purr.) and used without any further purification. All reactions as stated in the experimental procedure were performed in anhydrous solvents. All reactions were carried out in oven-dried glassware. Thin-layer chromatography (TLC) was performed on Merck silica gel 60 F254 pre-coated plastic-backed plates, which were visualized by ultraviolet light (254 and 350 nm).

Flash column chromatography was performed on Macherey–Nagel silica gel; solid deposit on Celite® 60 M (230–400 mesh, 0.04–0.063 mm).

NMR spectra were recorded on a Bruker Avance 400 at 400 MHz (^{1}H NMR) and 100 MHz (^{13}C NMR) and Bruker 600 MHz (^{1}H NMR) and 151 MHz (^{13}C NMR), respectively. The signals were referenced to residual solvent peaks (in parts per million (ppm) ^{1}H: $CDCl_3$, 7.26 ppm; $(CD_3)_2SO$, 2.50 ppm; ^{13}C: $CDCl_3$, 77.16 ppm; $(CD_3)_2SO$, 39.52 ppm). Coupling constants were assigned as observed. The obtained spectra were evaluated with the program MestReNova.

Mass spectra were recorded on a Bruker ESI TOF maXis 4G instrument. APPI-ToF mass spectrometry was performed on a Bruker maXis 4G UHR TOF MS/MS-spectrometer. The data were evaluated using the Bruker Compass Data Analysis 4.2 program.

UV/vis spectroscopy was performed using a Varian Cary 5000 UV/Vis-NIR spectrometer. The spectra were measured in the indicated solvents in quartz cuvettes (edge length = 1 cm) at room temperature. The absorption maxima λ_{max} are given in nanometers [nm].

Fluorescence spectroscopy was recorded on a FluoroMax 4 from Horiba equipped with a 150W ozone free Xe arc lamp with temperature control. The samples were measured in 1 cm quartz cuvettes (Hellma) at room temperature. Emission maxima λmax are given in nanometer [nm] with relative intensities in [%]. All titrations were done by keeping the host concentration constant and adding an increasing quantity of guests. The resulting data were imported into the *HypSpec* program and fitted to obtain stability constants.

Circular Dichroism (CD) was recorded on J-815 CD spectropolarimeter from JASCO equipped with a 150W Xe arc lamp with temperature control. The spectra were measured in quartz cuvettes (path length = 1 cm) at room temperature in the indicated solvents.

Isothermal Titration Calorimetry (ITC) experiments were carried out at 298 K on a nano–ITC calorimeter from TA Instruments. The resulting data were imported into the *Nanoaznalyze* program and fitted into *Affinometer* program to obtain stability constants.

2. Synthesis

2.1 General synthesis procedures

A. Synthesis of di-iodo derivative

The di-iodo derivatives of **1** (o-xylene) and **2** (diethyl phthalate) were synthesized according to the literature method without any modifications [1-2].

B. Synthesis of 1,1':2',1''-terphenyl derivative [TP]

In a 250 mL round-bottom flask equipped with a stirring bar, the di-iodo-derivative (2.0 g, 5.6 mmol), 3-bromo phenylboronic acid (2.50 g, 12.3 mmol), Pd(dppf)Cl_2 (410 mg, 0.56 mmol), and K_3PO_4 (4.75 g, 22.4 mmol) were dissolved in toluene: water (1:1, 240 mL). A condenser was attached, and this mixture was then heated at 80 °C for 3 hours. After cooling to room temperature, the solvent was removed on a rotary evaporator. The residue was suspended in 30 mL of water and extracted with dichloromethane (3×50 mL). The combined organic fractions were dried over anhydrous Na_2SO_4, filtered, and concentrated in vacuo. The crude product was purified by column chromatography (silica gel using hexane/ethyl acetate 9:1-8:2) to afford the desired product as a white solid.

C. Synthesis of Macrocycles [TP(n)]

A flame-dried 25-mL microwave vial was charged with bis(1,5-cyclooctadiene)nickel(0) (1.30 g, 4.8 mmol), 1,5-cyclooctadiene (0.6 mL, 4.8 mmol), and 2,2'-bipyridine (750 mg, 4.8 mmol). Anhydrous THF (15 mL) was added, and the resulting mixture was stirred at room temperature for 15 min under an argon atmosphere. A solution of the di-bromo derivative (500 mg, 1.20 mmol) in anhydrous THF (5 mL) was added dropwise over a period of 10 min. The reaction flask was sealed, and the mixture was stirred at 60 °C for 48 h. After cooling to room temperature, the solvent was removed under reduced pressure. The residue was dissolved in dichloromethane (DCM, 20-30 mL) and filtered to remove insoluble inorganic salts. The solid residue was washed with additional portions of DCM. The combined filtrates were concentrated in vacuo to afford the crude product.

D. Synthesis of acid macrocycles from esters [TP(n)COOH]

To a 25 mL microwave vial with a magnetic stir bar was added the ester derivative (25 mg, 34 μmol) and THF (5 mL). A 10% aqueous NaOH solution (5 mL) was then introduced, and the vial was capped. The mixture was stirred at 60 °C for 15 h in a sand bath. After the reaction was complete, the solvent was removed under reduced pressure. The remaining aqueous solution was acidified with 5 M HCl, resulting in the formation of a white precipitate. The precipitate was collected via centrifugation, washed with water (3×10 mL), and dried to afford the desired carboxylic acid derivative as a white solid.

E. Synthesis of acid macrocycles from methyl derivatives [TP(n)COOH]

The compounds were synthesized by a modified reaction reported earlier[3]. To a 25 mL round-bottom flask equipped with a magnetic stir bar and a reflux condenser, methyl derivative (51 mg, 0.8 mmol) was dissolved in a 1:1 v/v mixture of tBuOH/H2O (10 mL). KMnO4 (126 mg, 0.8 mmol) was added, and the resulting mixture was heated under reflux until the purple colour

of the permanganate solution disappeared. Subsequently, EtOH (10 mL) was added to quench any remaining KMnO4. The reaction mixture was then hot filtered to remove the brown manganese dioxide solids. After the filtrate cooled to room temperature, it was acidified with 2 M HCl, and the solvent was removed under reduced pressure. The resulting precipitate was collected by centrifugation, washed with deionized water (3×10 mL), and dried to afford the desired carboxylic acid derivative as a white solid (78% yield).

2.2 Synthesis of TP-CH_3 (3) and TP-COOEt (4) derivatives

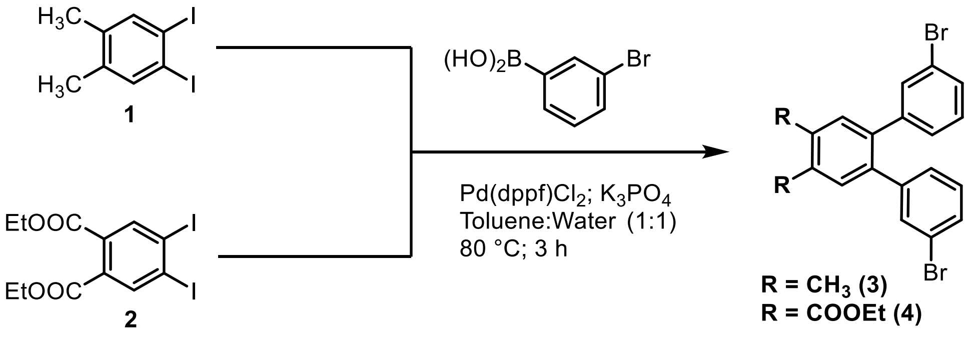

Scheme 1. Synthesis of TP-CH3 ***(3)*** *and TP-COOEt* ***(4)***.

2.3 3,3''-dibromo-4',5'-dimethyl-1,1':2',1''-terphenyl (3)

Compound **3** was synthesized by general procedure **A**. white solids 1.61 g (69%) Yield

^{1}H NMR (400 MHz, Chloroform-d) δ 7.37 (t, J = 1.8 Hz, 2H), 7.34 (ddd, J = 7.9, 2.0, 1.1 Hz, 2H), 7.18 (s, 2H), 7.05 (t, J = 7.8 Hz, 2H), 6.95 (dt, J = 7.7, 1.3 Hz, 2H), 2.35 (s, 6H).

^{13}C NMR (101 MHz, Chloroform-d) δ 143.33, 136.80, 136.58, 132.65, 131.92, 129.65, 129.47, 128.87, 122.21, 19.55.

HRMS (ESI, DCM) *m/z* calcd. for $C_{20}H_{16}Br_2$ 415.9598 obs. $[M]^+$ 415.9599.

2.4. diethyl 3,3''-dibromo-[1,1':2',1''-terphenyl]-4',5'-dicarboxylate (4)

Compound **4** was synthesized by general procedure **A**. white solids 1.62 g (72%) Yield

^{1}H NMR (400 MHz, Chloroform-d) δ 7.75 (s, 2H), 7.42 – 7.39 (m, 2H), 7.37 (t, J = 1.7 Hz, 2H), 7.10 (t, J = 7.8 Hz, 2H), 6.99 – 6.95 (m, 2H), 4.43 – 4.38 (m, 4H), 1.39 (t, J = 7.2 Hz, 6H).

^{13}C NMR (101 MHz, Chloroform-d) δ 167.17, 141.75, 141.32, 132.45, 131.89, 131.19, 130.88, 129.84, 128.54, 122.55, 62.05, 14.28.

HRMS (ESI, DCM) *m/z* calcd. for $C_{24}H_{20}Br_2O_4$ 529.9728 obs. $[M]^+$ 529.9458.

2.5. Macrocycles dimethyl-1,1':2',1''-terphenyl (5)

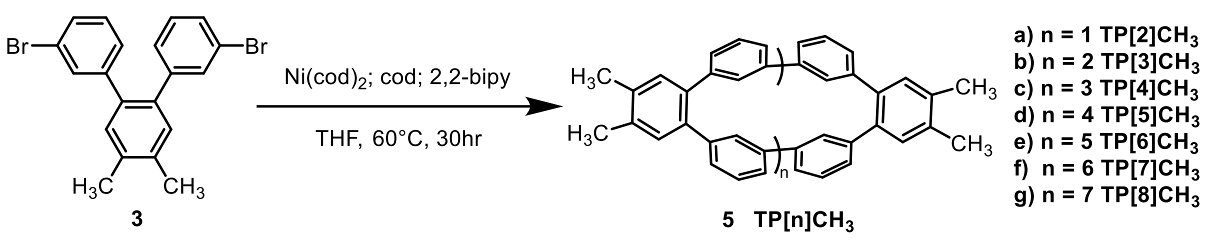

Scheme 2. Synthesis of TP[n]CH3 ***(5)****.*

TP[n]CH$_3$ were synthesized by general procedure **C**. All eight macrocycles were purified by flash chromatography on silica gel [silica cartridge (100 g); solid deposit on Celite®; λ detection (224 nm, 270 nm)]; gradient hexane: dichloromethane (8:2-5:5) for 120 min at 60 mL/min] isolated all macrocycles as a white solid.

5a) TP[2]CH$_3$ (65 mg) yield 21%. ^{1}H NMR (400 MHz, Chloroform-d) δ 7.31 (m, 4H), 7.27 (m, 4H), 7.25 – 7.24 (m, 4H), 7.14 (dt, J = 7.0, 1.8 Hz, 4H), 7.06 (m, 4H), 2.37 (s, 12H). ^{13}C NMR (101 MHz, Chloroform-d) δ 141.09, 140.36, 137.92, 136.17, 131.60, 131.44, 128.80, 128.09, 124.57, 19.60. HRMS appi+: *(m/z)* calcd. for $C_{40}H_{32}^+$ $[M]^+$ 512.2504, found 512.2485.

5b) TP[3]CH$_3$ (65 mg) yield 21%. ^{1}H NMR (400 MHz, Chloroform-d) δ 7.19 (t, J = 3.7 Hz, 12H), 7.14 (d, J = 7.2 Hz, 6H), 7.08 (d, J = 7.5 Hz, 6H), 6.58 (m, 6H), 2.46 (s, 18H). ^{13}C NMR (101 MHz, Chloroform-d) δ 142.52, 141.18, 138.72, 136.65, 132.93, 130.05, 129.51, 129.33, 125.91, 20.63. HRMS appi+: *(m/z)* calcd. for $C_{60}H_{49}^+$ $[M+H]^+$ 769.3834, found 769.3847.

5c) TP[4]CH$_3$ (43 mg) yield 14%. ^{1}H NMR (400 MHz, Chloroform-d) δ 7.18 (s, 8H), 6.97 (s, 16H), 6.83 (s, 8H), 2.35 (s, 24H). ^{13}C NMR (101 MHz, Chloroform-d) δ 141.69, 141.11, 137.97, 135.97, 131.88, 129.60, 128.54, 128.45, 125.23, 19.61. HRMS appi+: *(m/z)* calcd. for $C_{80}H_{65}^+$ [M+H]+ 1025.5086, found $[M]^+$ 1025.5061.

5d) TP[5]CH$_3$ (25 mg) yield 8%. ^{1}H NMR (400 MHz, Chloroform-d) δ 7.11 (s, 10H), 7.06 (s, 10H), 6.93 (dt, J = 21.6, 7.4 Hz, 30H), 2.29 (s, 30H). ^{13}C NMR (101 MHz, Chloroform-d) δ 141.78, 140.92, 137.95, 135.82, 131.82, 129.16, 128.66, 128.29, 125.11, 19.60. HRMS appi+ *(m/z)*: calcd. for $C_{100}H_{82}^+$ $[M-2H]^+$ 1282.6416, found $[M]^+$ 1282.6391.

5e) TP[6]CH$_3$ (6 mg) yield 2%. ^{1}H NMR (601 MHz, Chloroform-d) δ 7.08 (s, 10H), 7.06 (s, 10H), 6.99 – 6.94 (m, 15H), 6.91 (t, J = 7.5 Hz, 25H), 2.25 (s, 36H). ^{13}C NMR (151 MHz, Chloroform-d) δ 141.78, 140.91, 137.89, 135.81, 131.86, 129.12, 128.66, 128.24, 125.09, 19.57. HRMS appi+ (m/z): calcd. for $C_{120}H_{96}^+$ $[M]^+$ 1537.7512, found $[M]^+$ 1537.7554.

5f) TP[7]CH$_3$ (3 mg) yield 1%. % ^{1}H NMR (500 MHz, Chloroform-d) δ 7.15 – 7.08 (m, 30H), 6.98 – 6.90 (m, 41H), 2.27 (s, 42H). ^{13}C NMR (126 MHz, Chloroform-d) δ 141.78, 140.90, 137.96, 135.87, 131.86, 129.04, 128.75, 128.25, 125.12, 19.56. HRMS appi+ (m/z): calcd. for $C_{140}H_{112}^+$ $[M]^+$ 1793.8797, found $[M+K]^+$ 1832.8422.

5g) TP[8]CH$_3$ (1.5 mg) yield 0.5%. ^{1}H NMR (400 MHz, Chloroform-d) δ 7.11 (d, J = 7.4 Hz, 35H), 6.97 – 6.89 (m, 45H), 2.25 (s, 48H). HRMS appi+: m/z calcd. for $C_{160}H_{128}^+$ $[M]^+$ 2049.0016 found $[M+K]^+$ 2088.9641.

2.6. Macrocycles [1,1':2',1''-terphenyl]-4',5'-dicarboxylate (6)

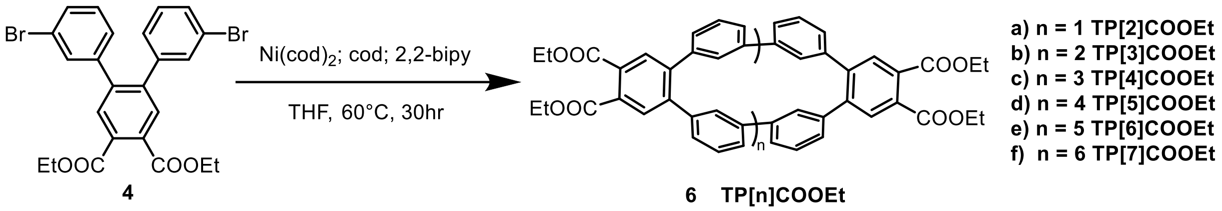

Scheme 3. Synthesis of TP[n]CH3 ***(3)*** *and TP[n]COOEt* ***(4)****.*

TP[n]COOEt were synthesized by general procedure **D**. All eight macrocycles were purified by flash chromatography on silica gel [silica cartridge (100 g); solid deposit on Celite®; λ detection (220 nm, 310 nm); gradient hexane: ethyl acetate (8:2-5:5) for 120 min at 60 mL/min] isolated all macrocycles as a white solid.

6a) TP[2]COOEt (89 mg) yield 20%. ^{1}H NMR (400 MHz, Chloroform-d) δ 7.89 (d, J = 0.9 Hz, 4H), 7.33 (t, J = 7.6 Hz, 4H), 7.29 – 7.18 (m, 14H), 7.08 (s, 4H), 4.41 (dt, J = 7.7, 6.6 Hz, 8H), 1.40 (td, J = 7.2, 0.9 Hz, 12H). ^{13}C NMR (101 MHz, Chloroform-d) δ 167.48, 143.09, 140.19, 139.37, 131.49, 131.08, 130.78, 129.29, 128.25, 125.68, 61.91, 14.31. *HRMS appi+:(m/z)*: calcd. for $C_{48}H_{40}O_8^+$ [M]+ 744.2723, found 744.2733.

6b) TP[3]COOEt (89 mg) yield 20%. ^{1}H NMR (400 MHz, Chloroform-*d*) δ 7.77 (s, 6H), 7.32 (t, *J* = 7.6 Hz, 6H), 7.17 (d, *J* = 7.4 Hz, 12H), 6.52 (s, 6H), 4.49 (q, *J* = 7.2 Hz, 12H), 1.47 (t, *J* = 7.2 Hz, 18H). ^{13}C NMR (101 MHz, Chloroform-*d*) δ 167.63, 143.01, 140.35, 139.90, 131.50, 131.30, 129.37, 128.89, 128.67, 126.25, 62.02, 14.41. HRMS MALDI *(m/z)*: calcd. for $C_{72}H_{60}O_{12}^+$ [M+H$^+$ 1116.4084, found 1116.5820.

6c) TP[4]COOEt (54 mg) yield 12%. ^{1}H NMR (601 MHz, Chloroform-*d*) δ 7.60 (s, 8H), 7.54 (dd, *J* = 6.6, 1.4 Hz, 8H), 7.29 (dt, *J* = 7.7, 1.3 Hz, 8H), 7.24 (t, *J* = 7.7 Hz, 8H), 7.16 (s, 8H), 7.13 (dt, *J* = 7.6, 1.3 Hz, 8H), 7.08 (t, *J* = 7.6 Hz, 8H), 6.42 (dt, *J* = 7.6, 1.1 Hz, 8H), 6.37 (t, *J* = 1.5 Hz, 8H), 5.98 (t, *J* = 1.6 Hz, 8H), 4.57 – 4.48 (m, 16H), 4.46 – 4.40 (m, 16H), 1.59 (t, *J* = 7.2 Hz, 24H), 1.43 (t, *J* = 7.1 Hz, 24H). ^{13}C NMR (151 MHz, Chloroform-*d*) δ 168.16, 166.33, 143.01, 142.45, 141.48, 141.26, 139.28, 138.05, 131.52, 130.28, 130.27, 130.23, 129.46, 129.11, 129.04, 128.19, 127.84, 127.30, 126.49, 126.46, 61.61, 14.54, 14.36. HRMS MALDI (m/z): calcd. for $C_{96}H_{80}O_{16}^+$ [M+H]$^+$ 1488.5446, found [M+Na]$^+$ 1512.7362.

6d) TP[5]COOEt (31 mg) yield 7%. ^{1}H NMR (400 MHz, Chloroform-*d*) δ 7.74 (s, 10H), 6.95 (d, *J* = 43.9 Hz, 40H), 4.41 (q, *J* = 7.1 Hz, 20H), 1.39 (t, *J* = 7.1 Hz, 30H). ^{13}C NMR (101 MHz, Chloroform-*d*) δ 167.43, 142.89, 140.85, 140.05, 131.47, 131.08, 129.20, 129.08, 128.58, 126.15, 61.93, 14.32. HRMS appi+ (m/z): calcd. for $C_{120}H_{100}O_{20}^+$ [M]$^+$ 1860.6808, found [M]$^+$ 1860.6828.

6e) TP[6]COOEt (4 mg) yield 1%. HRMS appi+ (m/z): calcd. for $C_{144}H_{120}O_{24}^+$ [M]$^+$ 2231.8169, found [M+Na]$^+$ 2256.8133.

6f) TP[7]COOEt (2 mg) yield 0.5%. HRMS ESI+ (m/z): calcd. for $C_{168}H_{140}O_{28}^+$ [M]$^+$ 2604.9531, found [M+Na]$^+$ 2628.9422.

2.7. Macrocycles [1,1':2',1''-terphenyl]-4',5'-dicarboxylate (6)

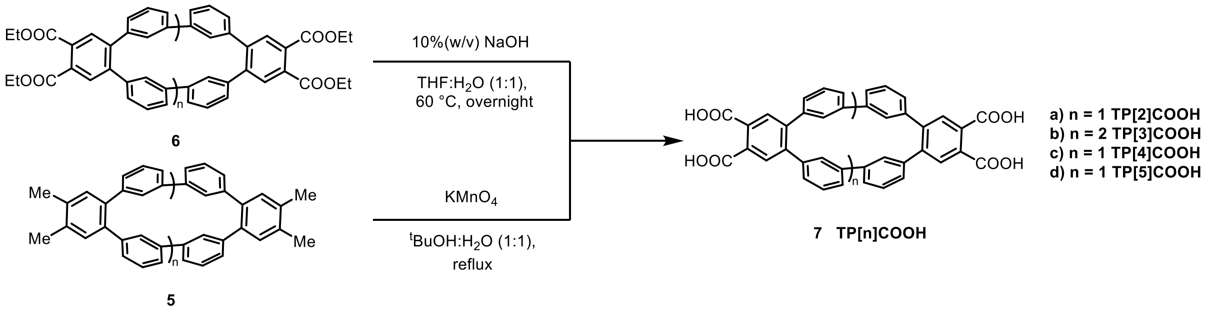

*Scheme 4. Synthesis of TP[n]COOH **(7)** from TP[n]CH$_3$ **(5)** and TP[n]COOEt **(6)**.*

TP[n]COOH were synthesized by general procedure **D**. The general procedure **E** used to synthesize the **TP[2]COOH** and **TP[3]COOH** the rest acid derivatives achieved in a lower yield.

7a) TP[2]COOH: obtained by general procedure **D** (17.44mg) yield 82.14% and by **E** (31.5mg) yield 51%.

^{1}H NMR (400 MHz, DMSO-d6) δ 7.72 (s, 1H), 7.45 (d, J = 7.9 Hz, 1H), 7.34 (t, J = 7.7 Hz, 1H), 7.19 (d, J = 7.5 Hz, 1H). ^{13}C NMR (101 MHz, DMSO) δ 168.21, 142.51, 139.56, 138.34, 131.85, 130.28, 129.92, 128.97, 127.70, 124.98. HRMS APPI+ *(m/z)*: calcd. for $C_{40}H_{23}O_8^-$ 631.1398, found [M]$^-$ 631.1372.

7b) TP[3]COOH: obtained by general procedure **D** (15.05mg) yield 80.54% and by **E** (11.72mg) yield 38%

^{1}H NMR (601 MHz, DMSO-d_6) δ 7.71 (s, 6H), 7.36 (t, J = 7.7 Hz, 6H), 7.21 (d, J = 7.5 Hz, 12H), 6.53 (s, 6H). ^{13}C NMR (151 MHz, DMSO) δ 167.95, 141.30, 139.33, 139.31, 132.21, 130.43, 128.97, 128.30, 127.71, 125.41. HRMS ESI- *(m/z)*: calcd. for $C_{60}H_{35}O_{12}^{2-}$ 473.1031, found [M]$^{2-}$ 473.1036, [M]$^{3-}$ 315.0663.

7c) TP[4]COOH: obtained by general procedure **D** (14.2mg) yield 80%.

^{1}H NMR (400 MHz, DMSO-d_6) δ 7.54 (d, J = 7.8 Hz, 8H), 7.45 (s, 8H), 7.28 (d, J = 7.8 Hz, 8H), 7.20 (t, J = 7.7 Hz, 8H), 7.10 (t, J = 7.7 Hz, 8H), 7.00 (d, J = 4.2 Hz, 16H), 6.38 (d, J = 7.8 Hz, 8H), 6.29 (s, 8H), 5.88 (s, 8H). ^{13}C NMR (151 MHz, DMSO-d6) δ 168.56, 166.93, 141.67, 141.06, 139.61, 138.01, 137.43, 132.29, 130.51, 129.20, 128.18, 127.57, 126.93, 126.78, 125.67, 125.43. HRMS ESI+ *(m/z)*: calcd. for $C_{80}H_{46}O_{16}^-$ 1262.2791, found [M]$^{2-}$ 631.1398, [M]$^{3-}$ 440.4241, [M]$^{4-}$ 315.0663.

7d) TP[5]COOH: obtained by general procedure **D** (9.8mg) yield 78%.

^{1}H NMR (601 MHz, DMSO-d6) δ 7.88 – 7.50 (m, 15H), 7.35 – 6.75 (m, 35H). ^{13}C NMR (151 MHz, DMSO-d6) δ 167.82, 141.45, 139.74, 139.50, 132.10, 130.34, 128.49, 128.14, 127.99, 125.37. HRMS ESI- *(m/z)*: calcd. for $C_{100}H_{58}O_{20}^-$ 1578.3527, found [M]$^{2-}$ 789.6802, [M]$^{3-}$ 526.1164, [M]$^{4-}$ 394.3355.

3. NMR Spectra

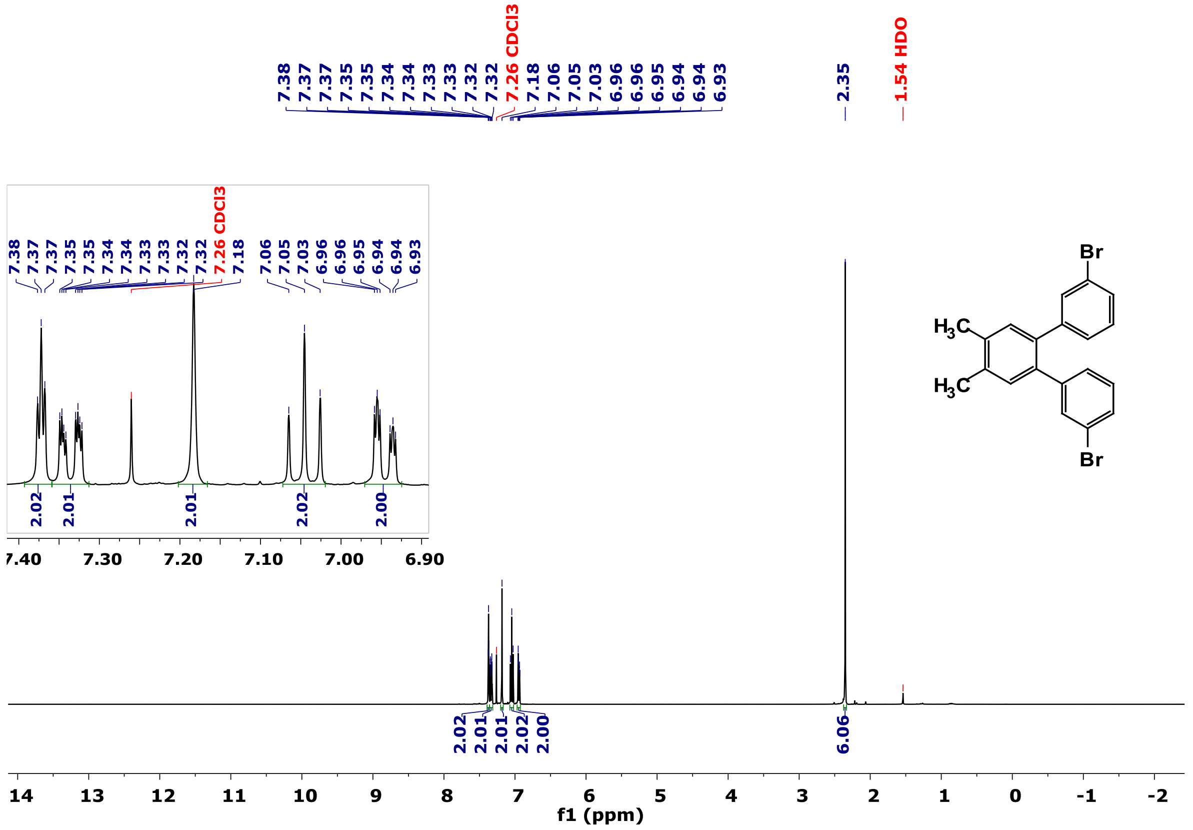

*Figure S1. ^{1}H NMR (400MHz, $CDCl_3$) spectrum of **3**.*

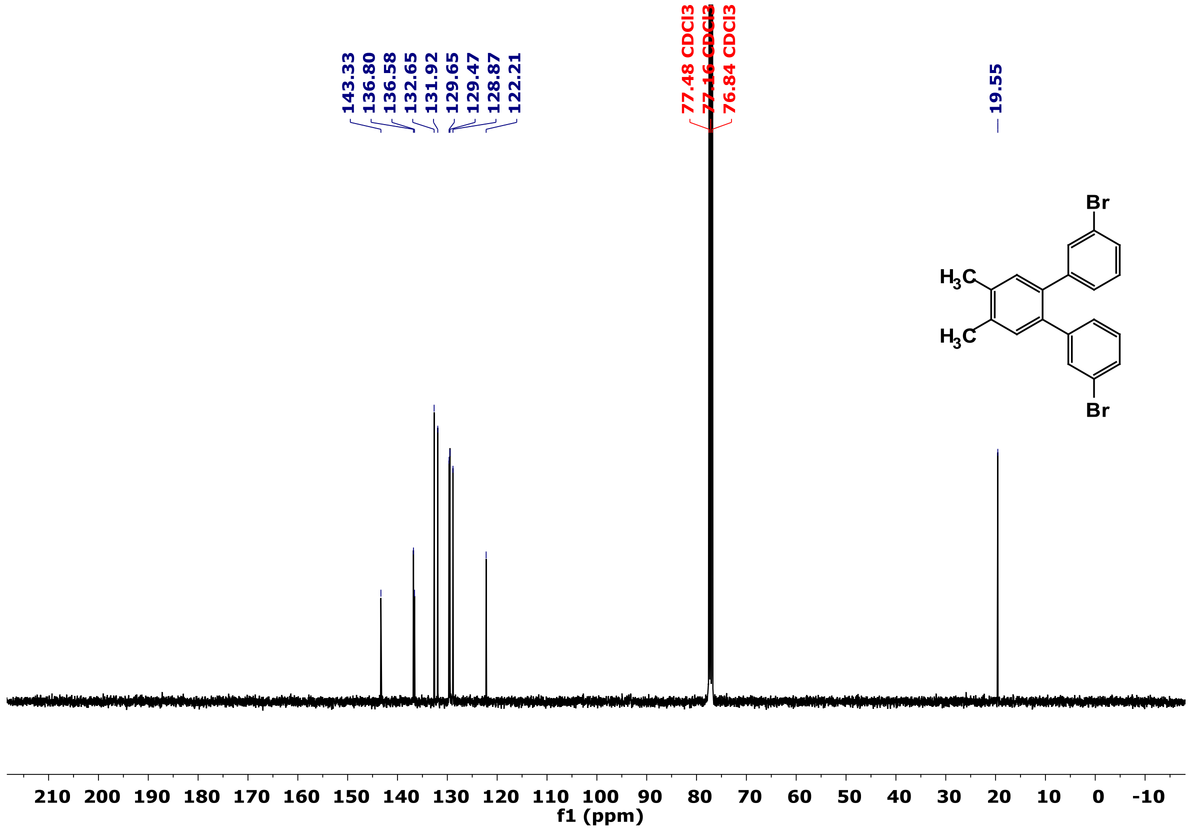

*Figure S2. ^{13}C NMR (101MHz, $CDCl_3$) spectrum of **3**.*

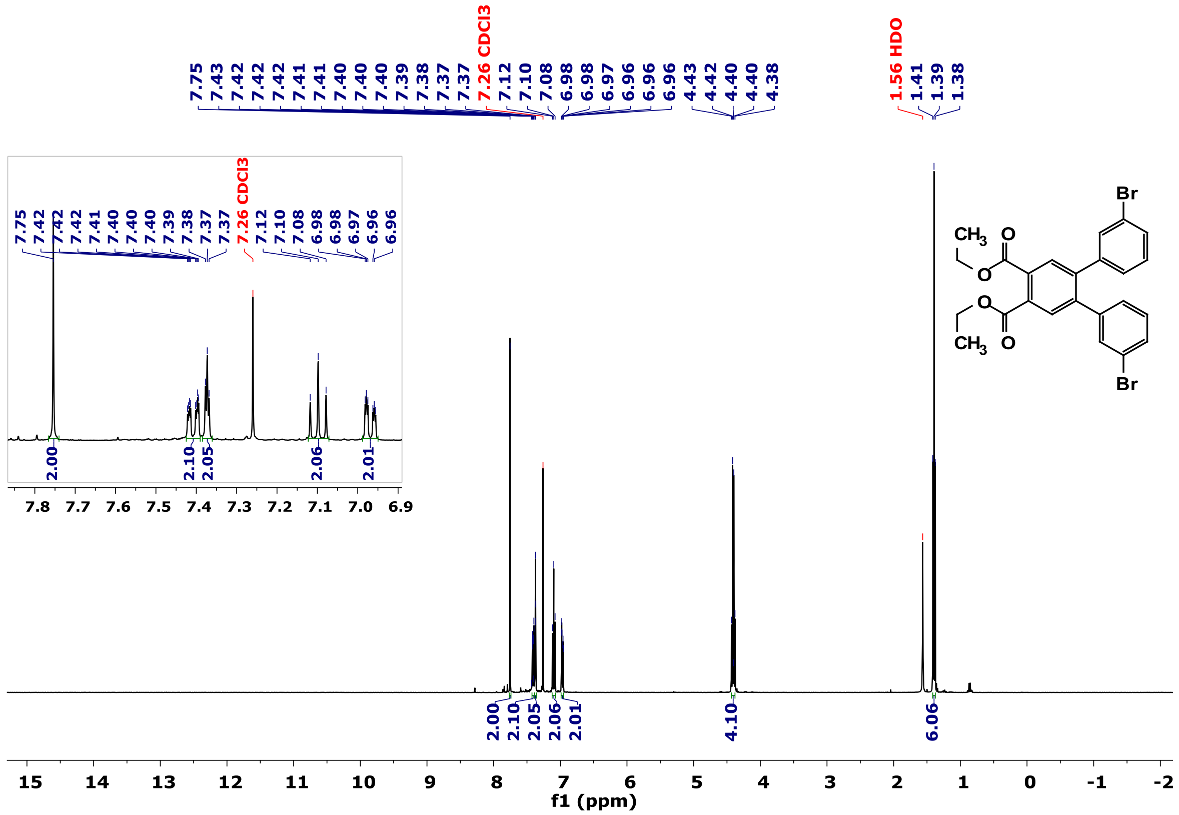

Figure S3. *^{1}H NMR (400MHz, CDCl$_3$) spectrum of* ***4***.

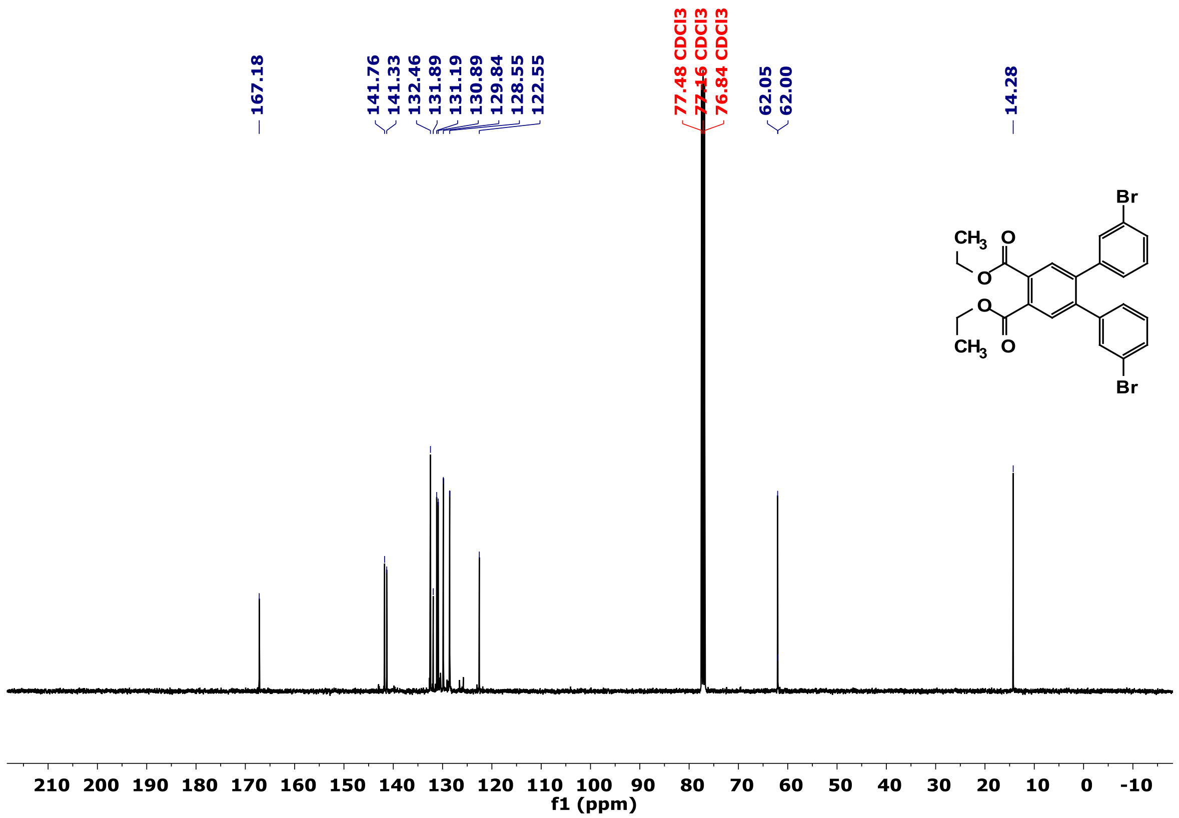

Figure S4. *^{13}C NMR (101MHz, CDCl$_3$) spectrum of* ***4***.

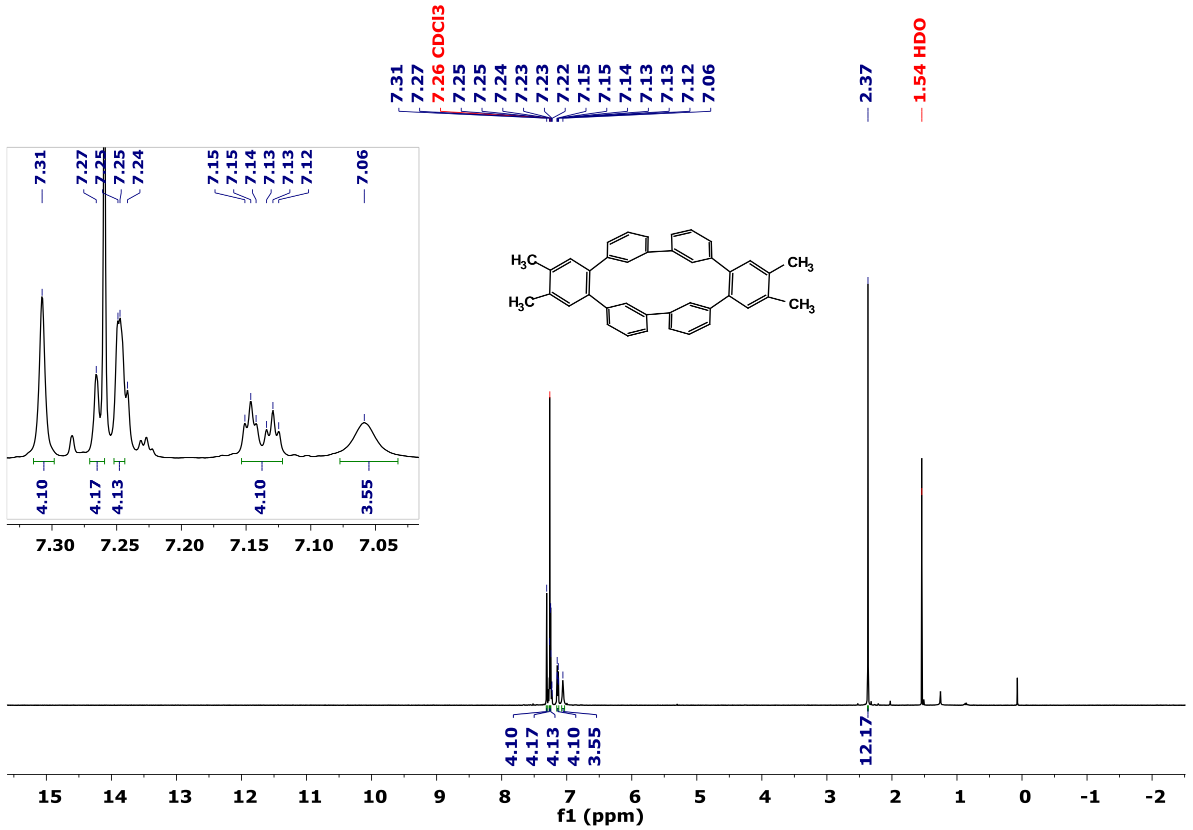

Figure S5. [1]*H NMR (400MHz, CDCl*$_3$*) spectrum of* ***TP[2]CH***$_3$.

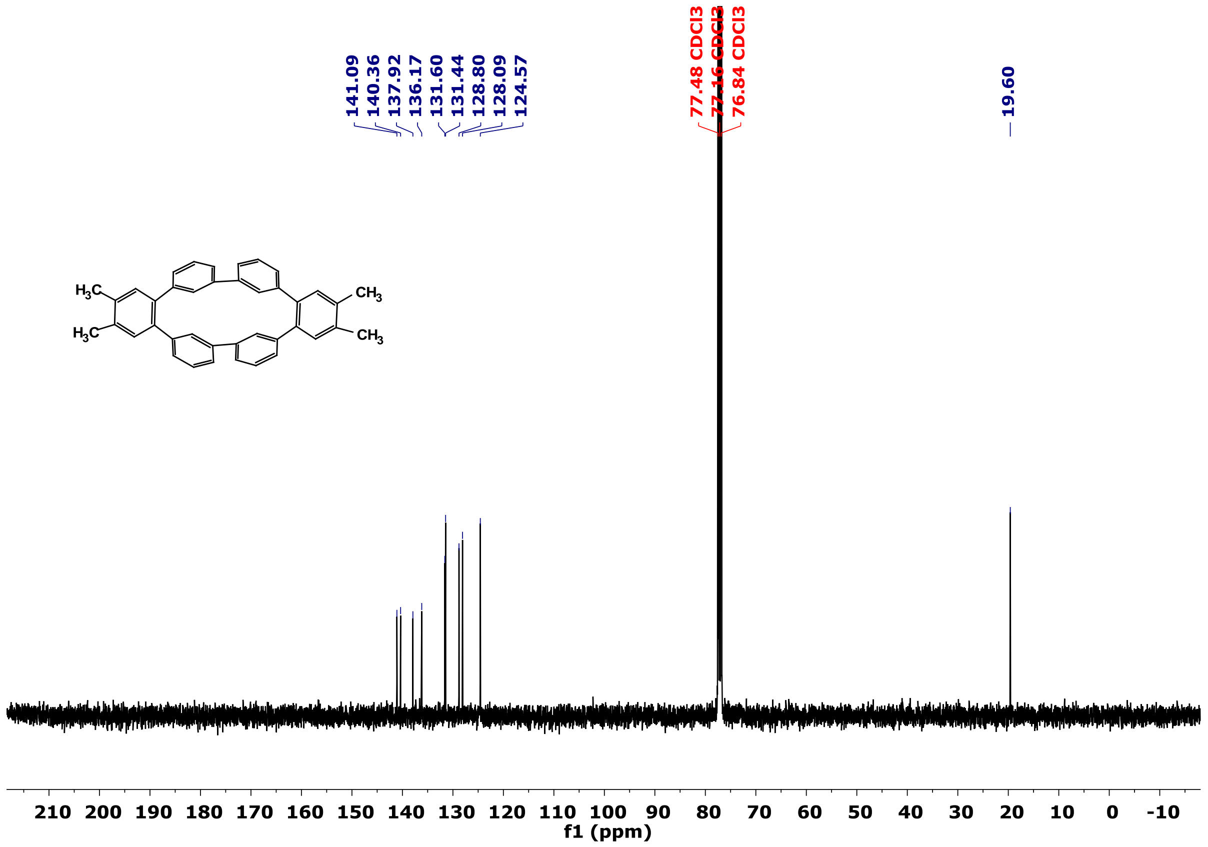

Figure S6. [13]*C NMR (101MHz, CDCl*$_3$*) spectrum of* ***TP[2]CH***$_3$.

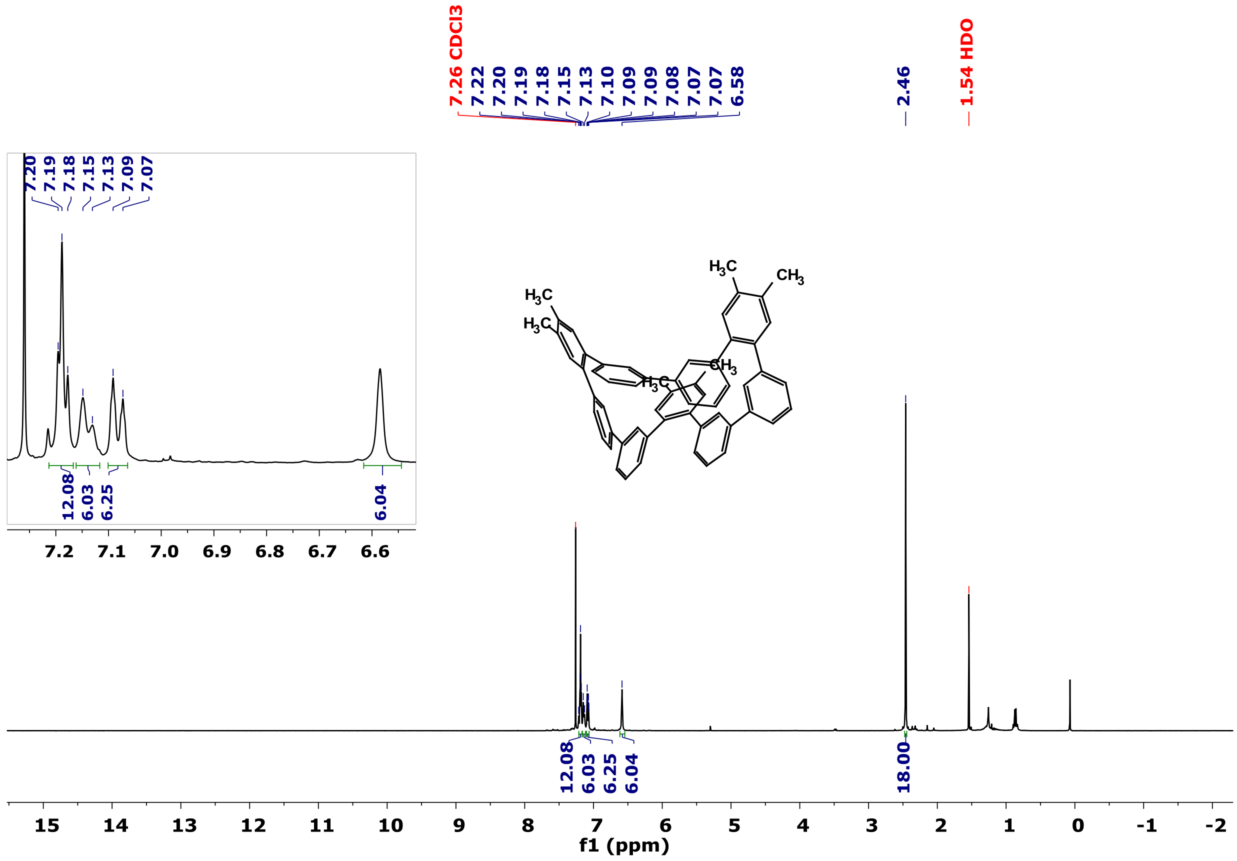

Figure S7. ¹*H NMR (400MHz, CDCl*$_3$*) spectrum of* ***TP[3]CH***$_3$.

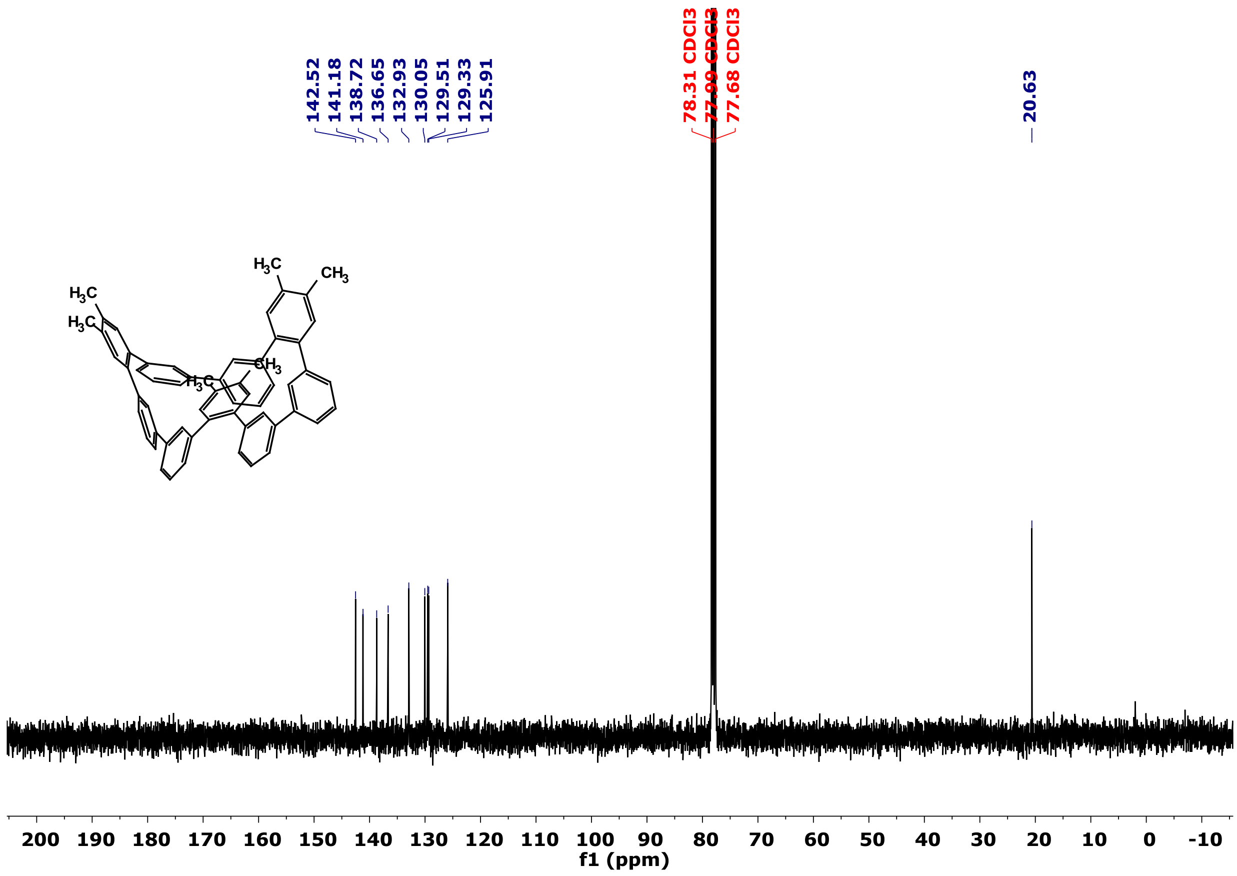

Figure S8. ¹³*C NMR (101MHz, CDCl*$_3$*) spectrum of* ***TP[3]CH***$_3$.

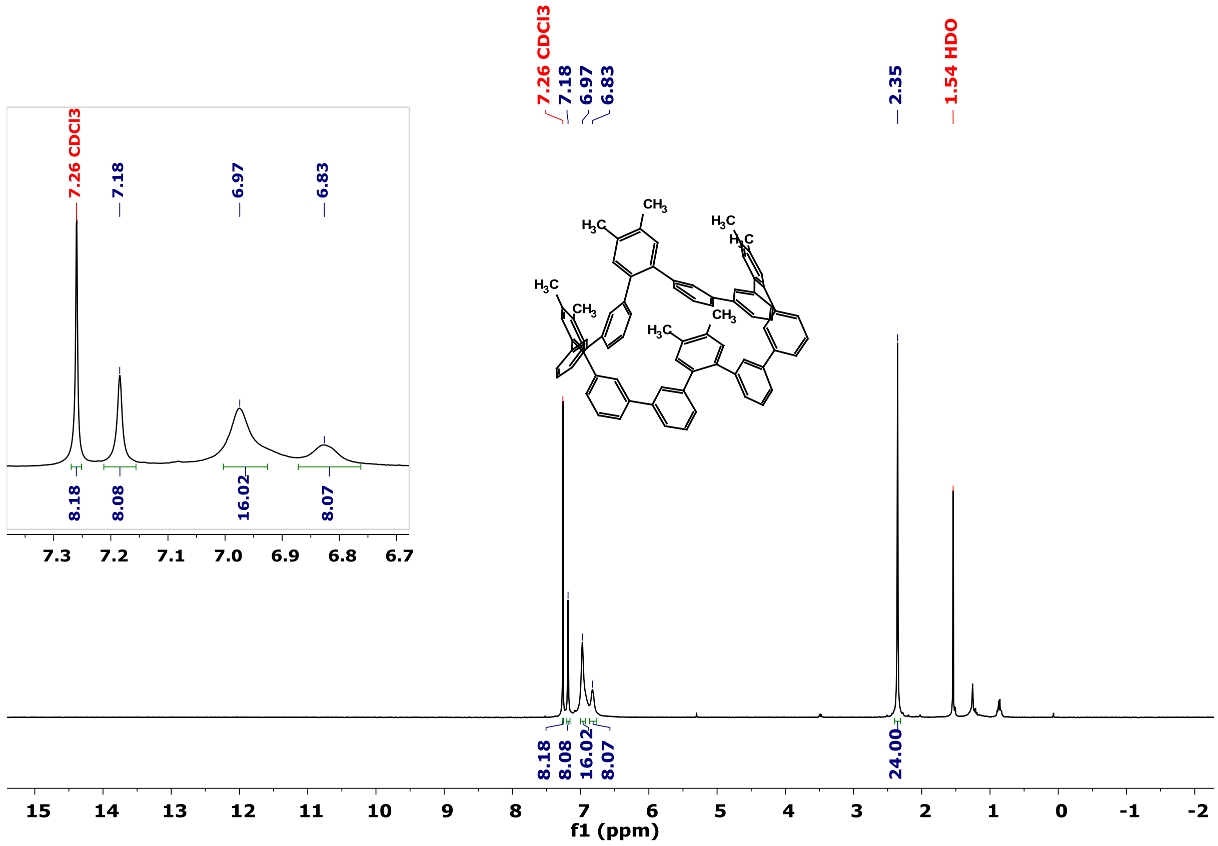

*Figure S9. ^{1}H NMR (400MHz, $CDCl_3$) spectrum of **TP[4]CH$_3$**.*

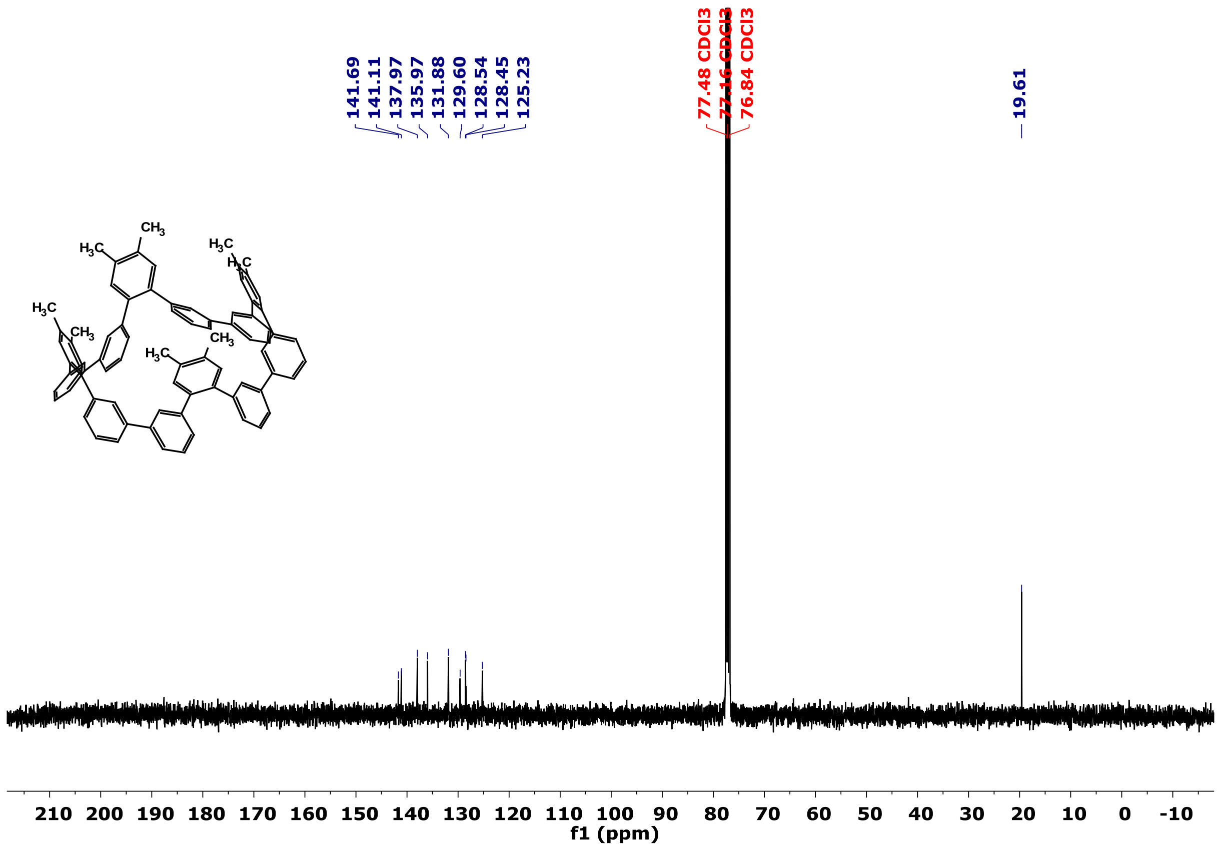

*Figure S10. ^{13}C NMR (101MHz, $CDCl_3$) spectrum of **TP[4]CH$_3$**.*

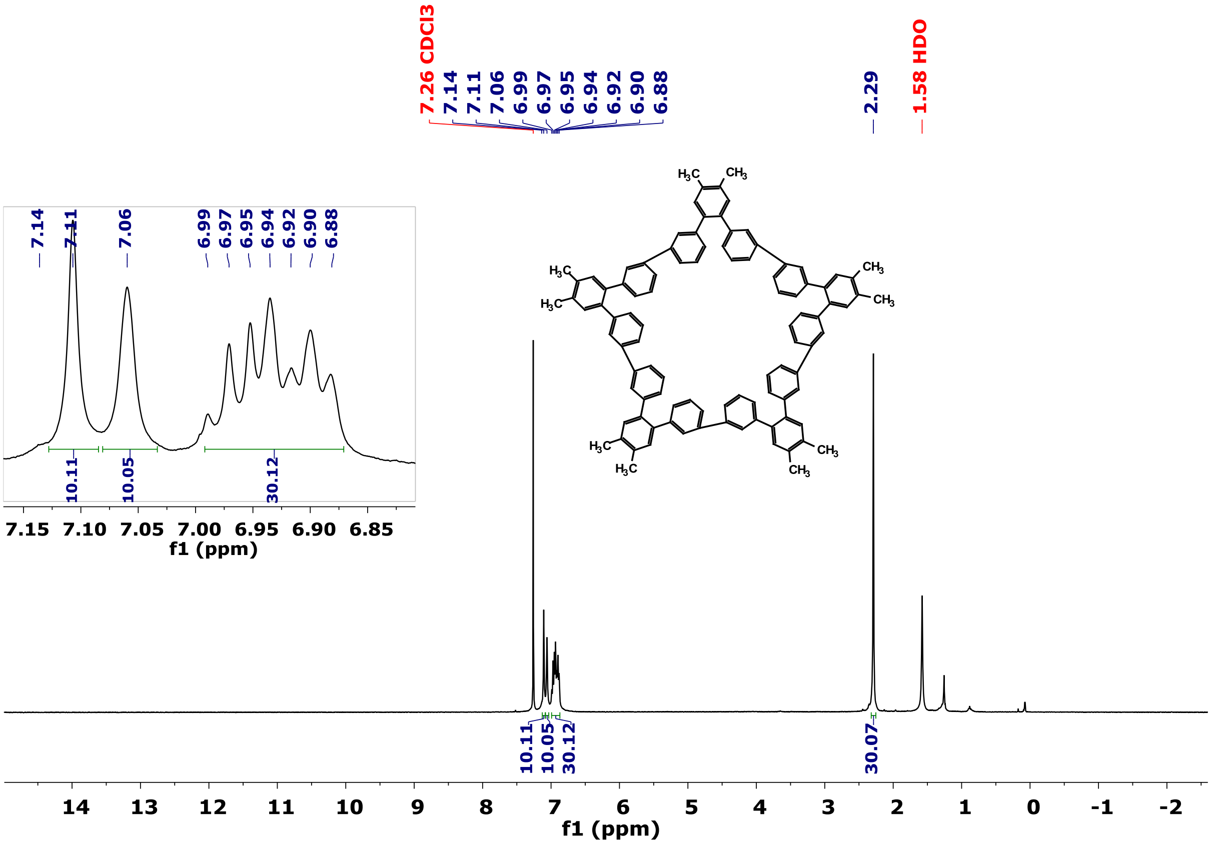

Figure S11. ¹H NMR (400MHz, $CDCl_3$) spectrum of ***TP[5]CH₃***.

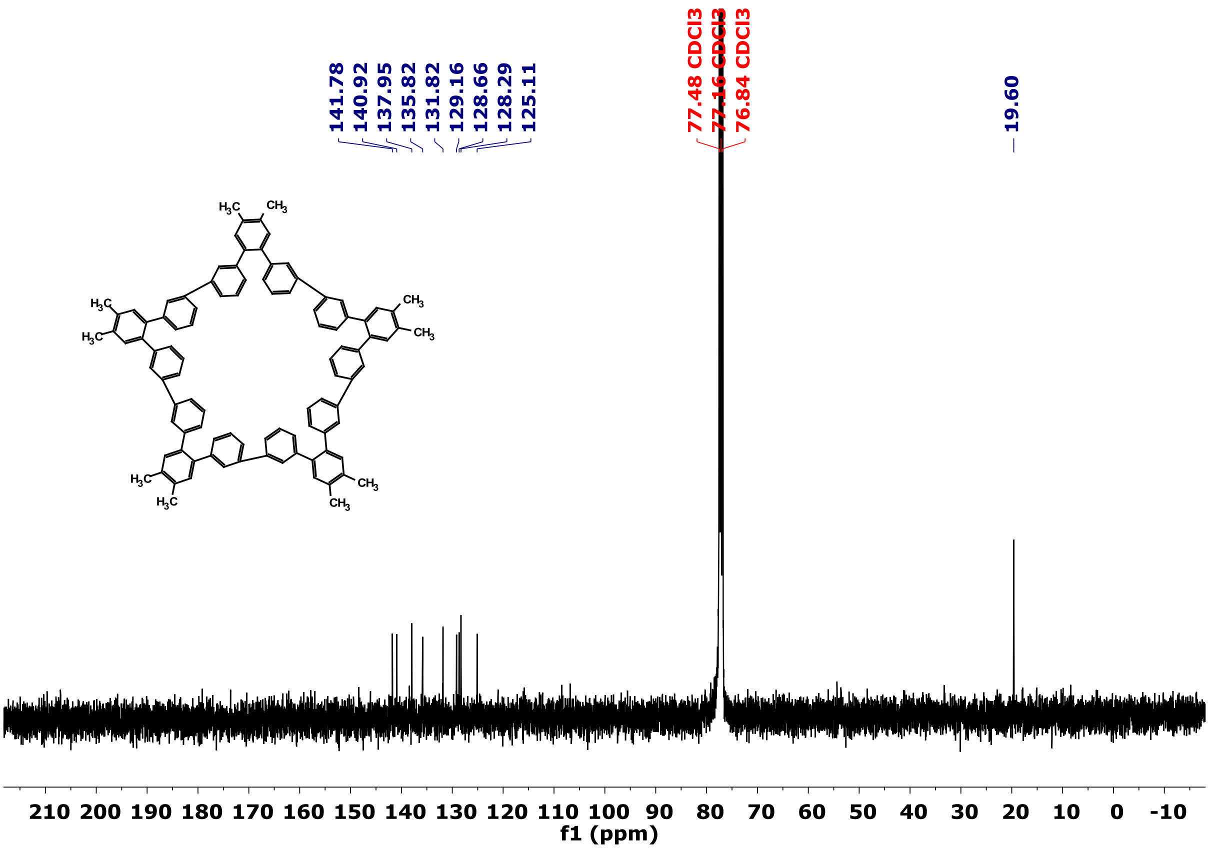

Figure S12. ¹³C NMR (101MHz, $CDCl_3$) spectrum of ***TP[5]CH₃***.

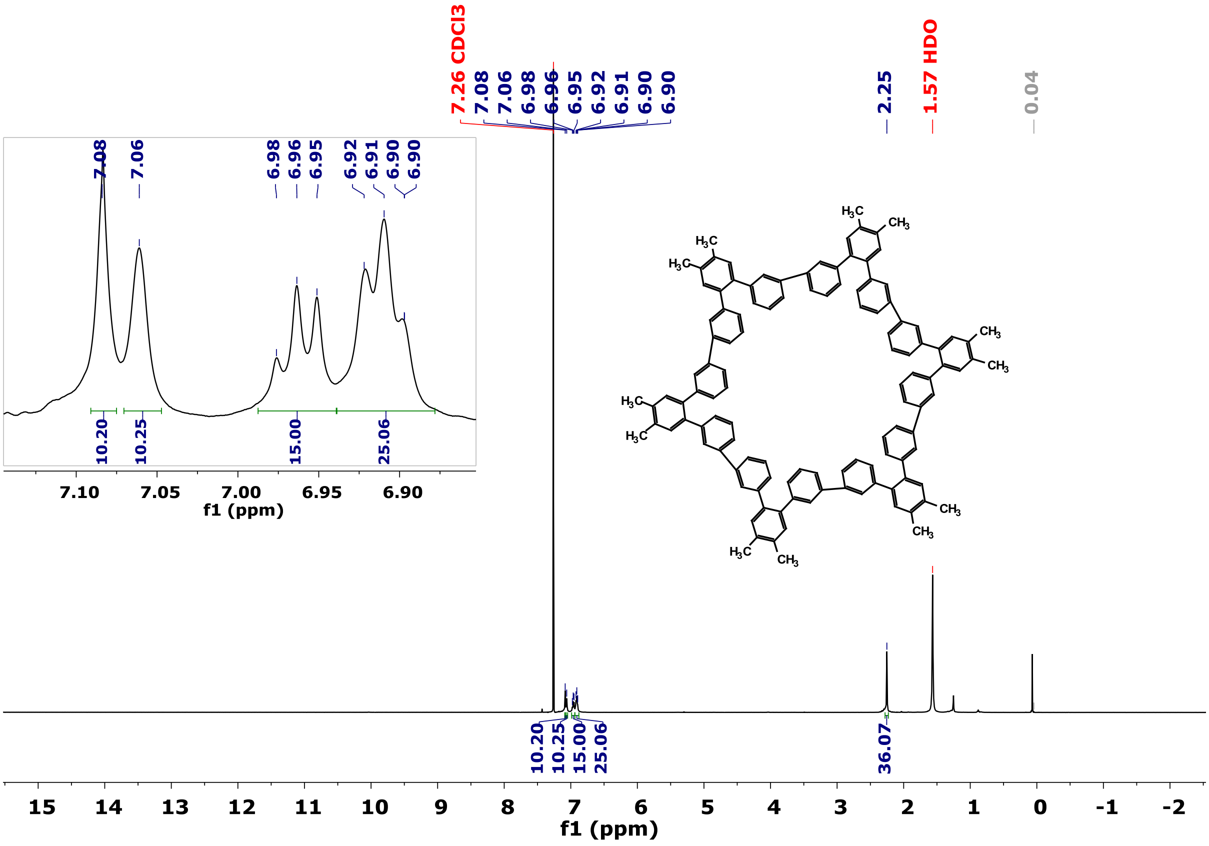

*Figure S13. ^{1}H NMR (601MHz, $CDCl_3$) spectrum of **TP[6]CH$_3$**.*

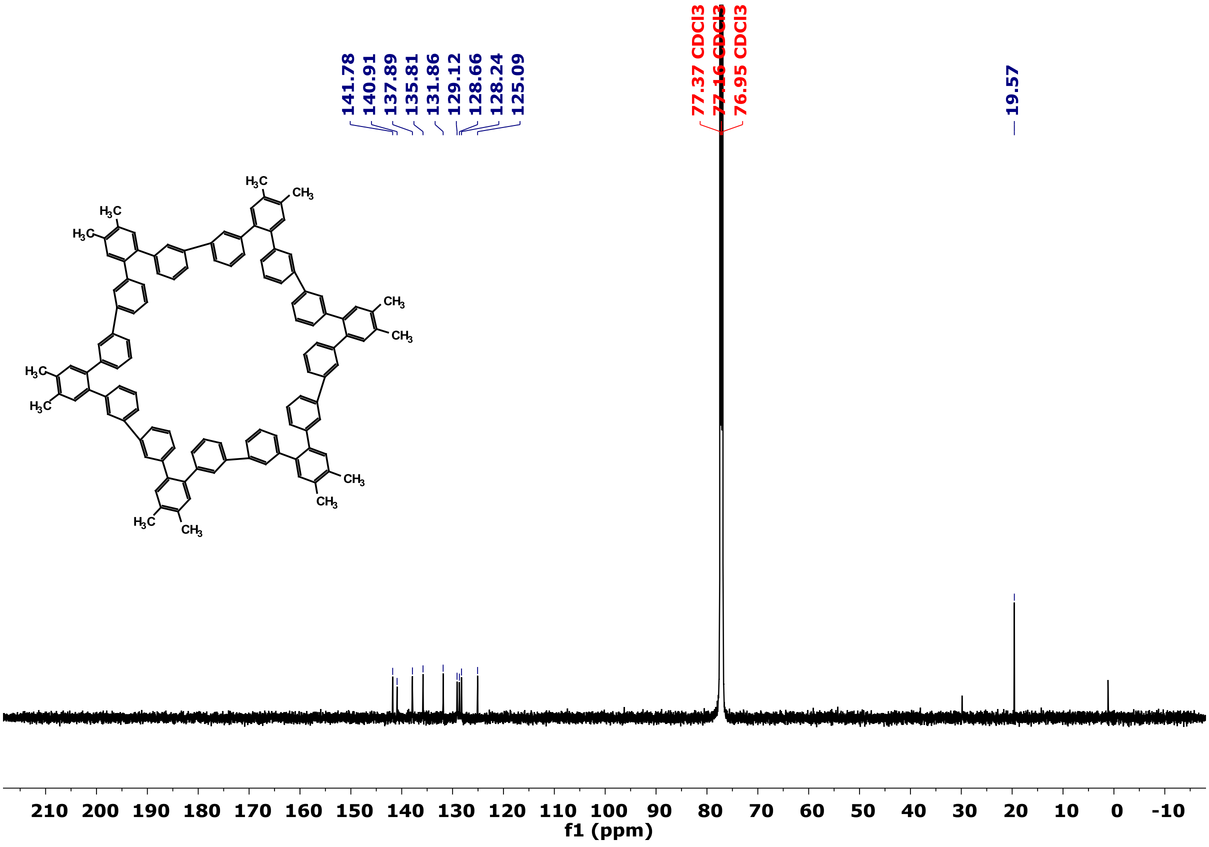

*Figure S14. ^{13}C NMR (151MHz, $CDCl_3$) spectrum of **TP[6]CH$_3$**.*

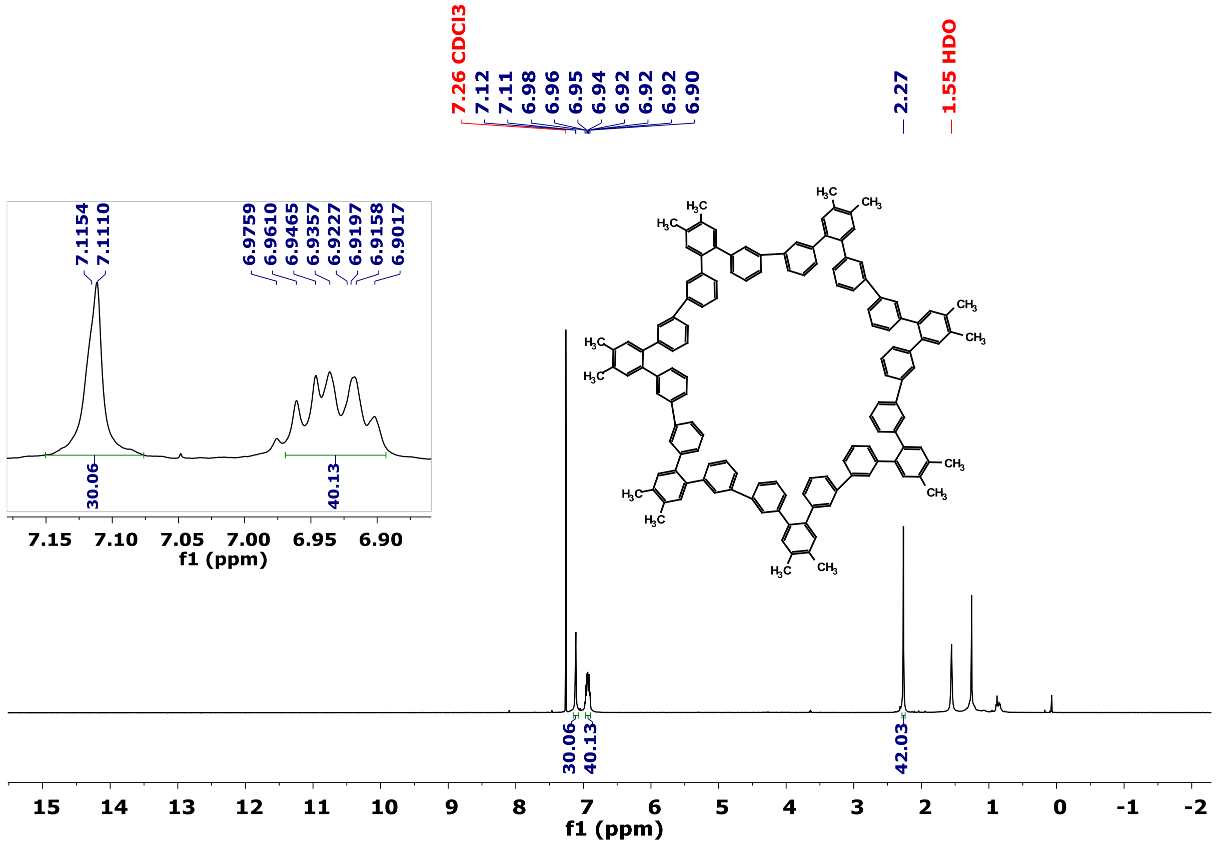

Figure S15. ^{1}H NMR (500MHz, $CDCl_3$) spectrum of ***TP[7]CH$_3$****.*

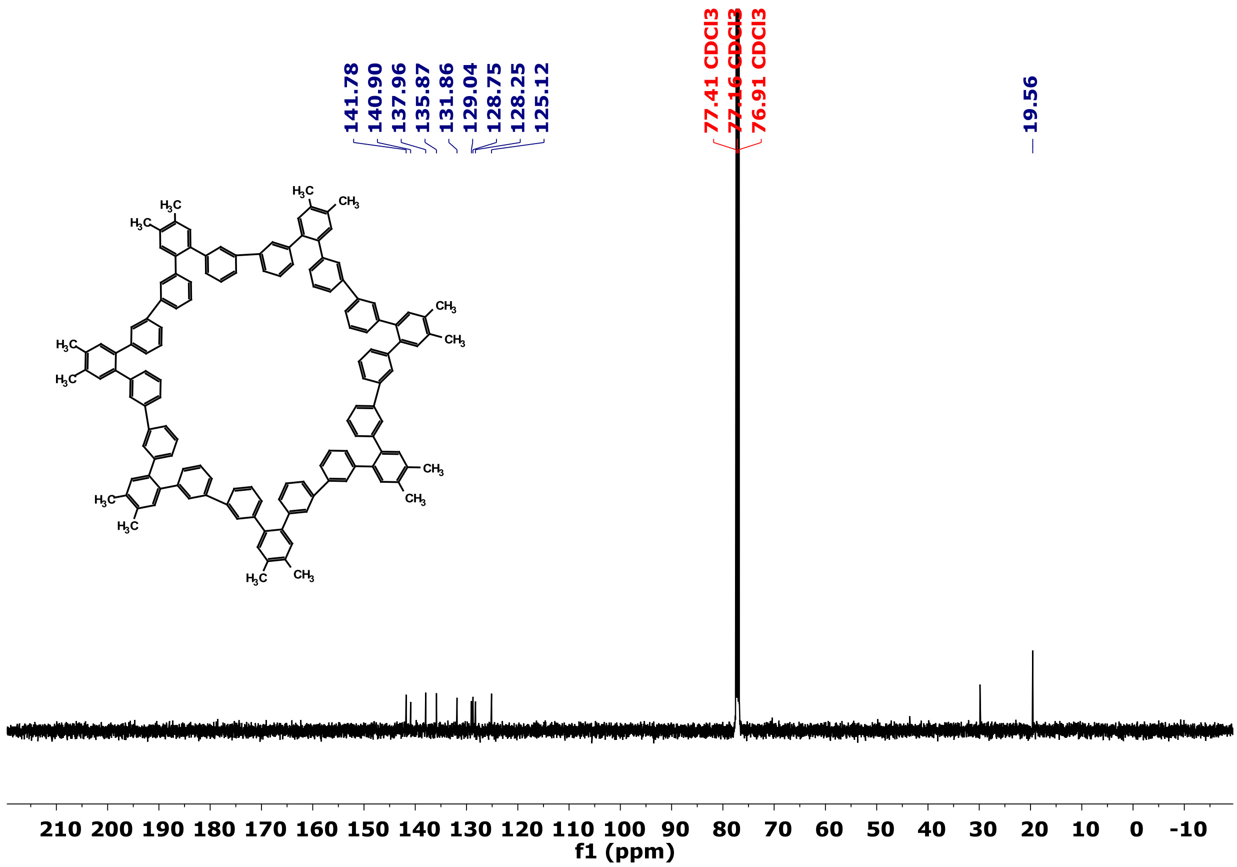

Figure S16. ^{13}C NMR (126MHz, $CDCl_3$) spectrum of ***TP[7]CH$_3$****.*

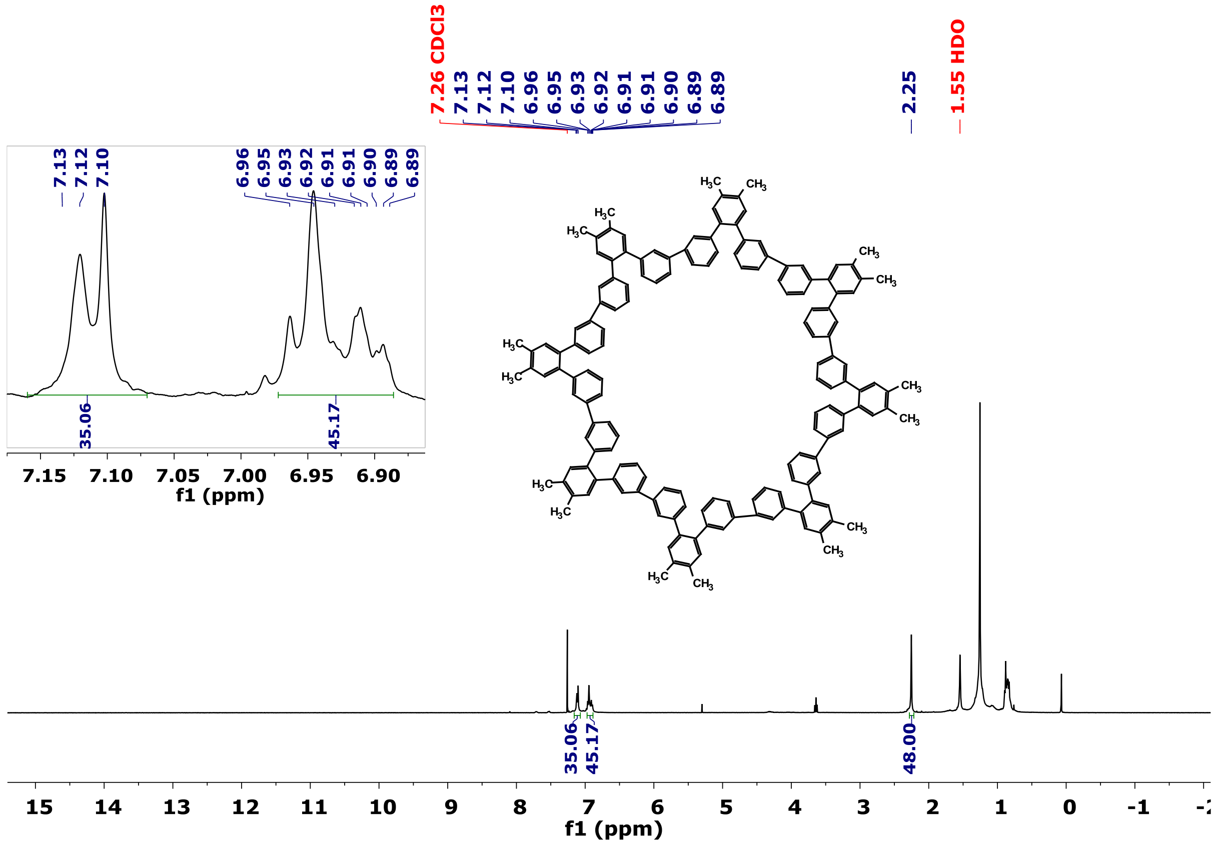

*Figure S17. ^{1}H NMR (400MHz, $CDCl_3$) spectrum of **TP[8]CH$_3$**.*

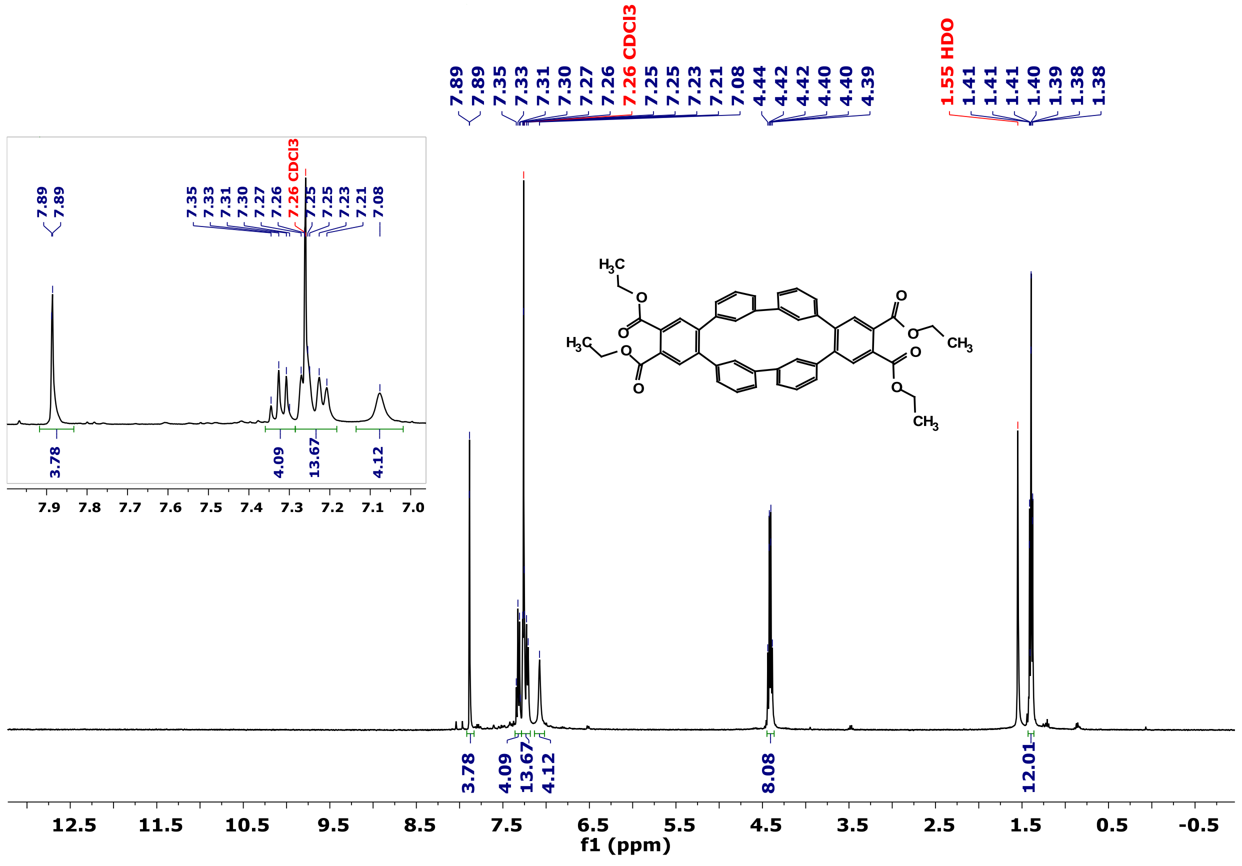

*Figure S18. ^{1}H NMR (400MHz, $CDCl_3$) spectrum of **TP[2]COOEt**.*

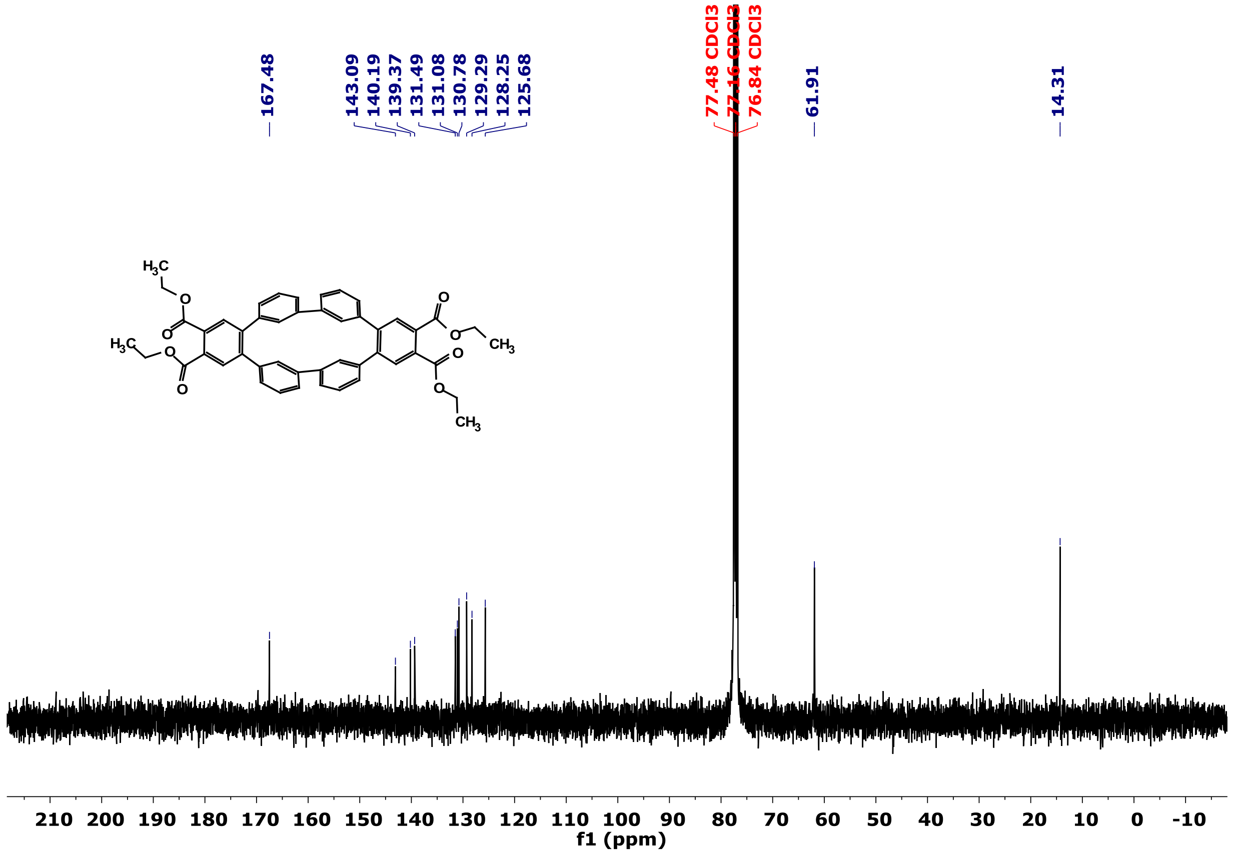

Figure S19. ^13^C NMR (400MHz, $CDCl_3$) spectrum of ***TP[2]COOEt****.*

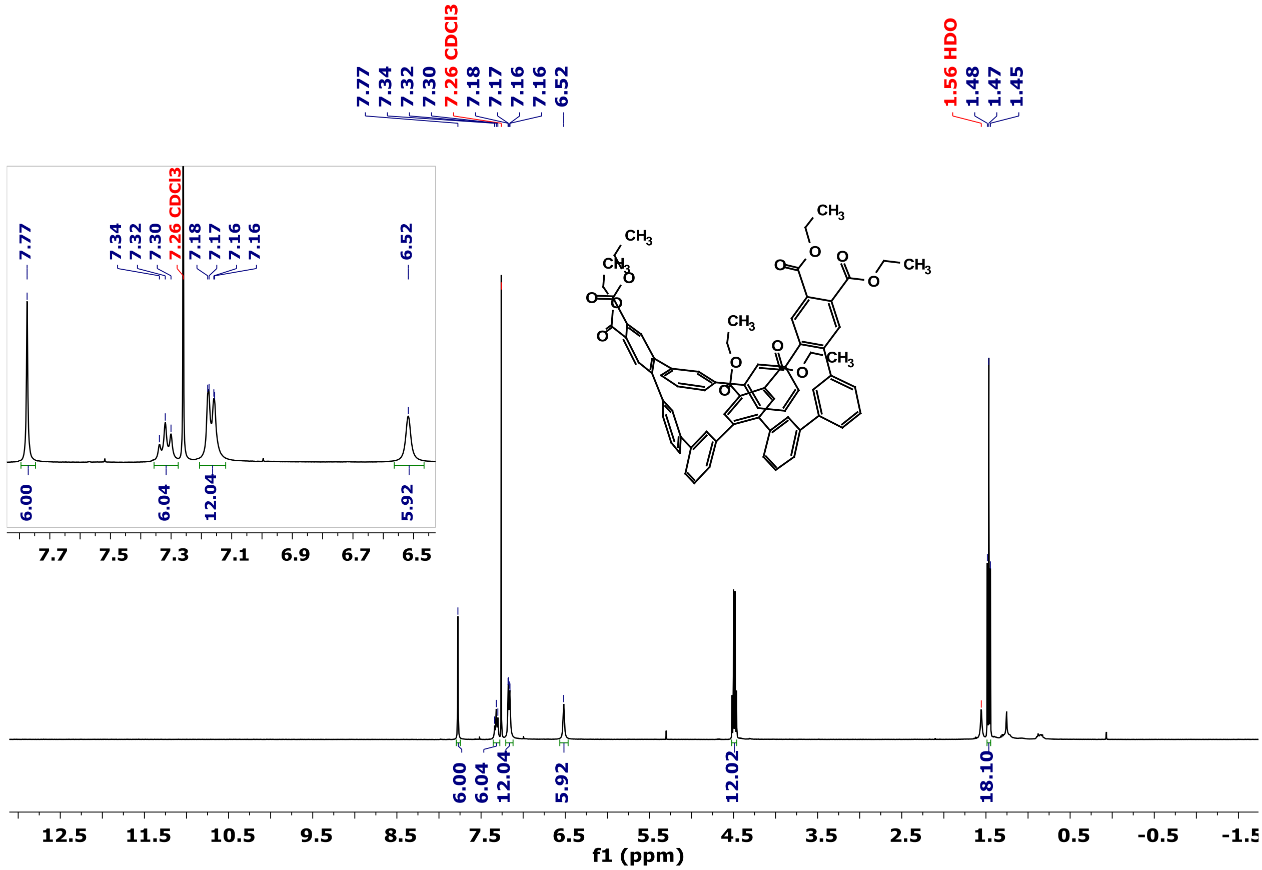

Figure S20. ^1^H NMR (400MHz, $CDCl_3$) spectrum of ***TP[3]COOEt****.*

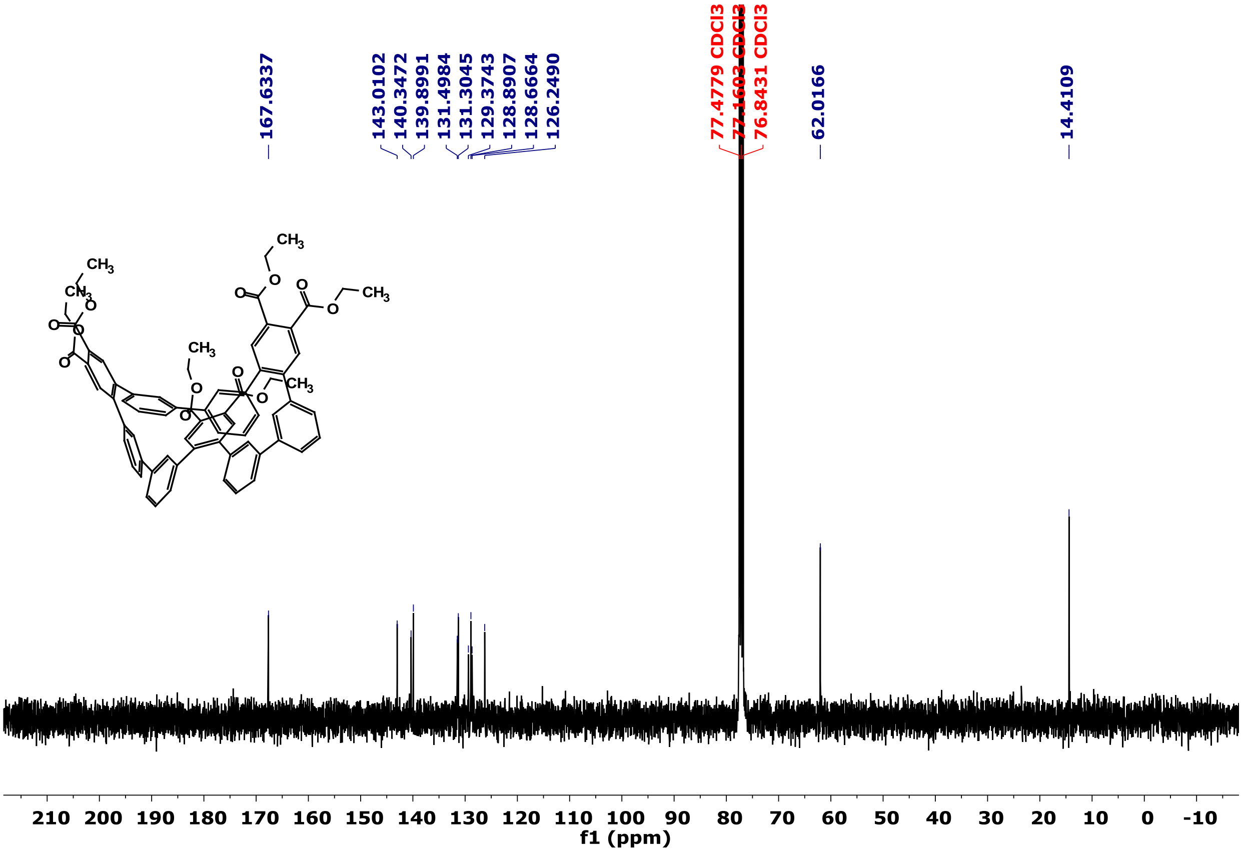

Figure S21. ^{13}C *NMR (400MHz,* $CDCl_3$*) spectrum of* ***TP[3]COOEt****.*

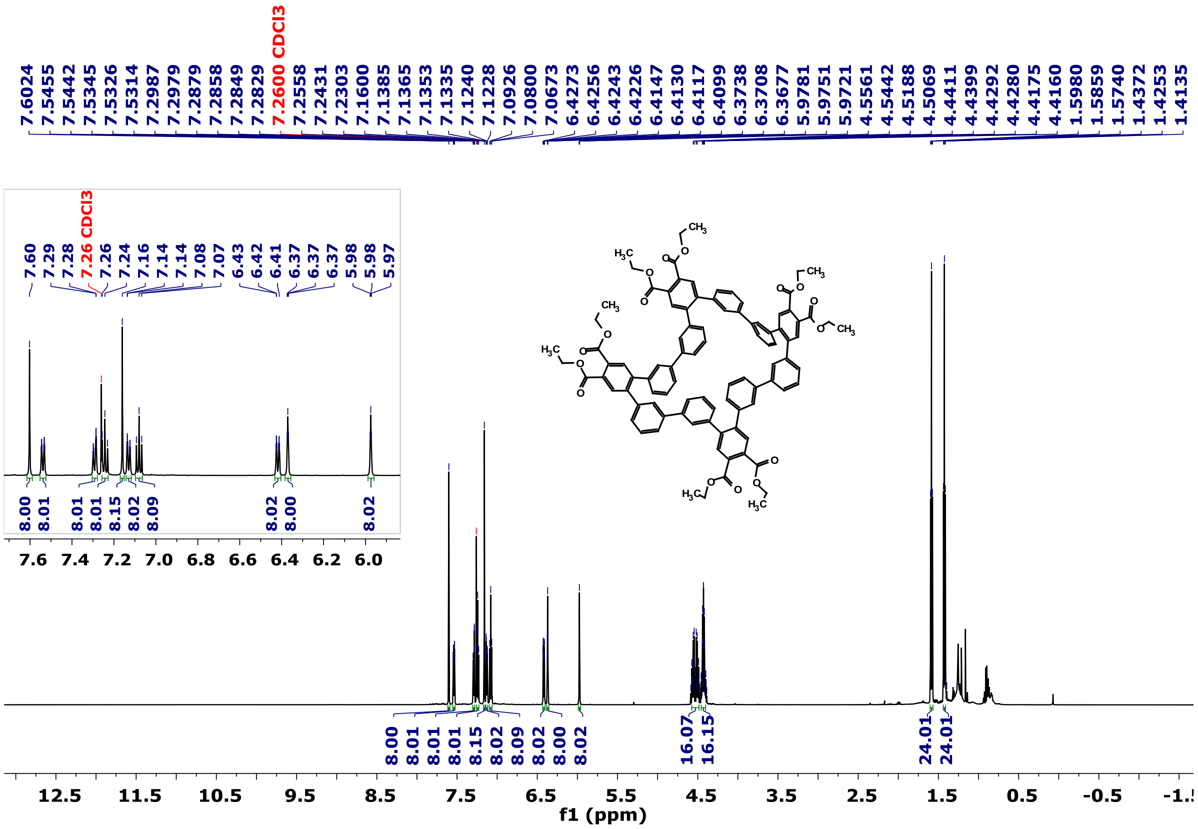

Figure S22. ^{1}H *NMR (601MHz,* $CDCl_3$*) spectrum of* ***TP[4]COOEt****.*

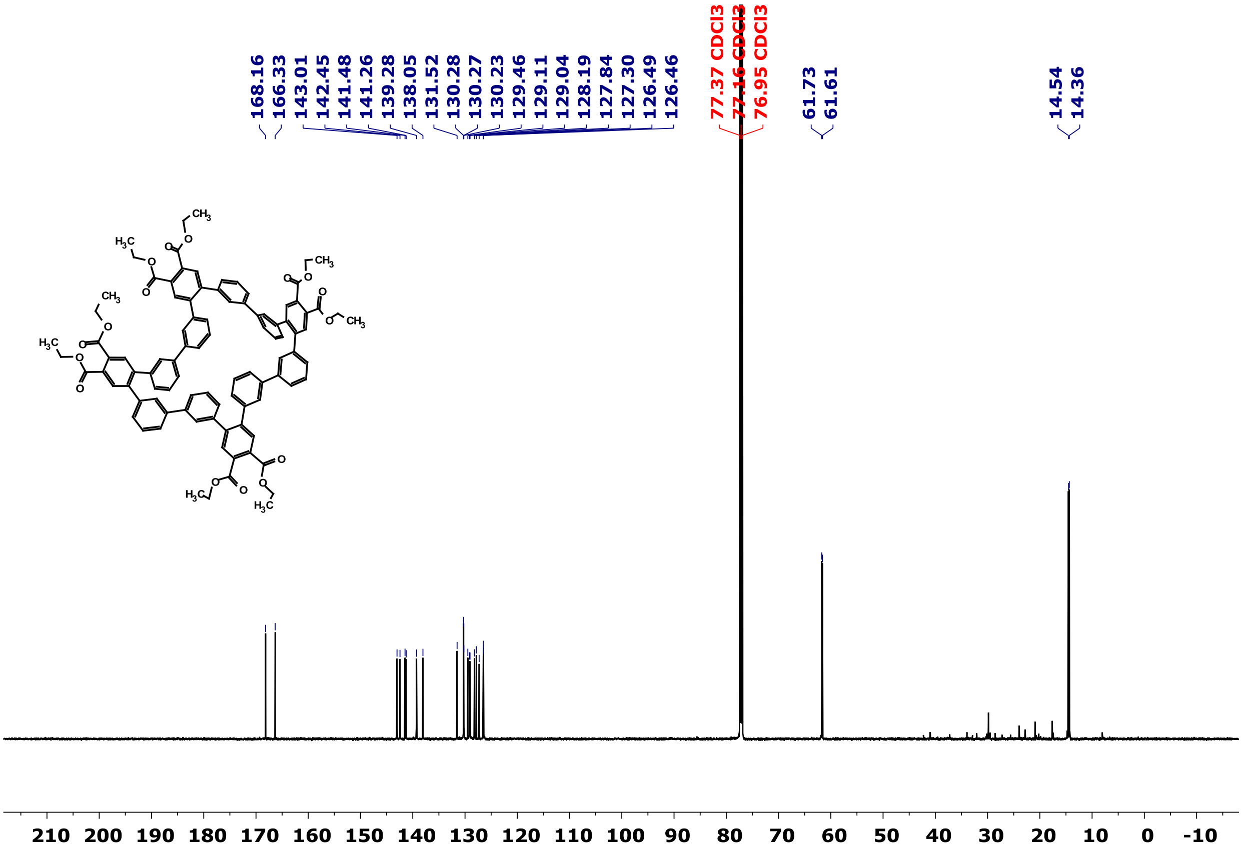

*Figure S23. ^{13}C NMR (601MHz, $CDCl_3$) spectrum of **TP[4]COOEt**.*

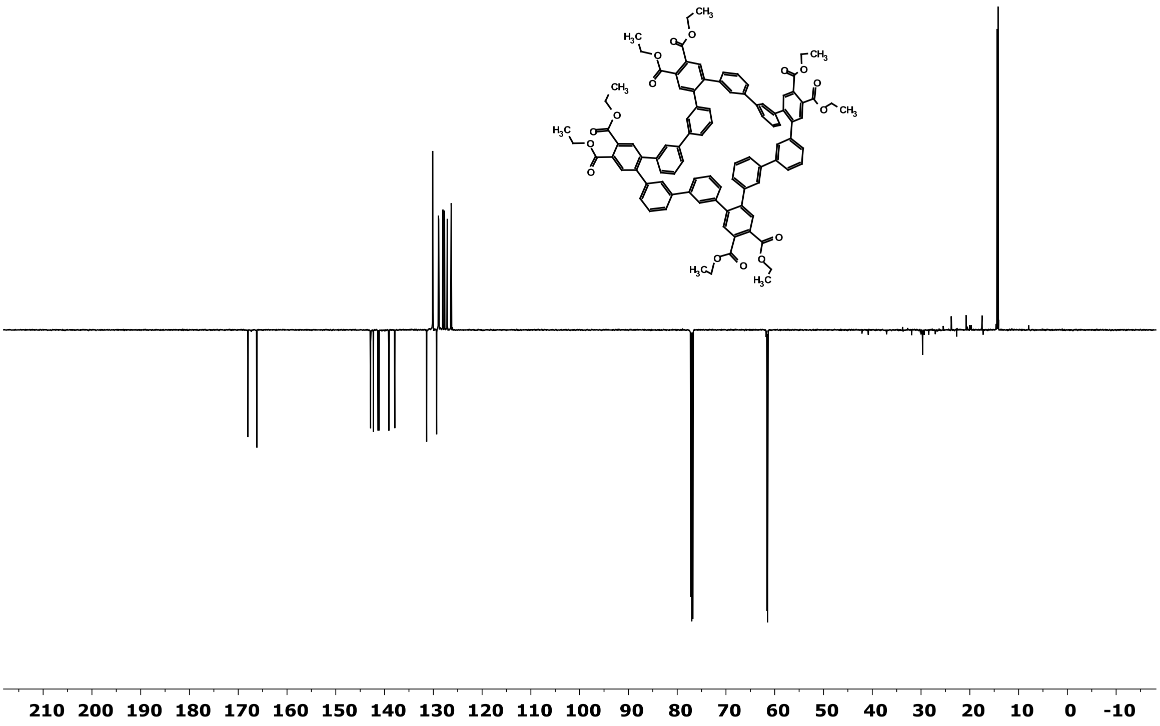

*Figure S24. ^{13}C NMR DEPT (151MHz, $CDCl_3$) spectrum of **TP[4]COOEt**.*

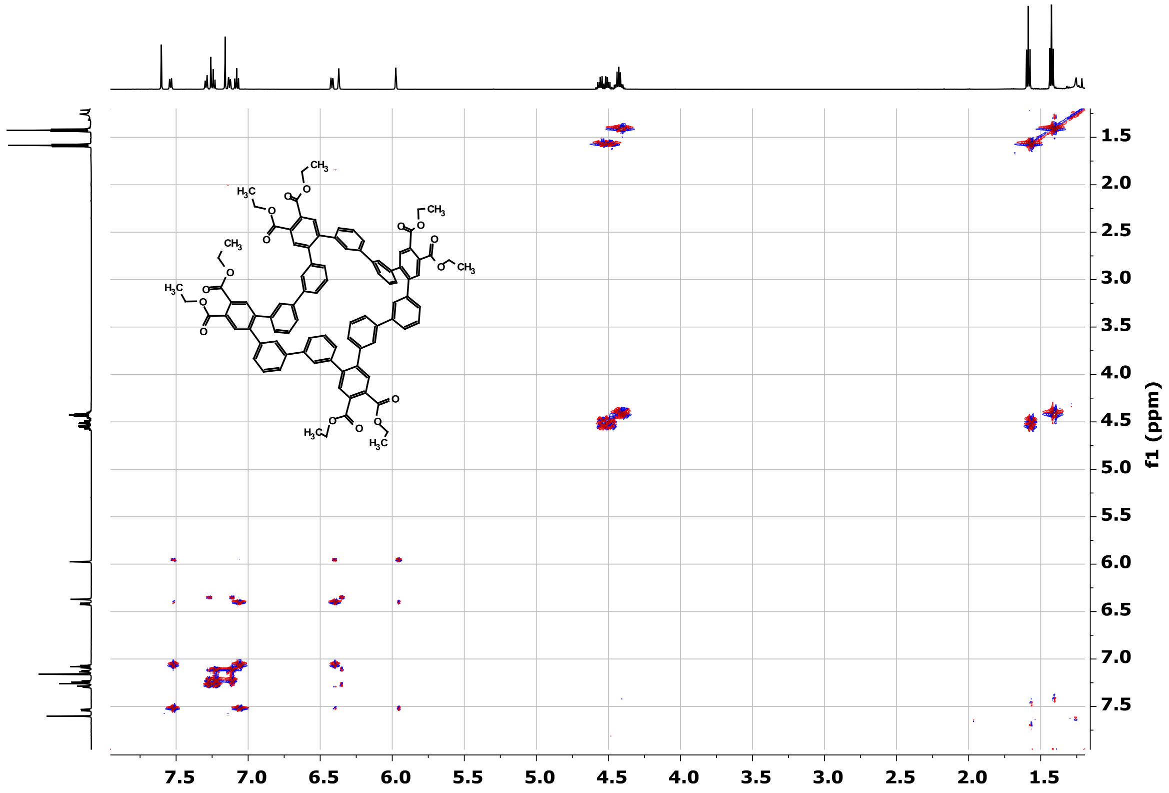

*Figure S25. ^{1}H-^{1}H-COSY (601MHz, $CDCl_3$) spectrum of **TP[4]COOEt**.*

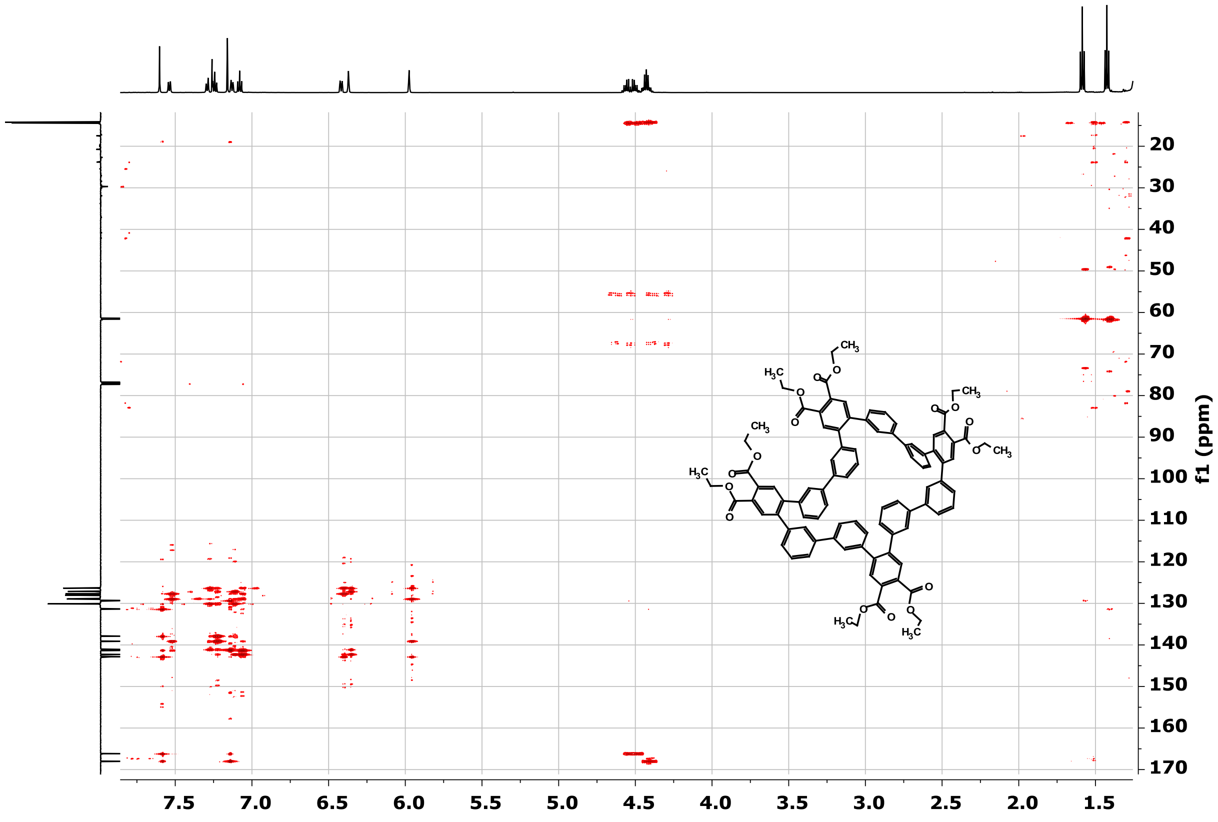

*Figure S26. ^{1}H-^{13}C-HSQC (601MHz-151MHz, $CDCl_3$) spectrum of **TP[4]COOEt**.*

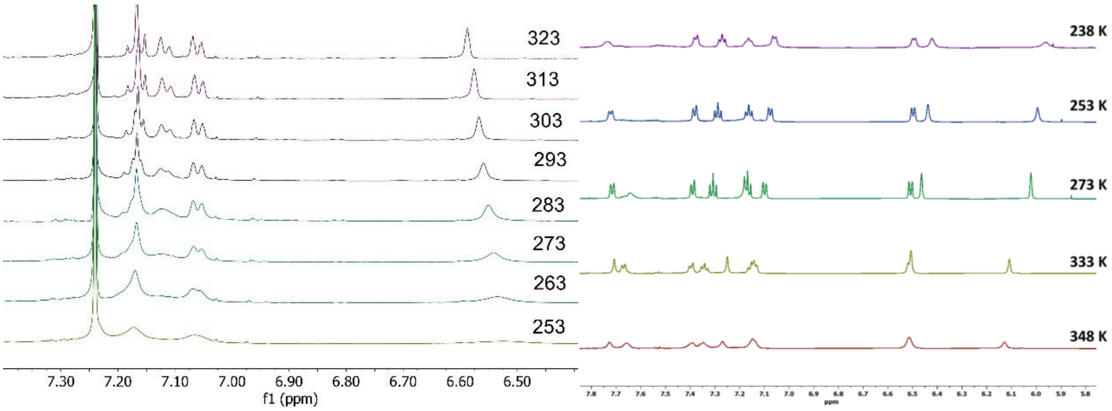

Figure S27. Templerature-dependent ^{1}H NMR (DMF-d_7) spectra of TP[3]CH_3 (left)and TP[4]COOEt (right).

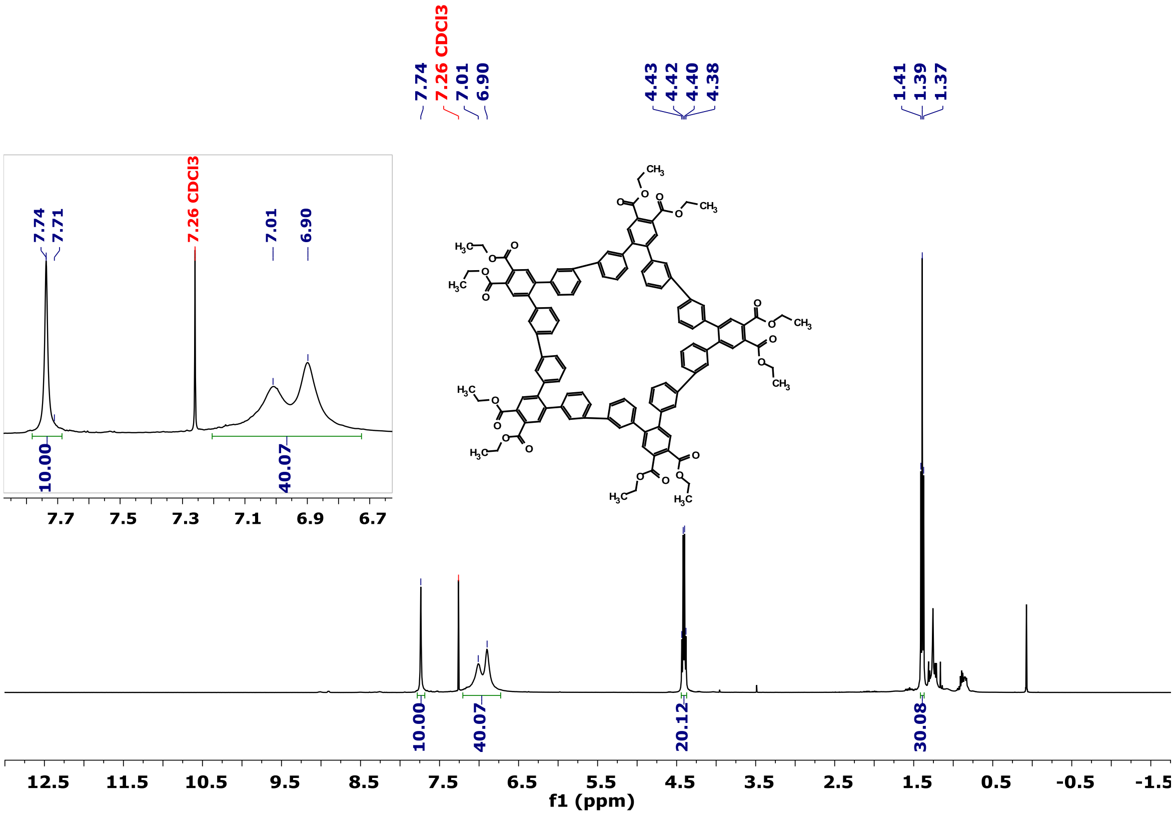

*Figure S28. ^{1}H NMR (400MHz, $CDCl_3$) spectrum of **TP[5]COOEt**.*

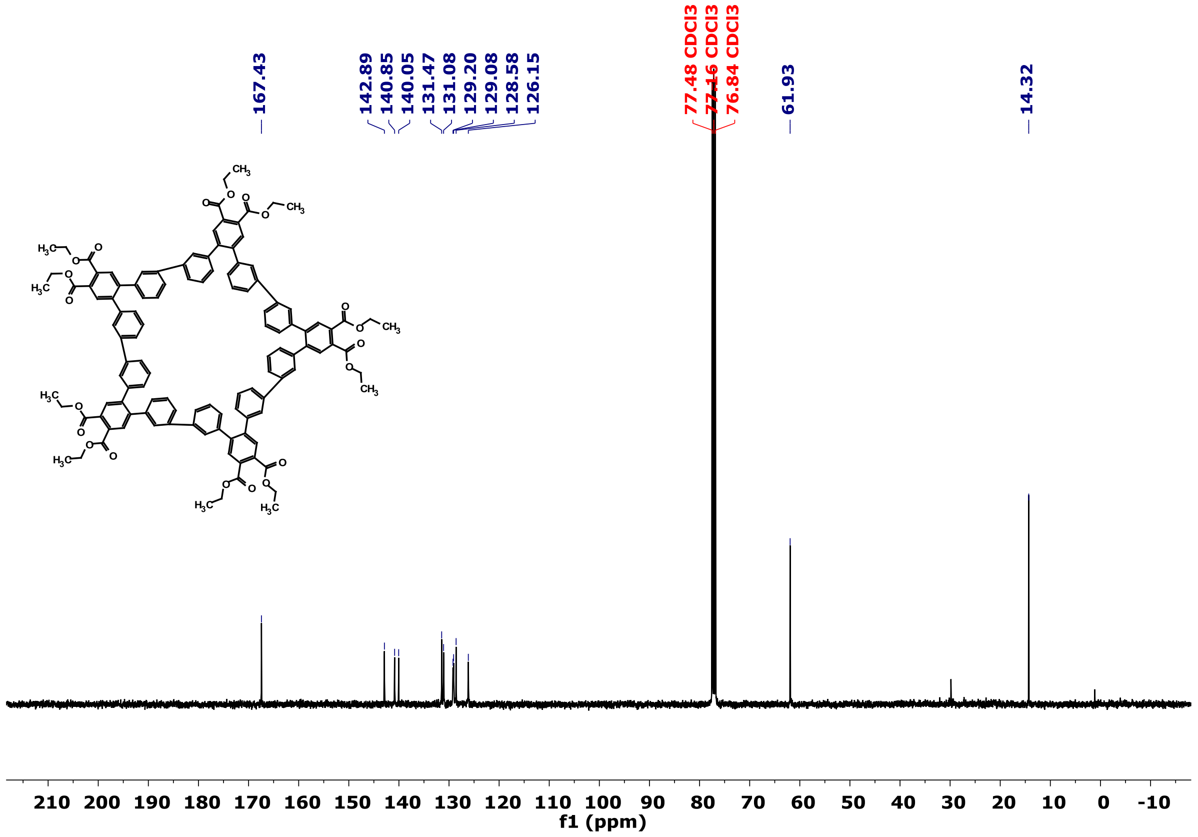

*Figure S29. ^{13}C NMR (101MHz, $CDCl_3$) spectrum of **TP[5]COOEt**.*

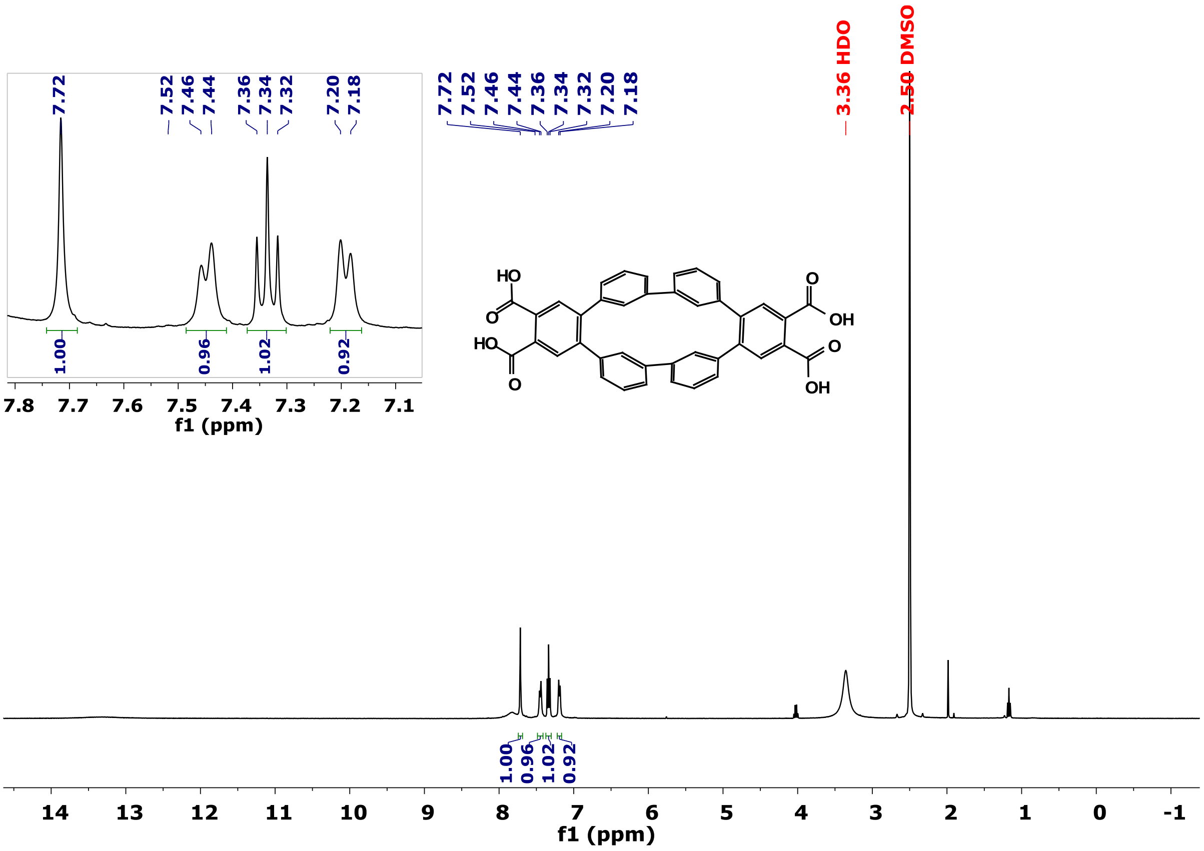

*Figure S30. ^{1}H NMR (400MHz, DMSO-d6) spectrum of **TP[2]COOH**.*

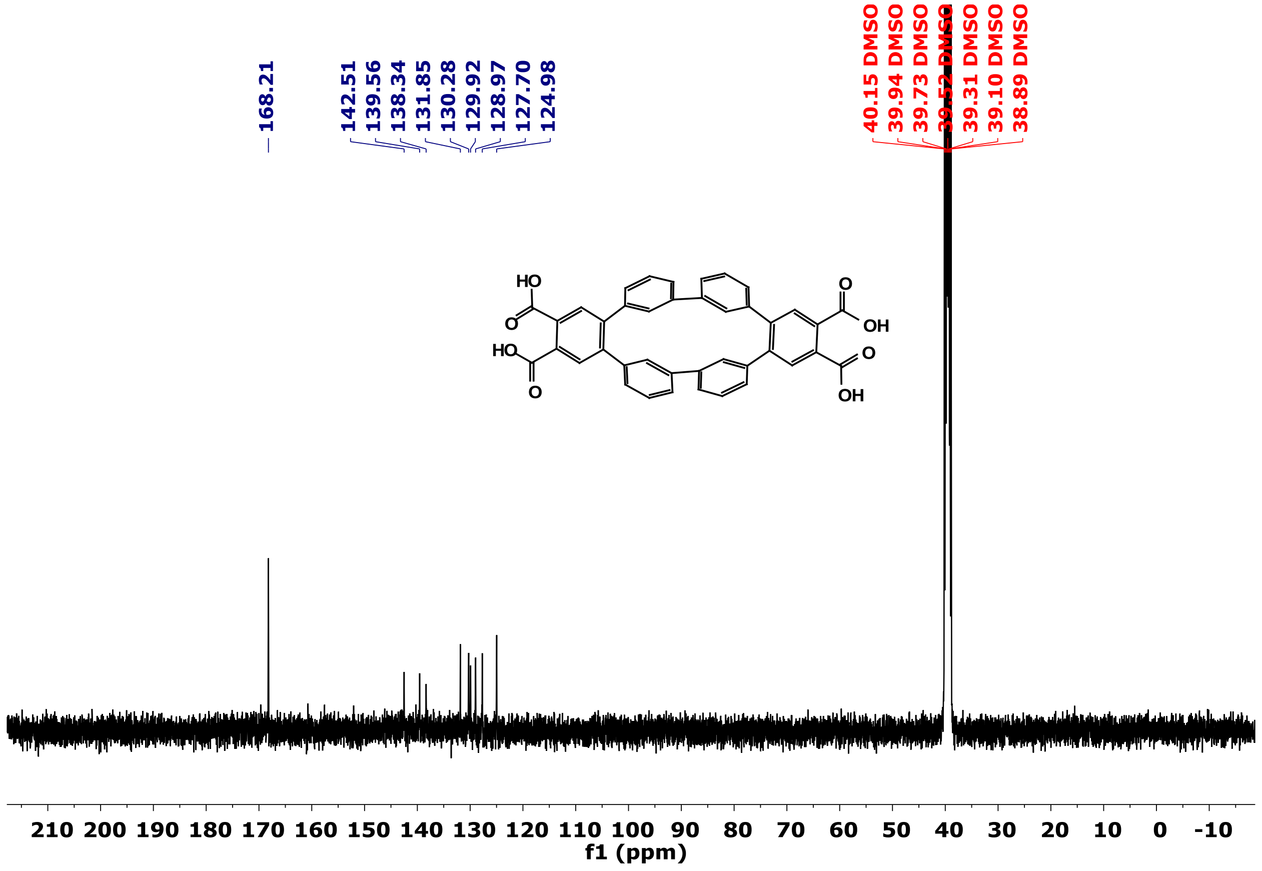

Figure S31. ^{13}C *NMR (101MHz, DMSO-d6) spectrum of* ***TP[2]COOH***.

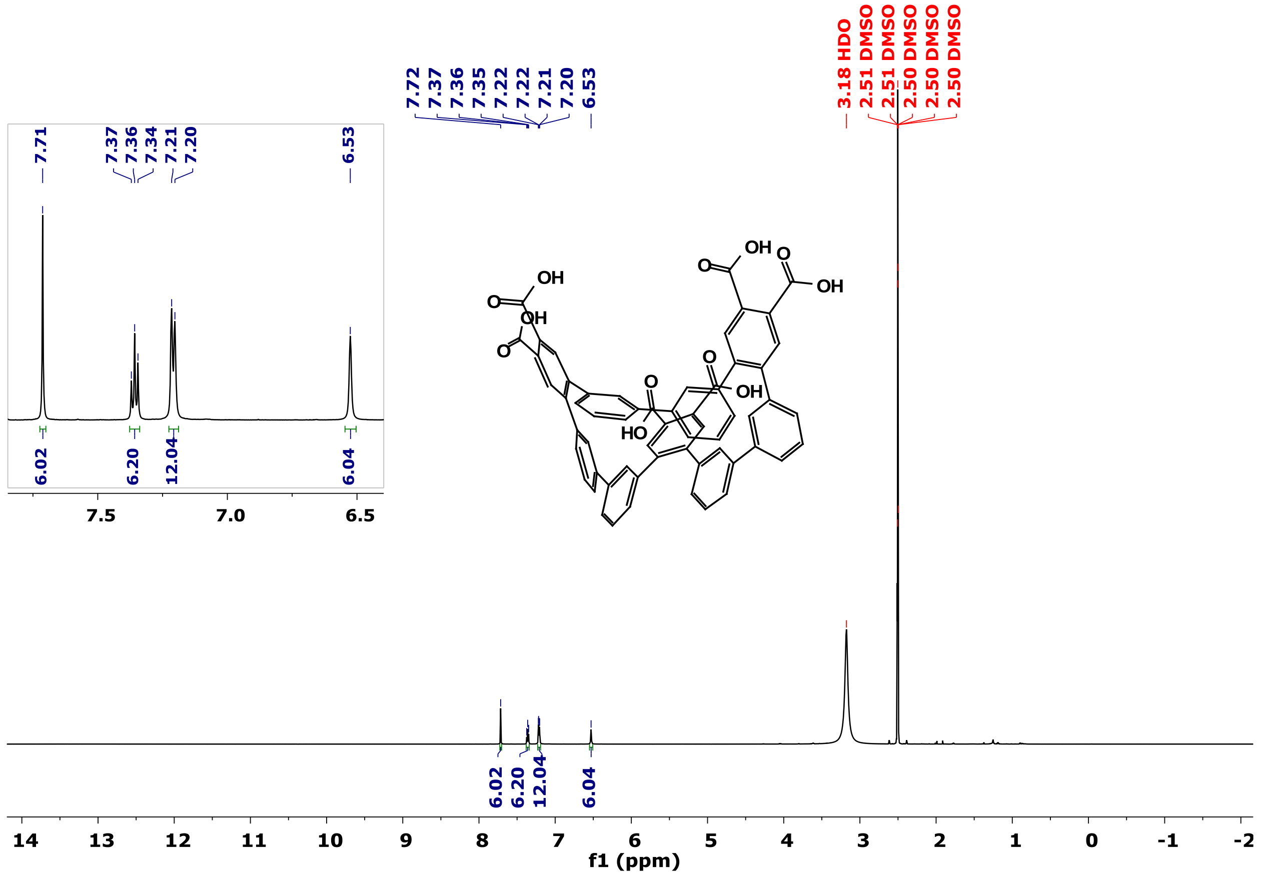

Figure S32. ^{1}H *NMR (601MHz, DMSO-d6) spectrum of* ***TP[3]COOH***.

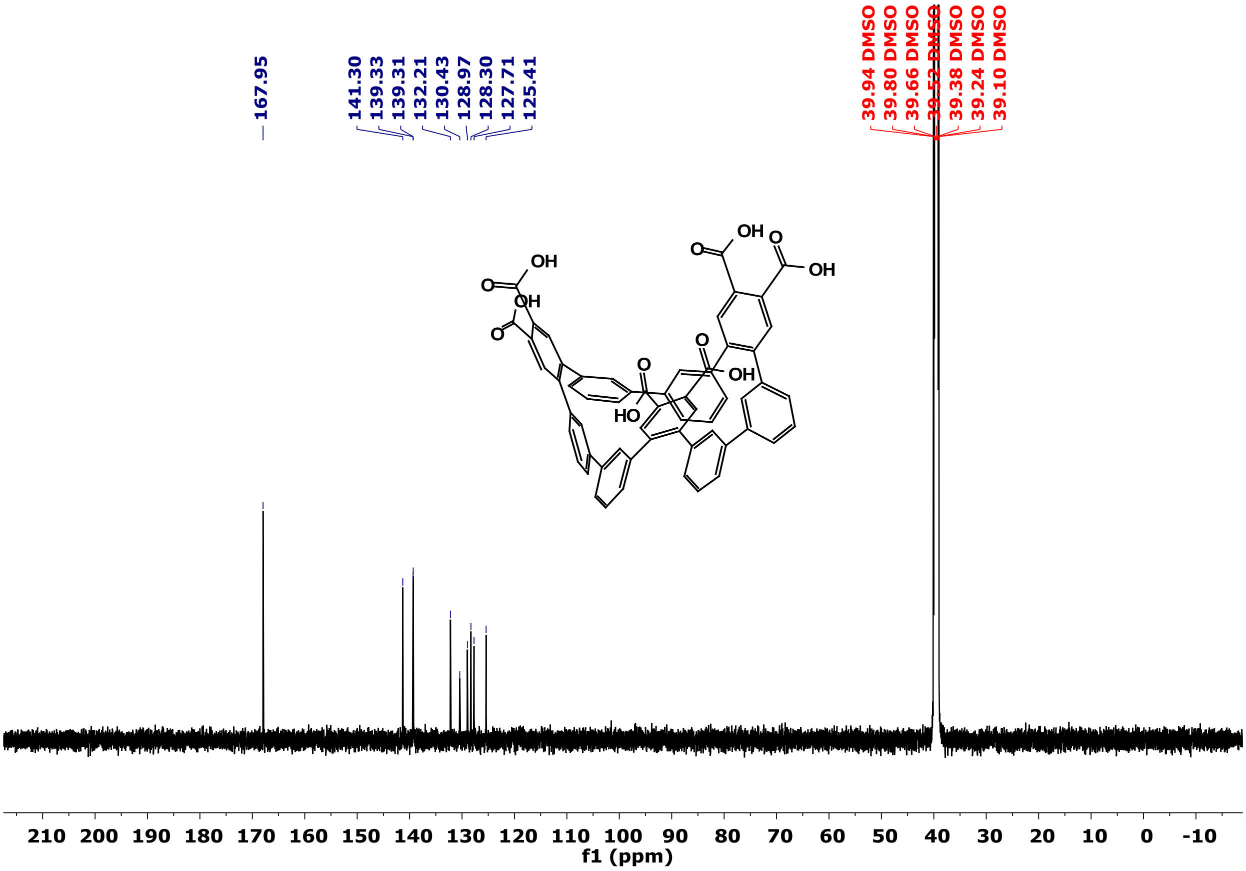

Figure S33. ^{13}C NMR (151MHz, DMSO-d6) spectrum of ***TP[3]COOH****.*

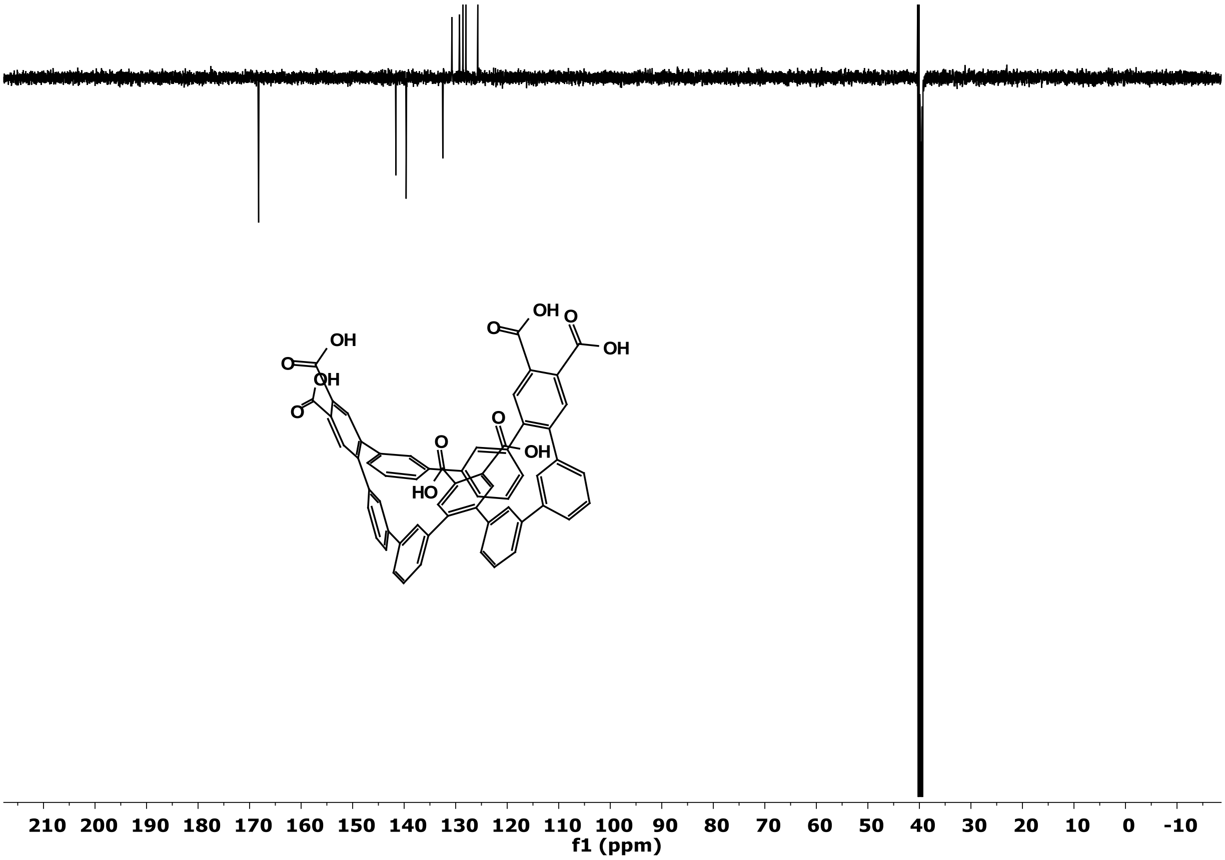

Figure S34. ^{13}C NMR DEPT (151MHz, DMSO-d6) spectrum of ***TP[3]COOH****.*

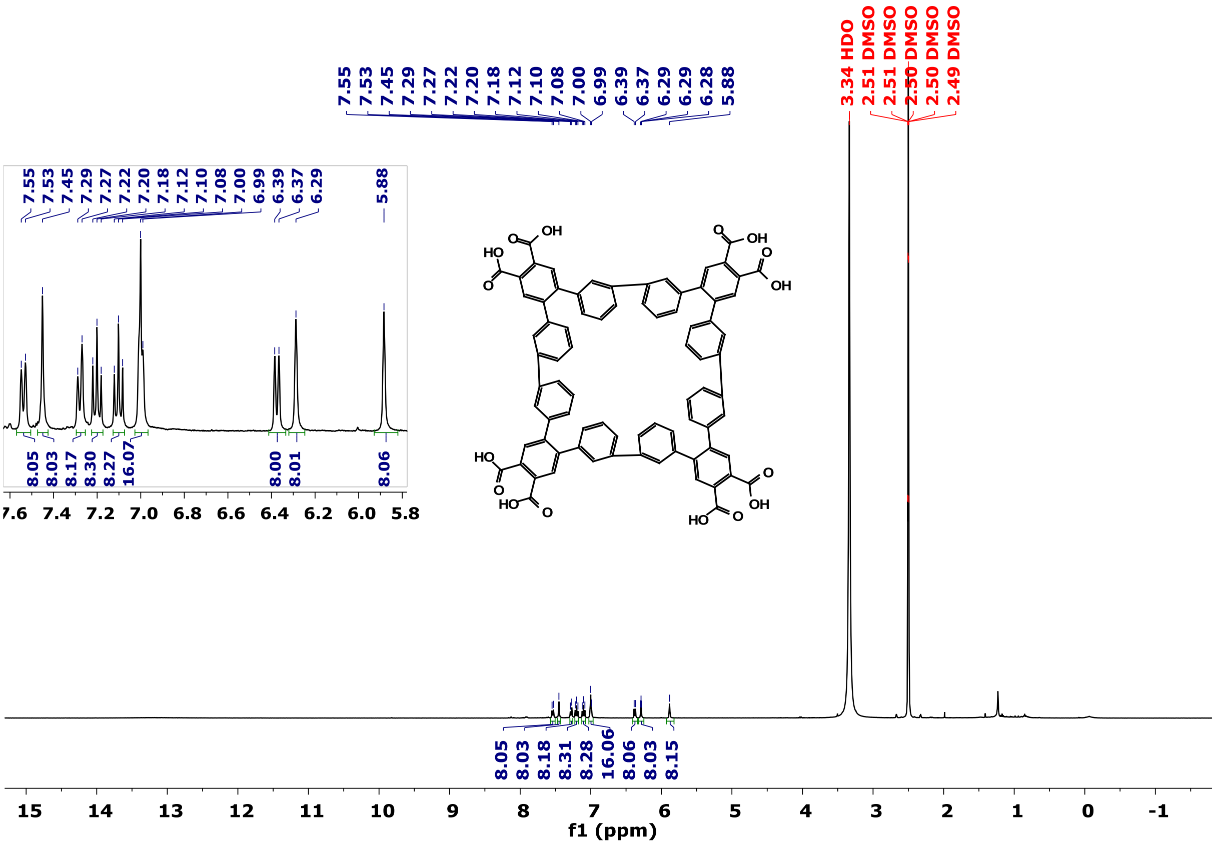

Figure S35. ^{1}H NMR (601MHz, DMSO-d6) spectrum of **TP[4]COOH**.

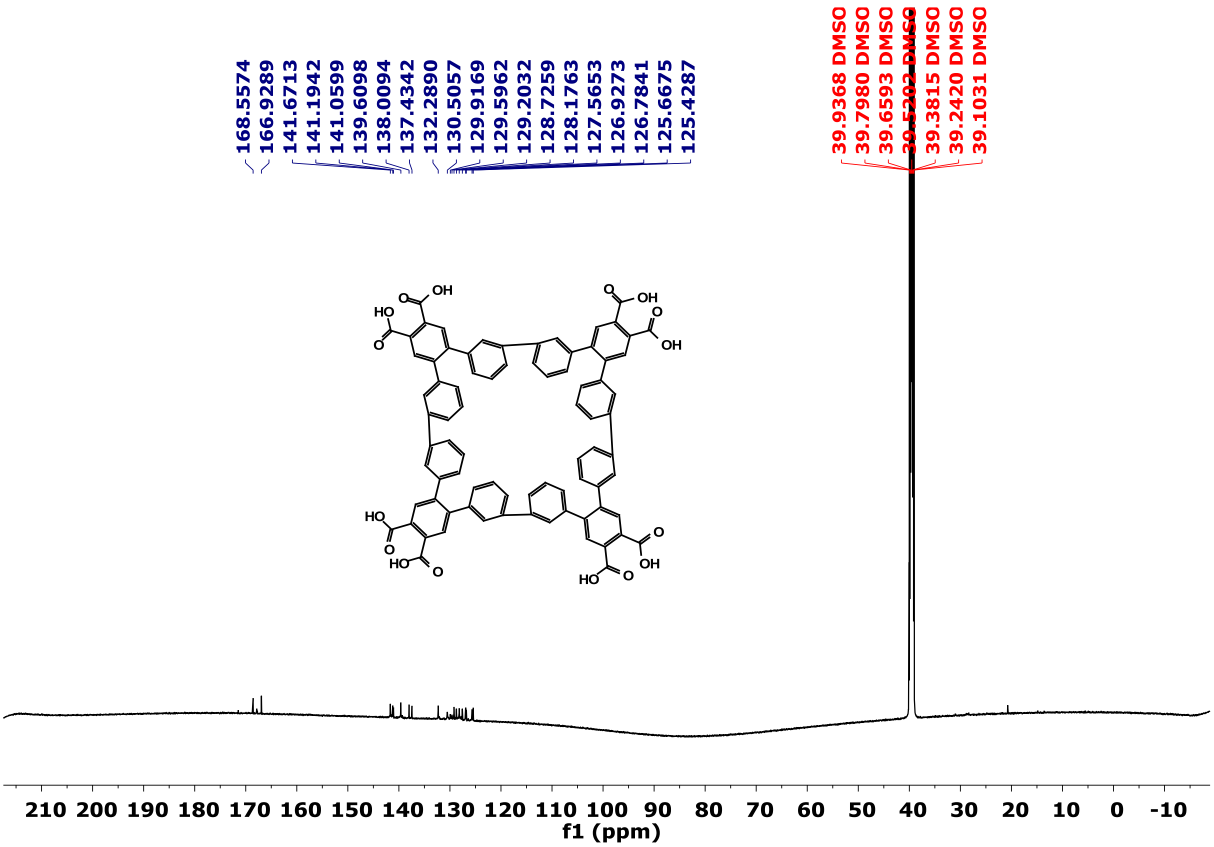

Figure S36. ^{13}C NMR (151MHz, DMSO-d6) spectrum of **TP[4]COOH**.

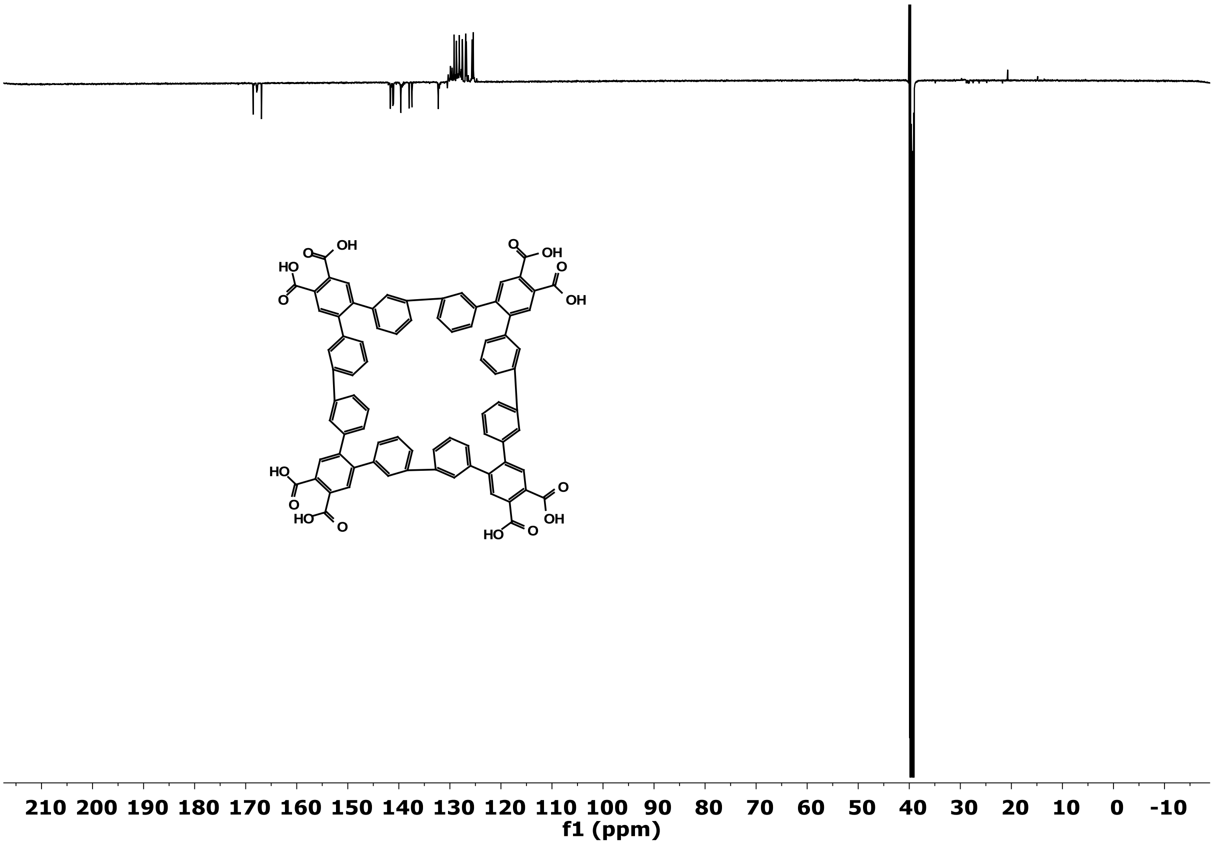

Figure S37. ^{13}*C NMR DEPT (151MHz, DMSO-d6) spectrum of* ***TP[4]COOH***.

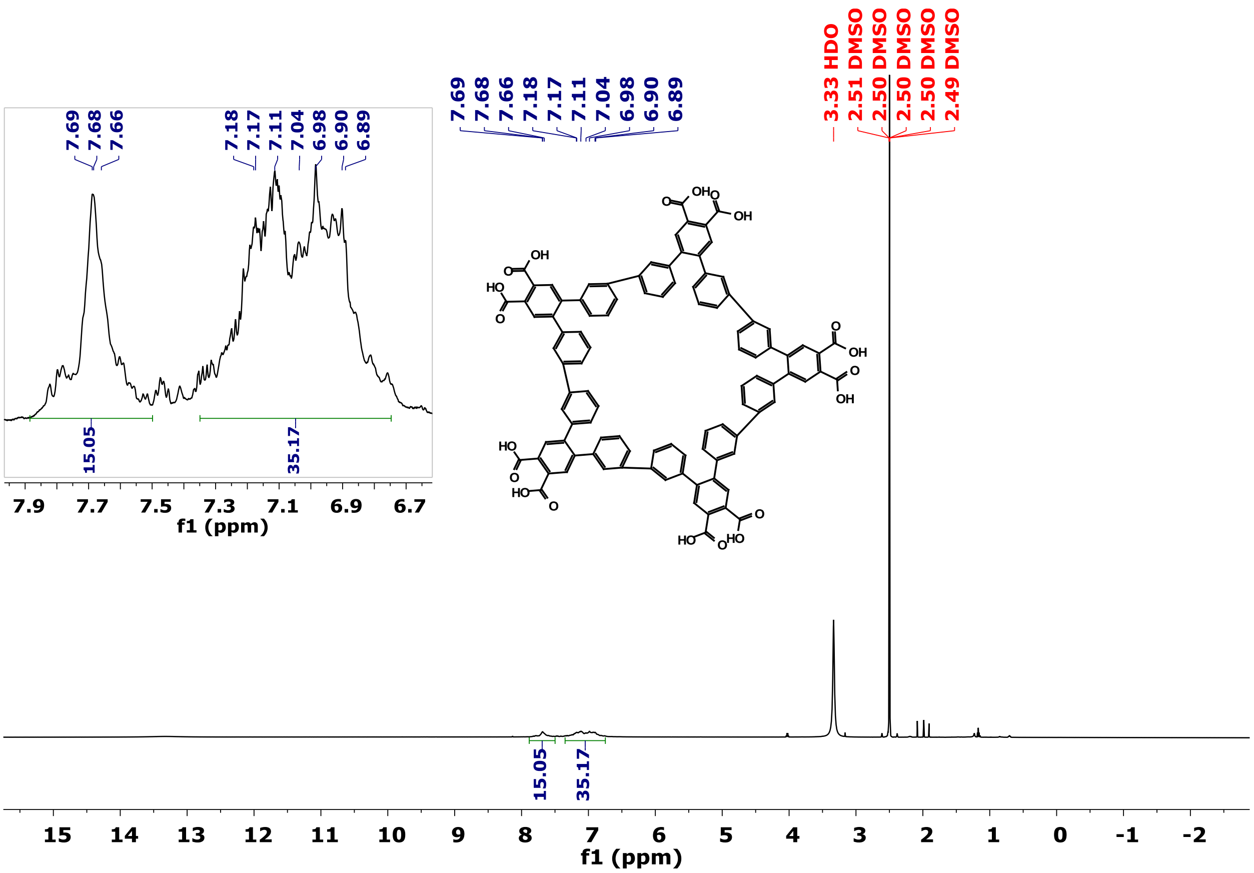

Figure S38. ^{1}*H NMR (601MHz, DMSO-d6) spectrum of* ***TP[5]COOH***.

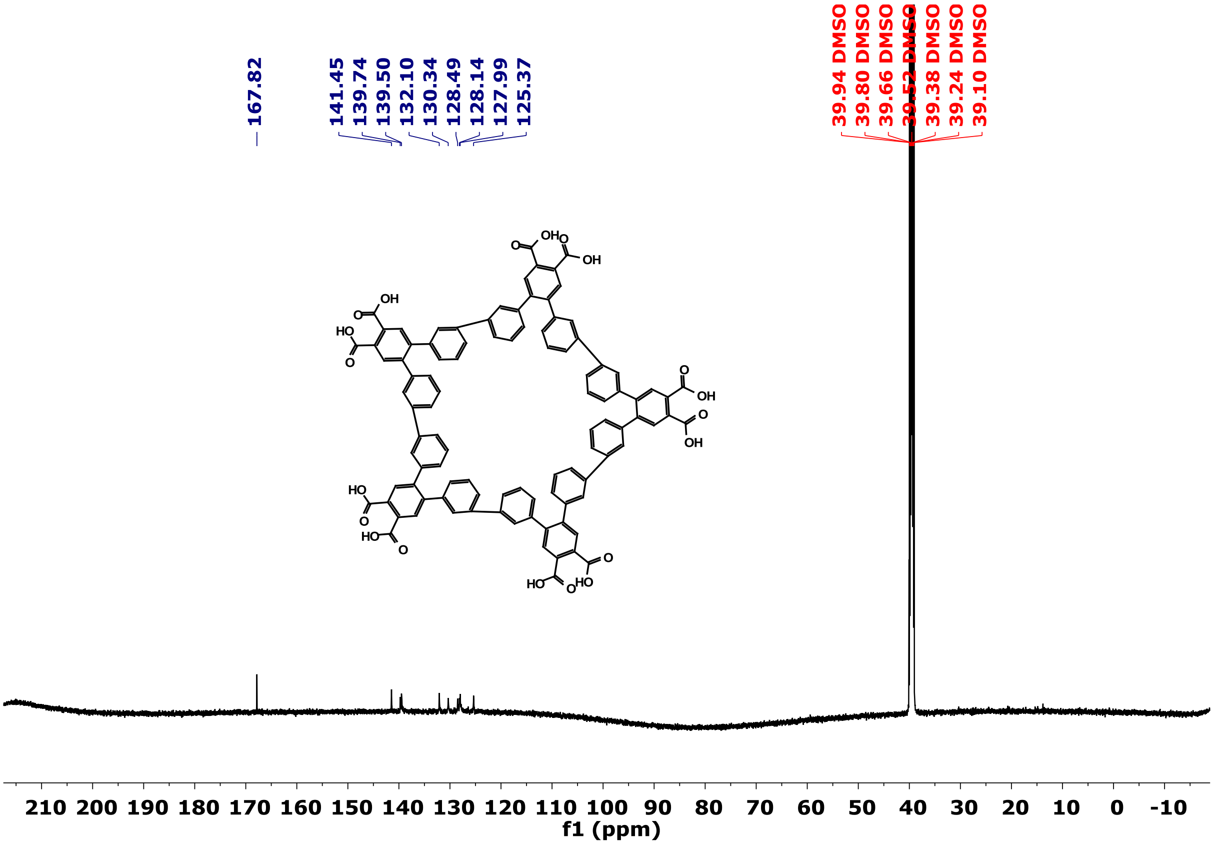

Figure S39. ^{13}C NMR DEPT (151MHz, DMSO-d6) spectrum of ***TP[5]COOH****.*

4. Mass Spectra

Acquisition Parameter

Source Type	APPI	Ion Polarity	Positive	Set Nebulizer	5.2 Bar
Focus	Not active	Set Capillary	700 V	Set Dry Heater	220 °C
Scan Begin	80 m/z	Set End Plate Offset	-500 V	Set Dry Gas	1.2 l/min
Scan End	1550 m/z	Set Charging Voltage	0 V	Set Divert Valve	Waste
		Set Corona	0 nA	Set APCI Heater	250 °C

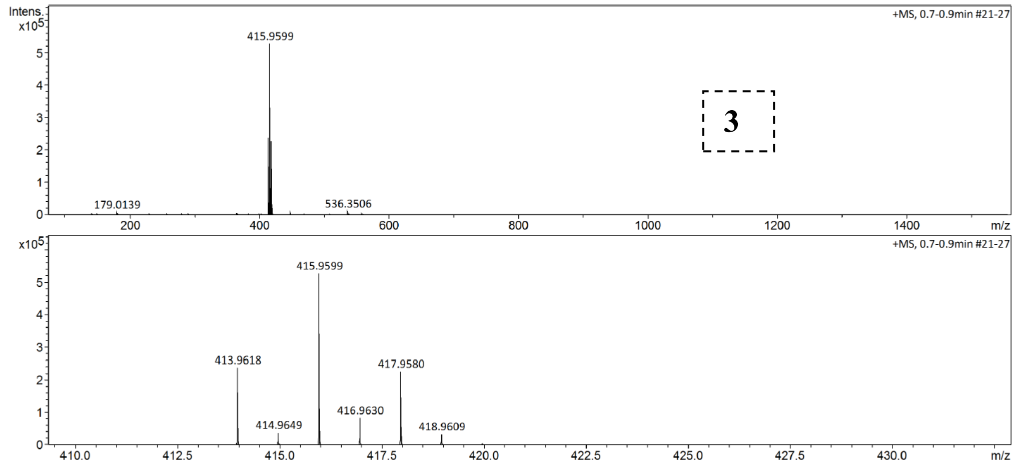

Acquisition Parameter

Source Type	APPI	Ion Polarity	Positive	Set Nebulizer	5.2 Bar
Focus	Not active	Set Capillary	700 V	Set Dry Heater	220 °C
Scan Begin	80 m/z	Set End Plate Offset	-500 V	Set Dry Gas	1.2 l/min
Scan End	1550 m/z	Set Charging Voltage	0 V	Set Divert Valve	Waste
		Set Corona	0 nA	Set APCI Heater	250 °C

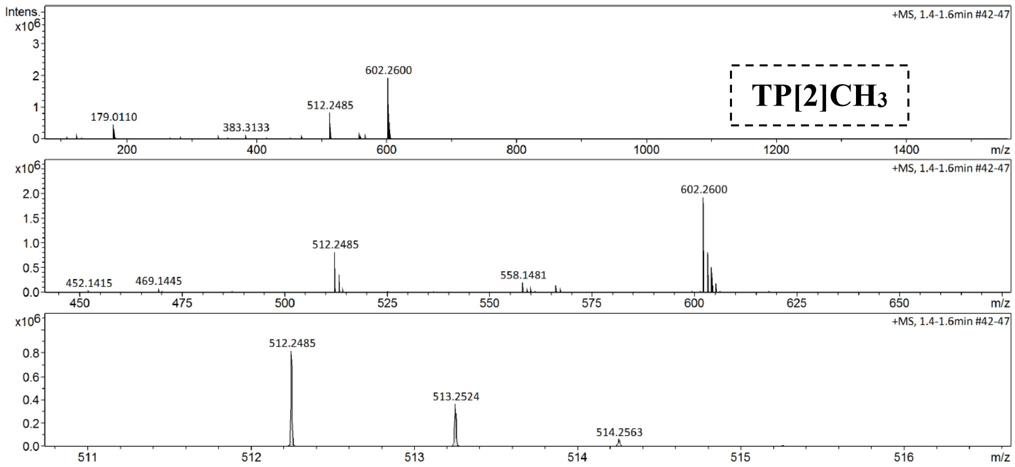

Acquisition Parameter

Source Type	APPI	Ion Polarity	Positive	Set Nebulizer	5.2 Bar
Focus	Not active	Set Capillary	700 V	Set Dry Heater	220 °C
Scan Begin	80 m/z	Set End Plate Offset	-500 V	Set Dry Gas	1.2 l/min
Scan End	1550 m/z	Set Charging Voltage	0 V	Set Divert Valve	Waste
		Set Corona	0 nA	Set APCI Heater	300 °C

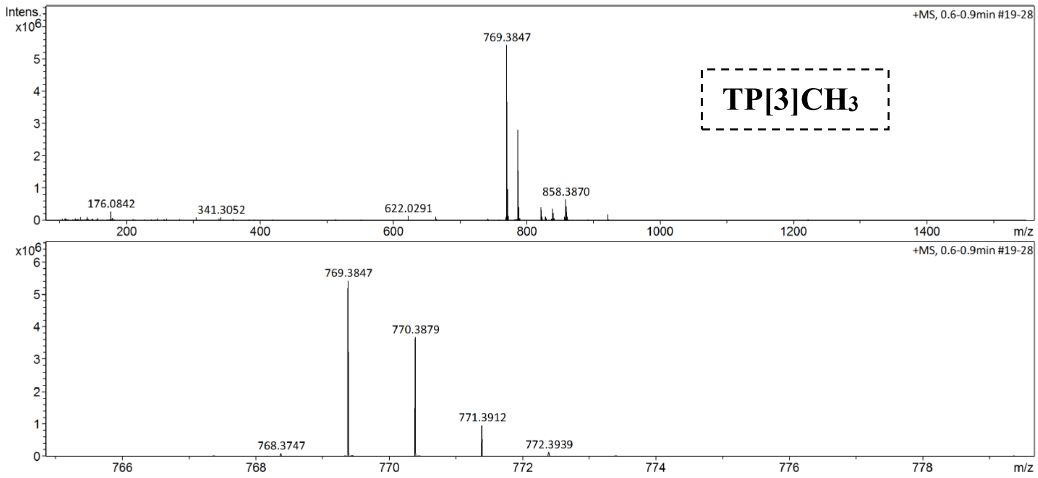

Acquisition Parameter

Source Type	APPI	Ion Polarity	Positive	Set Nebulizer	5.2 Bar
Focus	Not active	Set Capillary	700 V	Set Dry Heater	220 °C
Scan Begin	80 m/z	Set End Plate Offset	-500 V	Set Dry Gas	1.2 l/min
Scan End	1550 m/z	Set Charging Voltage	0 V	Set Divert Valve	Waste
		Set Corona	0 nA	Set APCI Heater	250 °C

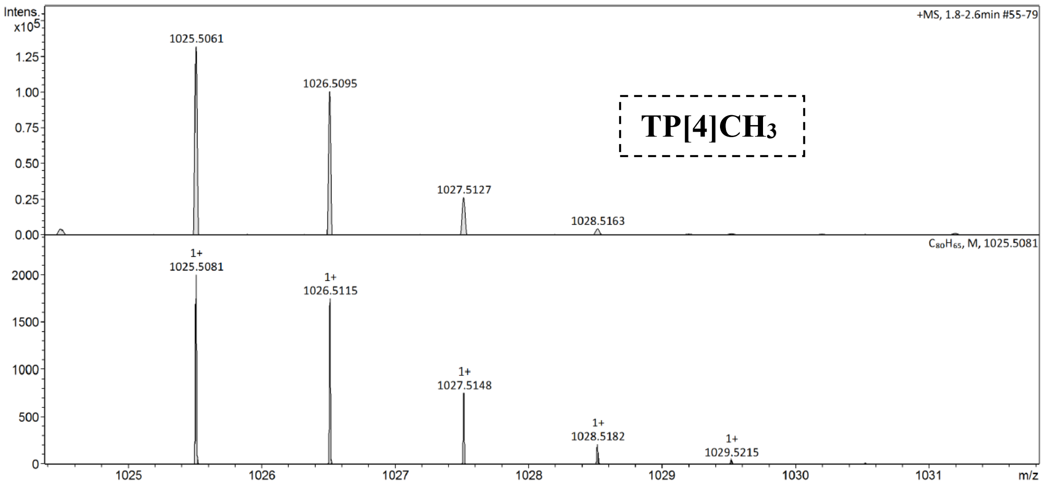

Acquisition Parameter

Source Type	APPI	Ion Polarity	Positive	Set Nebulizer	5.2 Bar
Focus	Not active	Set Capillary	700 V	Set Dry Heater	220 °C
Scan Begin	80 m/z	Set End Plate Offset	-500 V	Set Dry Gas	1.2 l/min
Scan End	1550 m/z	Set Charging Voltage	0 V	Set Divert Valve	Waste
		Set Corona	0 nA	Set APCI Heater	250 °C

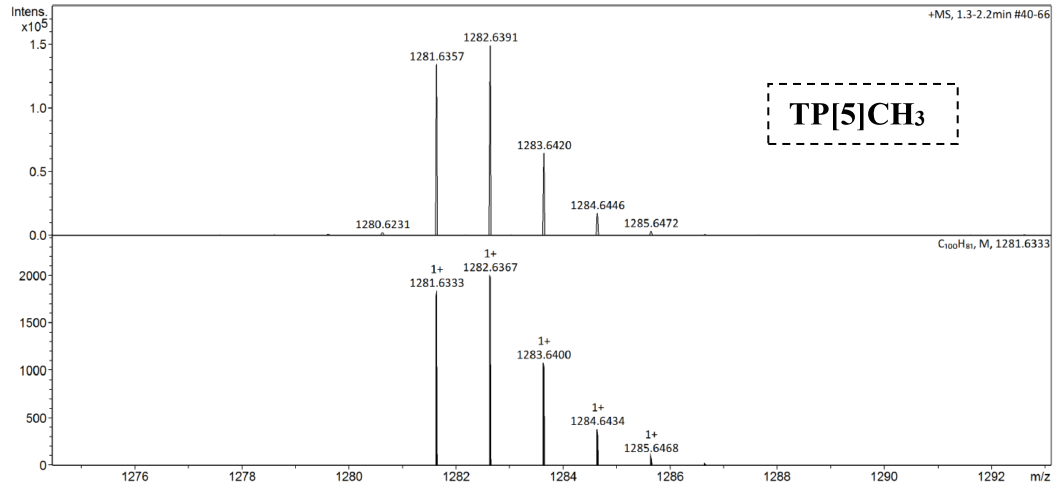

Acquisition Parameter

Source Type	APPI	Ion Polarity	Positive	Set Nebulizer	2.5 Bar
Focus	Not active	Set Capillary	750 V	Set Dry Heater	200 °C
Scan Begin	300 m/z	Set End Plate Offset	-500 V	Set Dry Gas	1.5 l/min
Scan End	2900 m/z	Set Charging Voltage	0 V	Set Divert Valve	Waste
		Set Corona	0 nA	Set APCI Heater	250 °C

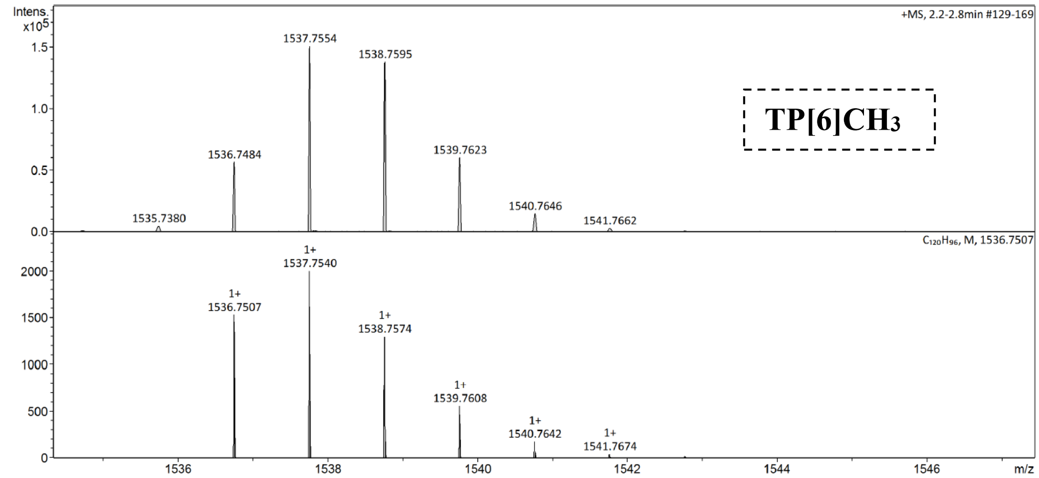

Acquisition Parameter

Source Type	APPI	Ion Polarity	Positive	Set Nebulizer	2.5 Bar
Focus	Not active	Set Capillary	750 V	Set Dry Heater	200 °C
Scan Begin	300 m/z	Set End Plate Offset	-500 V	Set Dry Gas	1.5 l/min
Scan End	2900 m/z	Set Charging Voltage	0 V	Set Divert Valve	Waste
		Set Corona	0 nA	Set APCI Heater	350 °C

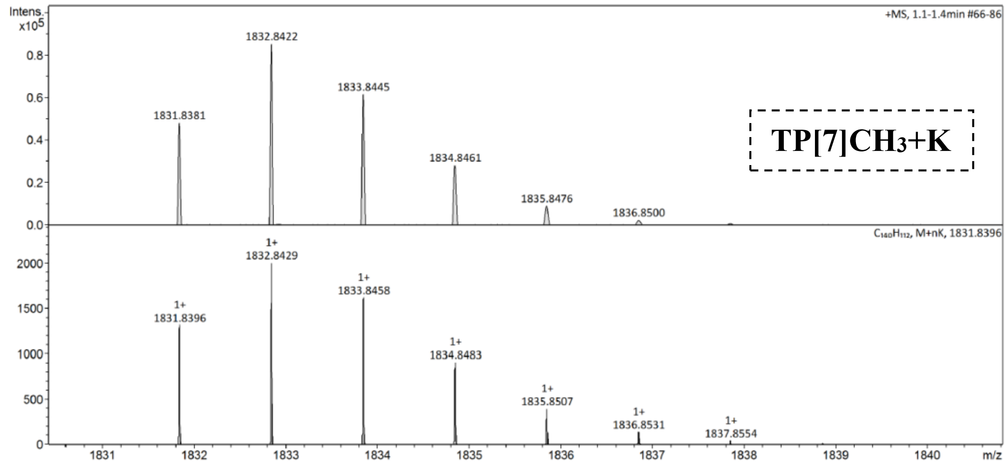

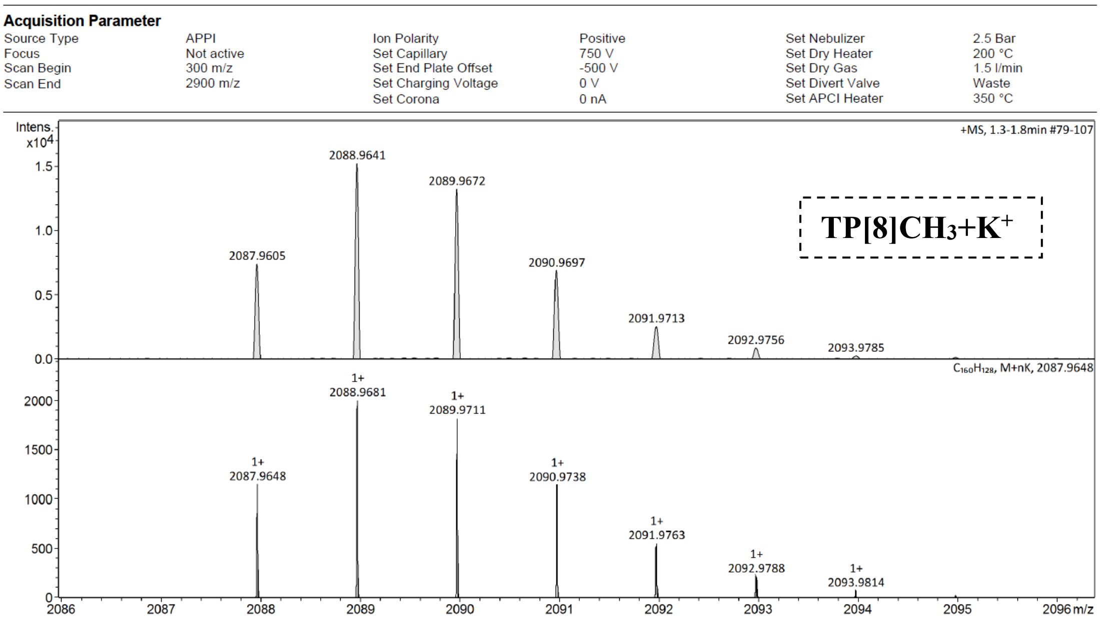
Acquisition Parameter
Source Type APPI
Focus Not active
Scan Begin 300 m/z
Scan End 2900 m/z
Ion Polarity Positive
Set Capillary 750 V
Set End Plate Offset -500 V
Set Charging Voltage 0 V
Set Corona 0 nA
Set Nebulizer 2.5 Bar
Set Dry Heater 200 °C
Set Dry Gas 1.5 l/min
Set Divert Valve Waste
Set APCI Heater 350 °C
Intens. x10^4
+MS, 1.3-1.8min #79-107
TP[8]CH3+K+
2087.9605
2088.9641
2089.9672
2090.9697
2091.9713
2092.9756
2093.9785
C160H128, M+nK, 2087.9648
1+ 2087.9648
1+ 2088.9681
1+ 2089.9711
1+ 2090.9738
1+ 2091.9763
1+ 2092.9788
1+ 2093.9814
m/z

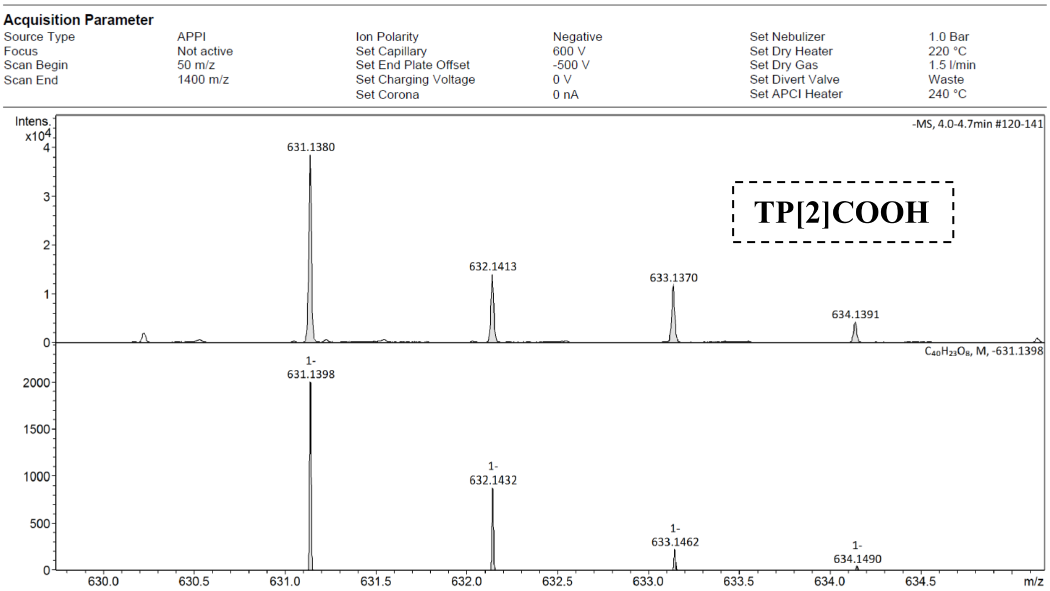
Acquisition Parameter
Source Type APPI
Focus Not active
Scan Begin 50 m/z
Scan End 1400 m/z
Ion Polarity Negative
Set Capillary 600 V
Set End Plate Offset -500 V
Set Charging Voltage 0 V
Set Corona 0 nA
Set Nebulizer 1.0 Bar
Set Dry Heater 220 °C
Set Dry Gas 1.5 l/min
Set Divert Valve Waste
Set APCI Heater 240 °C
Intens. x10^4
-MS, 4.0-4.7min #120-141
TP[2]COOH
631.1380
632.1413
633.1370
634.1391
C40H23O8, M, -631.1398
1- 631.1398
1- 632.1432
1- 633.1462
1- 634.1490
m/z

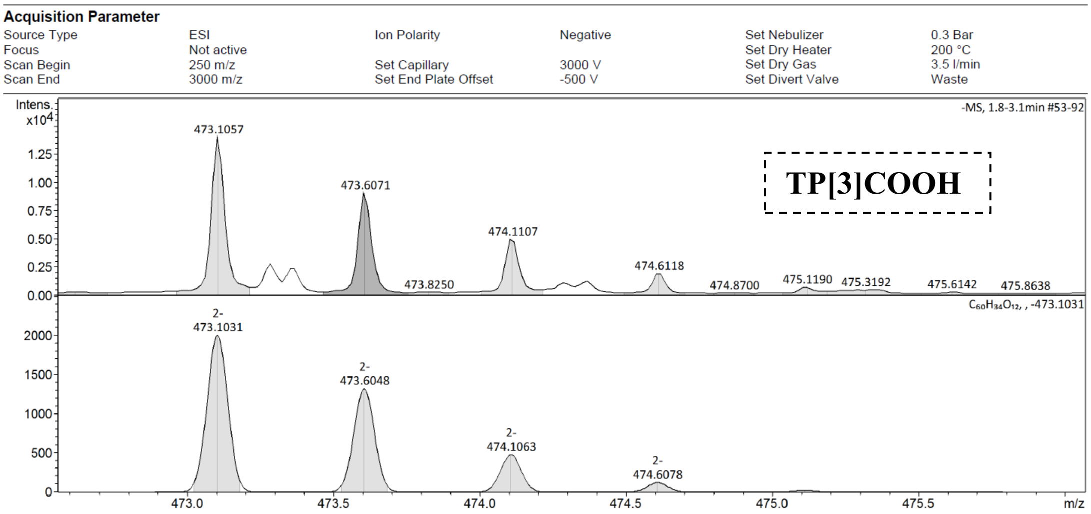
Acquisition Parameter
Source Type ESI
Focus Not active
Scan Begin 250 m/z
Scan End 3000 m/z
Ion Polarity Negative
Set Capillary 3000 V
Set End Plate Offset -500 V
Set Nebulizer 0.3 Bar
Set Dry Heater 200 °C
Set Dry Gas 3.5 l/min
Set Divert Valve Waste
Intens. x10^4
-MS, 1.8-3.1min #53-92
TP[3]COOH
473.1057
473.6071
473.8250
474.1107
474.6118
474.8700
475.1190
475.3192
475.6142
475.8638
C60H34O12, , -473.1031
2- 473.1031
2- 473.6048
2- 474.1063
2- 474.6078
m/z

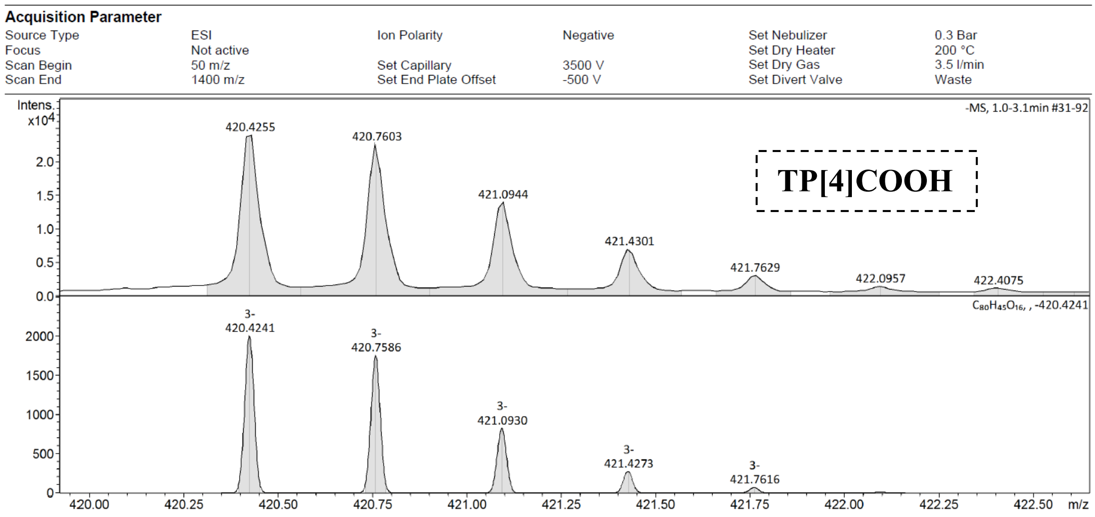

Acquisition Parameter
Source Type ESI
Focus Not active
Scan Begin 50 m/z
Scan End 1400 m/z
Ion Polarity Negative
Set Capillary 3500 V
Set End Plate Offset -500 V
Set Nebulizer 0.3 Bar
Set Dry Heater 200 °C
Set Dry Gas 3.5 l/min
Set Divert Valve Waste
Intens. x10^4
-MS, 1.0-3.1min #31-92
TP[4]COOH
420.4255
420.7603
421.0944
421.4301
421.7629
422.0957
422.4075
$C_{80}H_{45}O_{16}$, , -420.4241
3- 420.4241
3- 420.7586
3- 421.0930
3- 421.4273
3- 421.7616
m/z

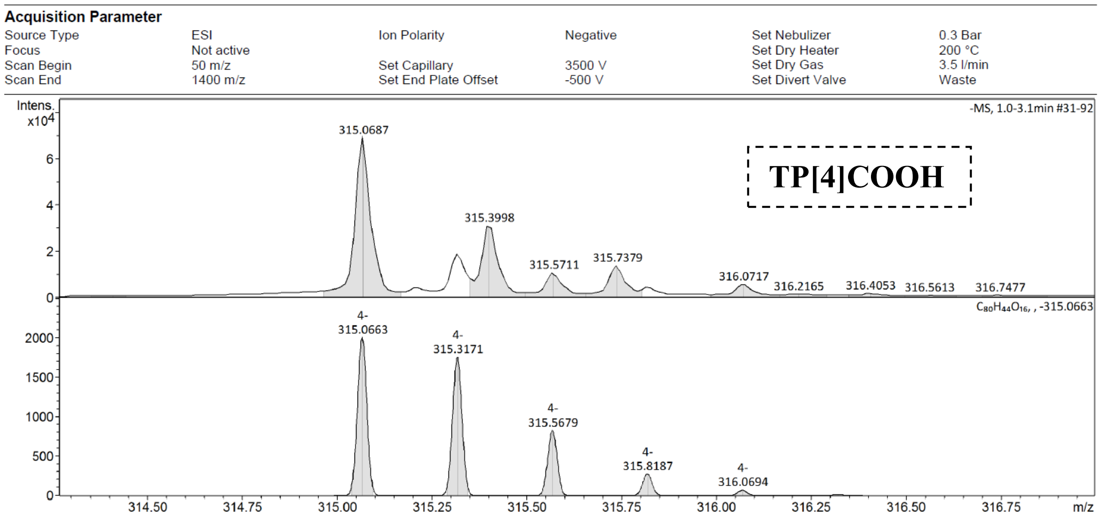

Acquisition Parameter
Source Type ESI
Focus Not active
Scan Begin 50 m/z
Scan End 1400 m/z
Ion Polarity Negative
Set Capillary 3500 V
Set End Plate Offset -500 V
Set Nebulizer 0.3 Bar
Set Dry Heater 200 °C
Set Dry Gas 3.5 l/min
Set Divert Valve Waste
Intens. x10^4
-MS, 1.0-3.1min #31-92
TP[4]COOH
315.0687
315.3998
315.5711
315.7379
316.0717
316.2165
316.4053
316.5613
316.7477
$C_{80}H_{44}O_{16}$, , -315.0663
4- 315.0663
4- 315.3171
4- 315.5679
4- 315.8187
4- 316.0694
m/z

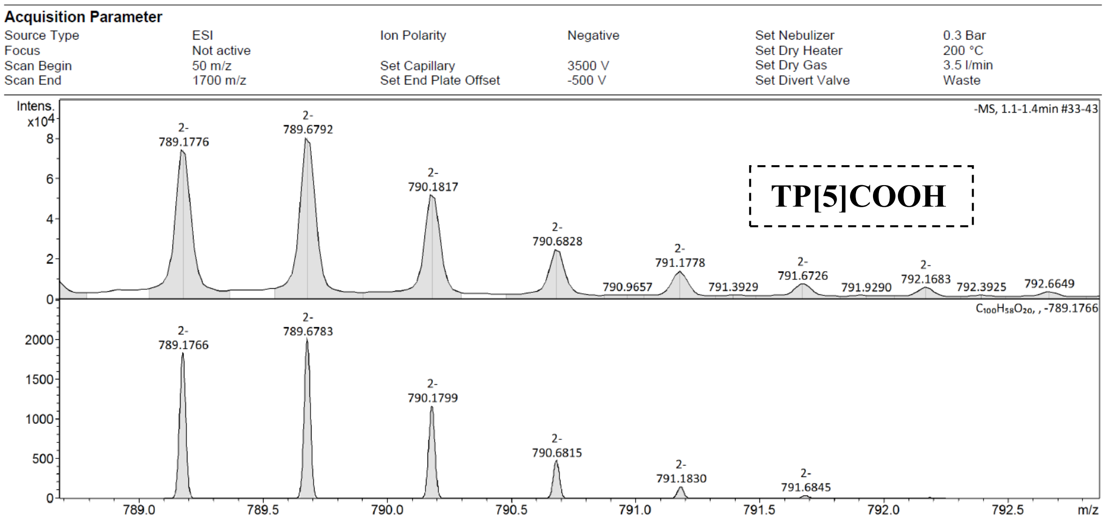

Acquisition Parameter
Source Type ESI
Focus Not active
Scan Begin 50 m/z
Scan End 1700 m/z
Ion Polarity Negative
Set Capillary 3500 V
Set End Plate Offset -500 V
Set Nebulizer 0.3 Bar
Set Dry Heater 200 °C
Set Dry Gas 3.5 l/min
Set Divert Valve Waste
Intens. x10^4
-MS, 1.1-1.4min #33-43
TP[5]COOH
2- 789.1776
2- 789.6792
2- 790.1817
2- 790.6828
790.9657
2- 791.1778
791.3929
2- 791.6726
791.9290
2- 792.1683
792.3925
792.6649
$C_{100}H_{58}O_{20}$, , -789.1766
2- 789.1766
2- 789.6783
2- 790.1799
2- 790.6815
2- 791.1830
2- 791.6845
m/z

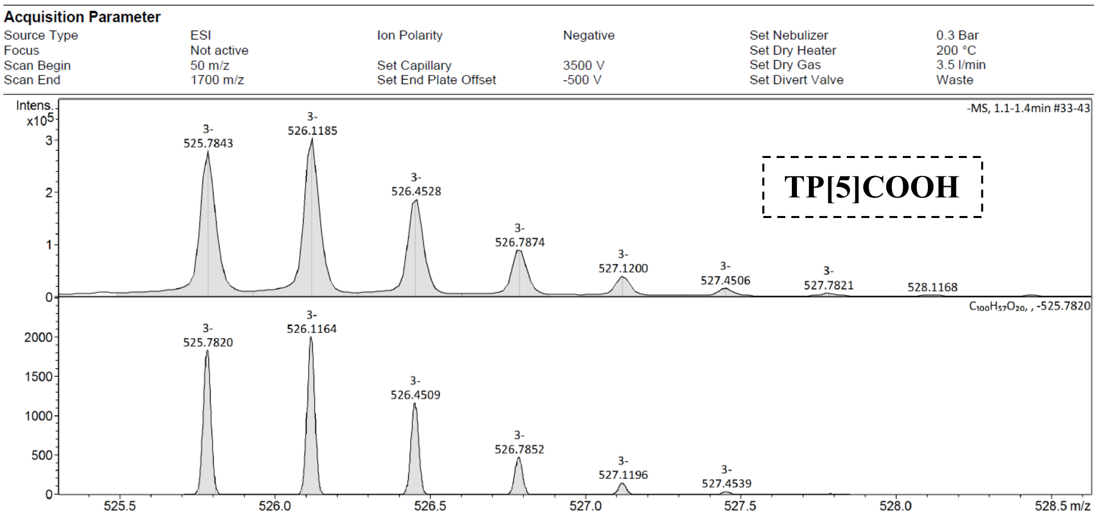

5. 1H NMR Titrations

NMR titrations were carried out on 400MHz with **TP[n]COOH** (5.0×10^{-4} M) as a host solution in a buffer containing 50mM MOPSO in D_2O pH 7.4 with 20% DMSO-d6. Guest solution aliquots were added to the host and spectra was recorded at 25 °C. Binding constants were calculated by a global analysis of all shifted peaks using the bind-fit software from *supramolecular.org* with a 1:1 and or 1:2 model. Corresponding titrations are summarized in the table below.

[MOPSO: 2-Hydroxy-3-morpholinopropanesulfonic acid]

**TP[2]COOH** with **L-arginine**	**TP[2]COOH** with **L-lysine**
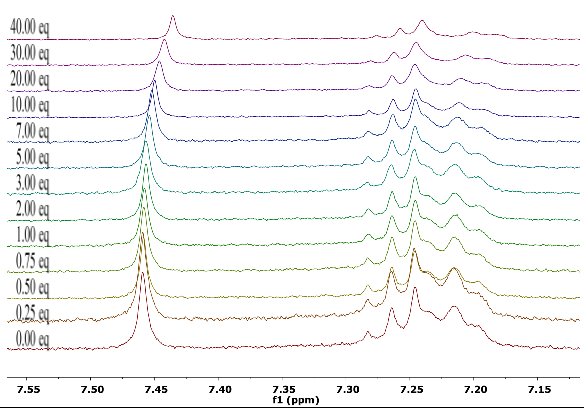	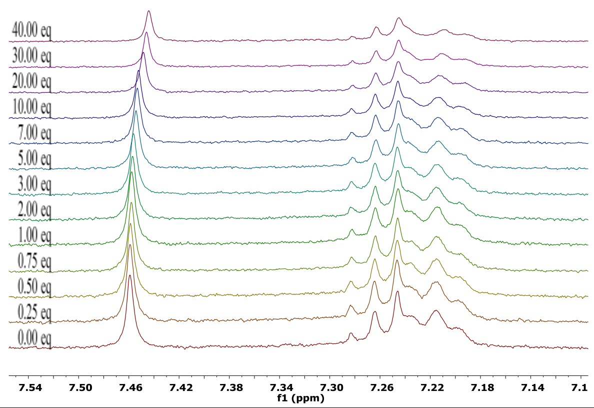

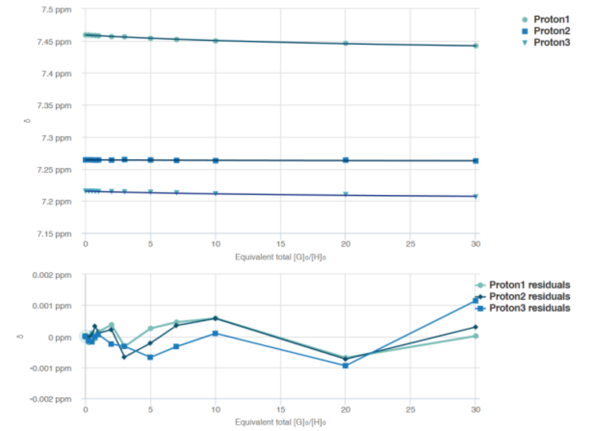

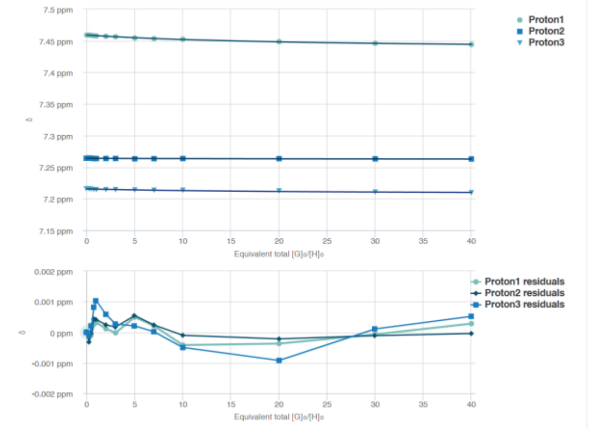

TP[2]COOH with **Histamine**

TP[3]COOH with **Dopamine**

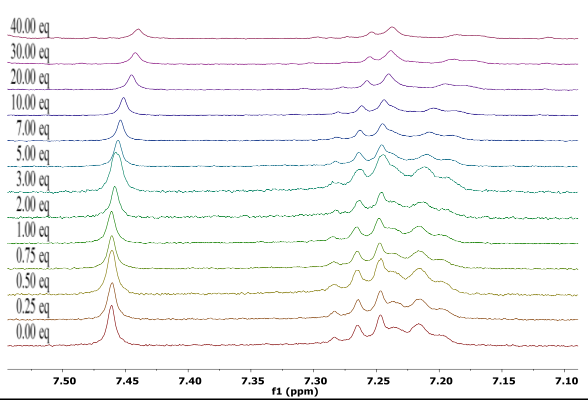

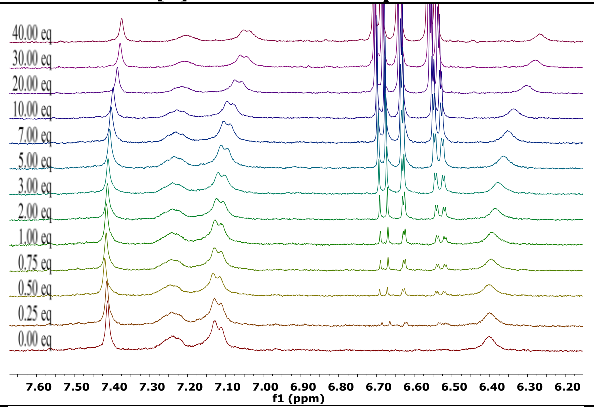

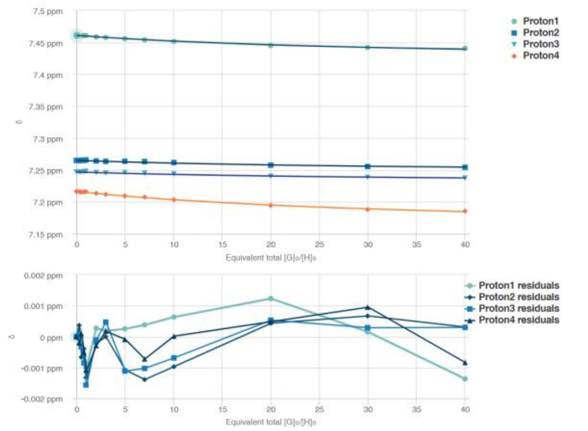

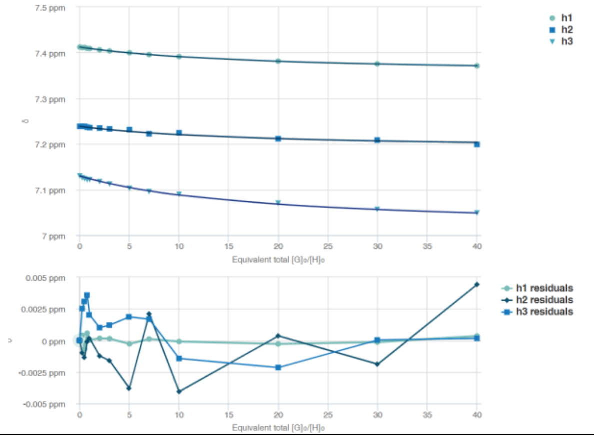

TP[3]COOH with **L-dopa**

TP[3]COOH with **Histamine**

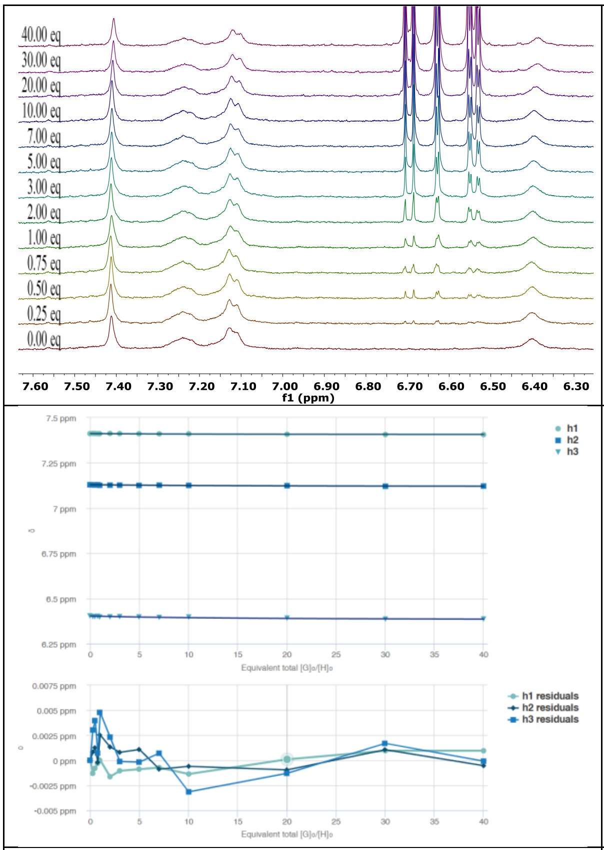

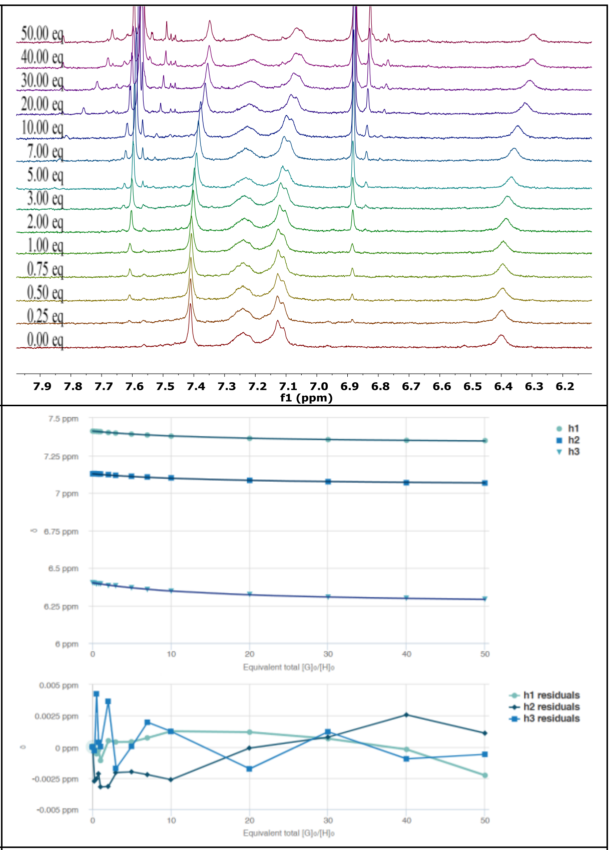

**TP[3]COOH with L-histidine**	**TP[3]COOH with L-alanine**

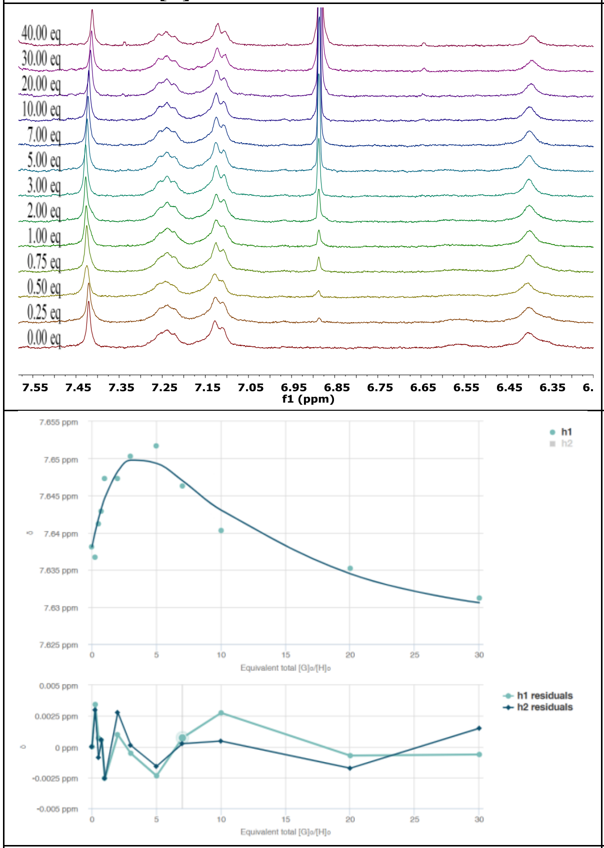

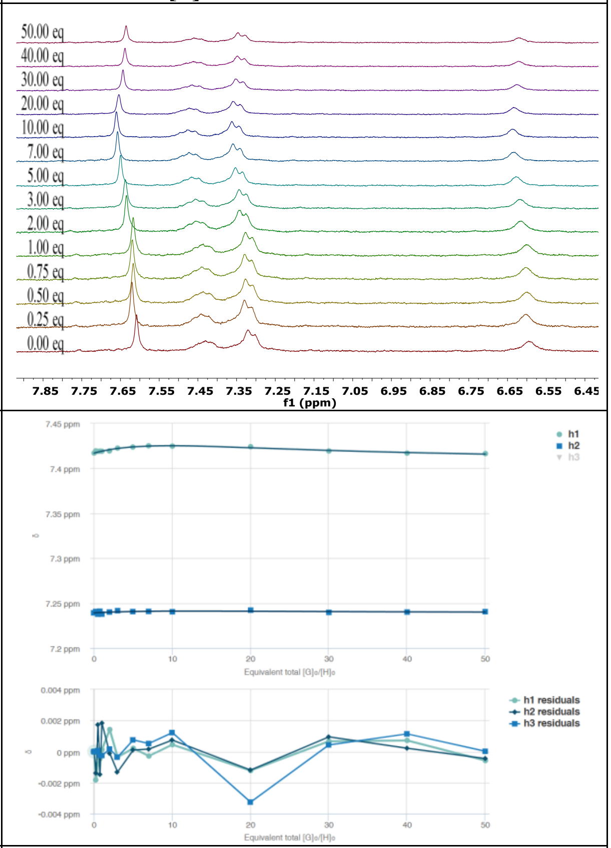

**TP[3]COOH with L-phenylalanine**	**TP[3]COOH with Serotonin**

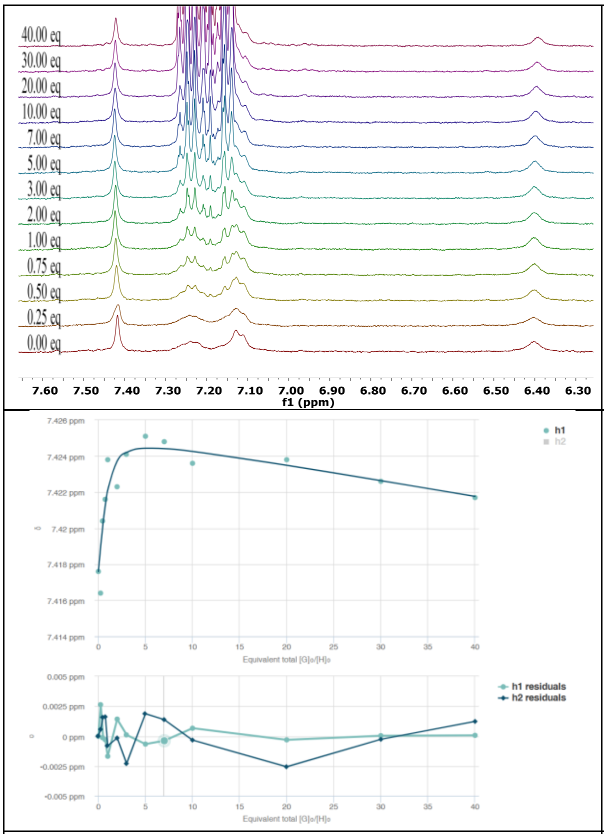

TP[3]COOH with **L-tryptophan**

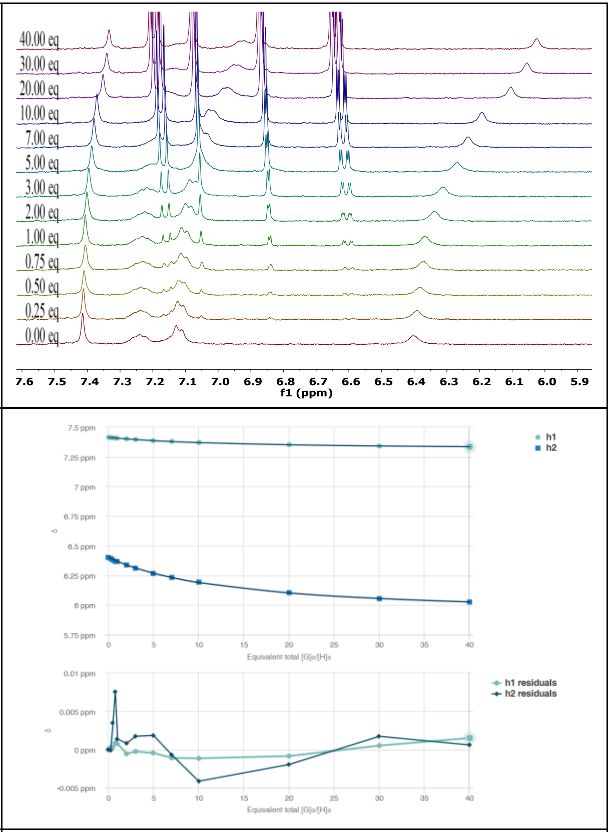

TP[3]COOH with **L-tyrosine**

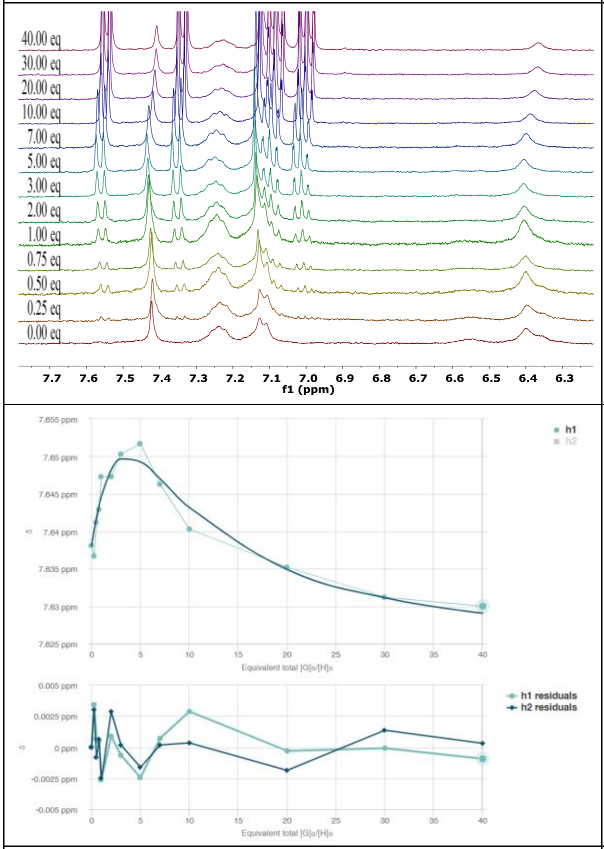

TP[3]COOH with **L-lysine**

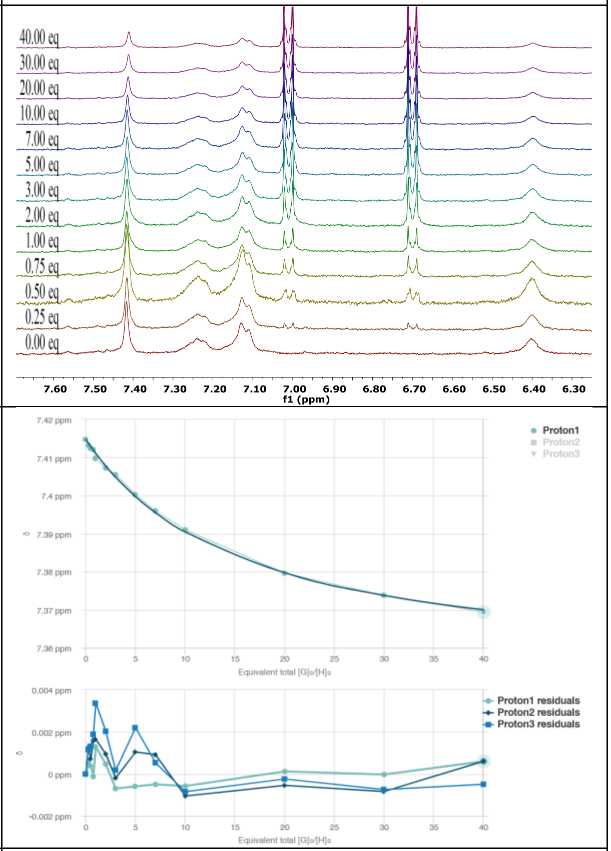

TP[3]COOH with **L-arginine**

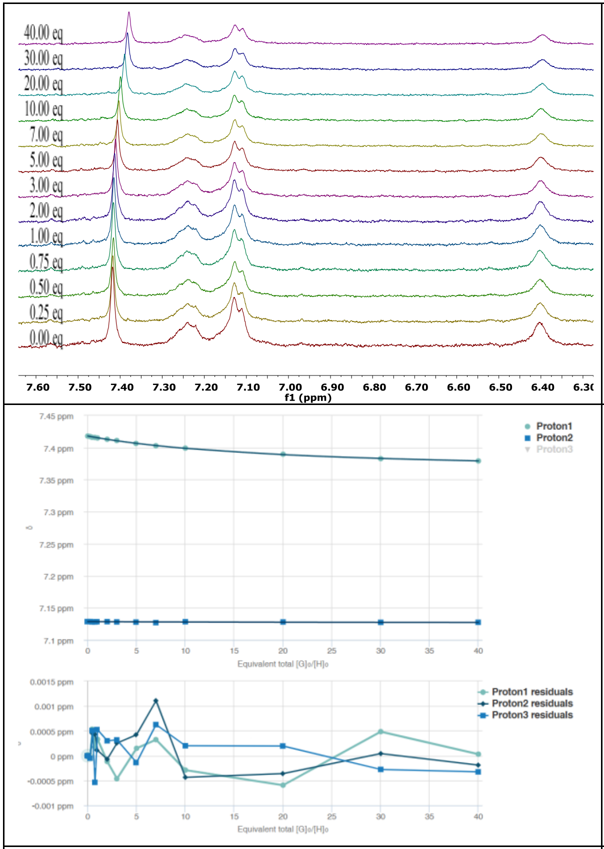

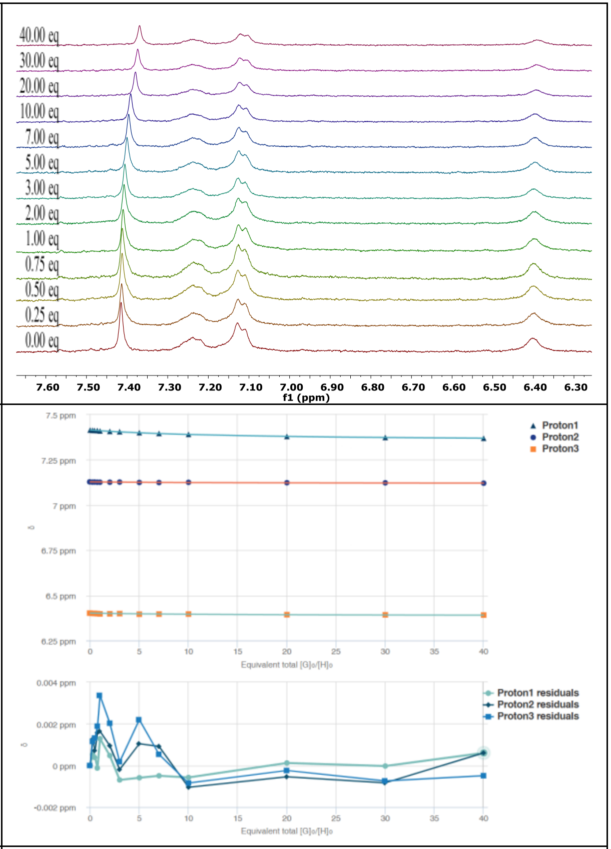

TP[4]COOH with **Dopamine**

TP[4]COOH with **L-dopa**

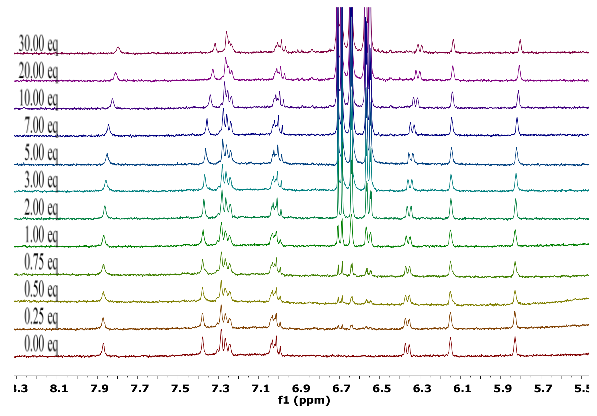

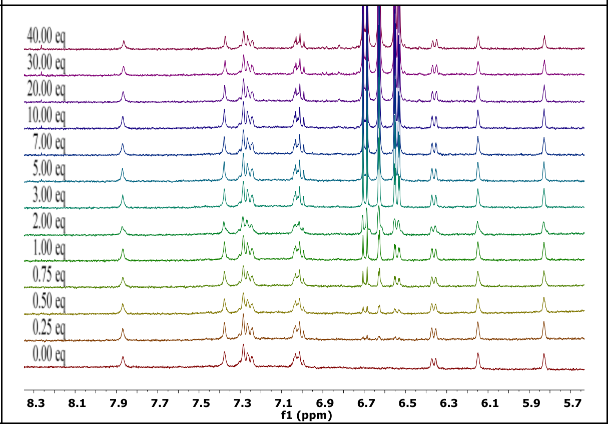

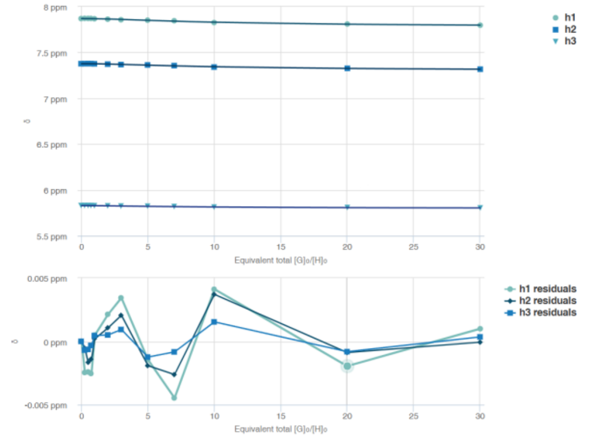

no shift

TP[4]COOH with **Histamine**

TP[4]COOH with **L-histidine**

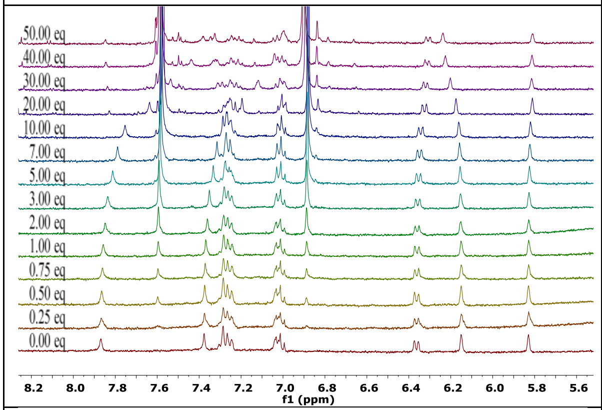

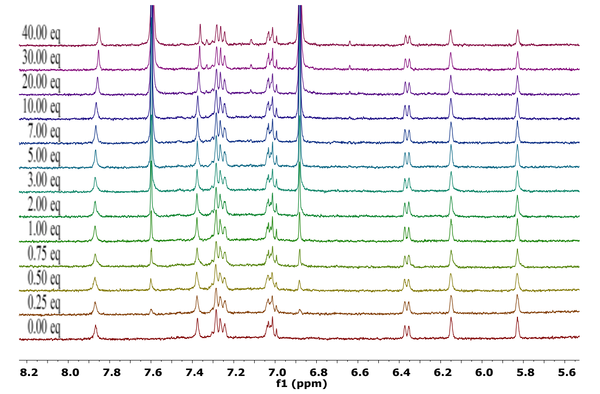

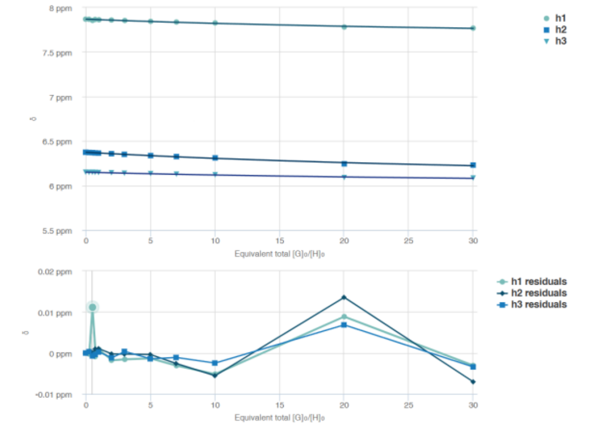

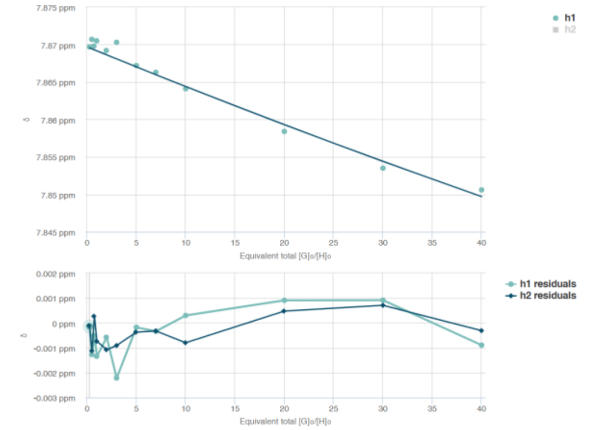

TP[4]COOH with **L-alanine**

TP[4]COOH with **L-phenylalanine**

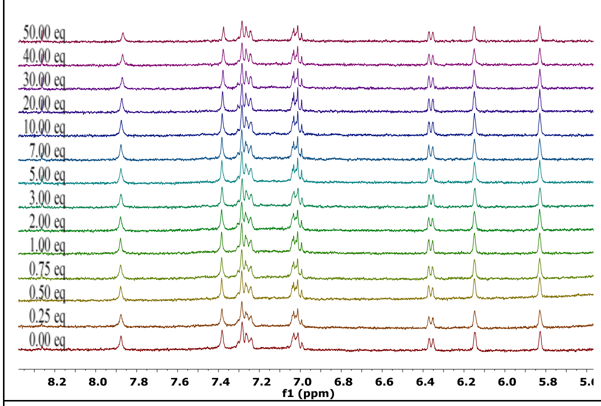

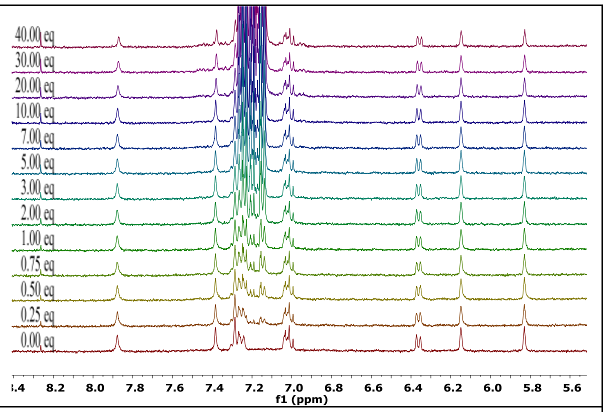

No shift | No shift

TP[4]COOH with **Serotonin** | **TP[4]COOH** with **L-tryptophan**

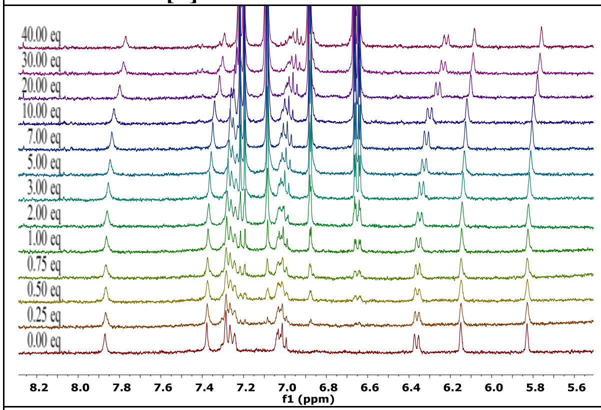

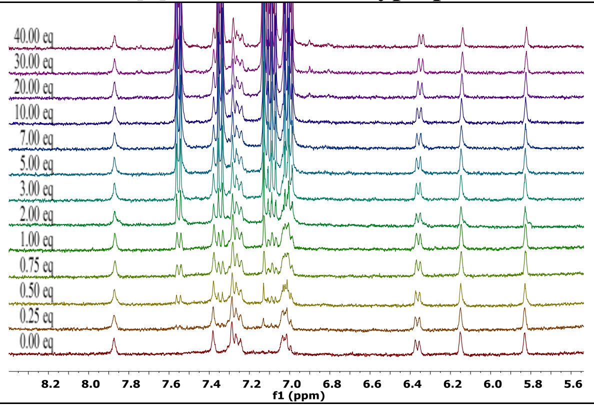

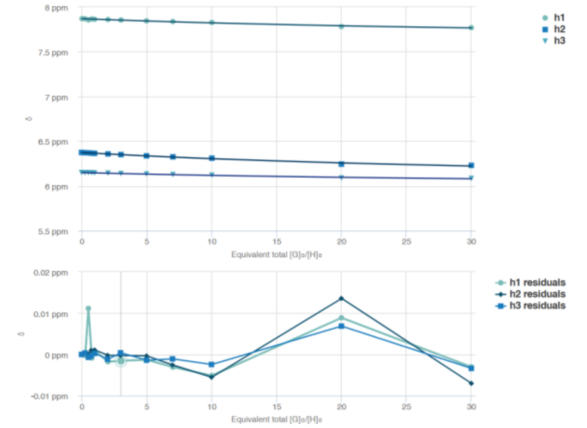

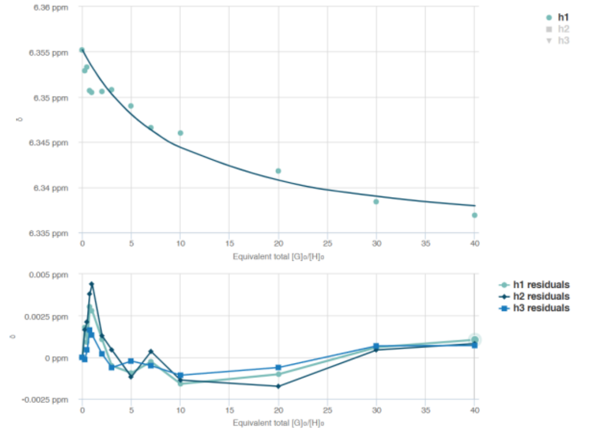

TP[4]COOH with **L-lysine** | **TP[4]COOH** with **L-arginine**

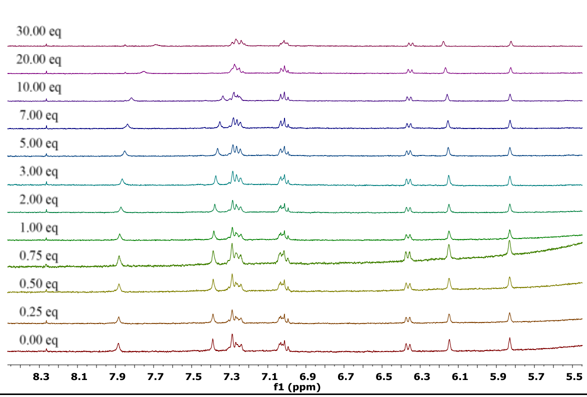

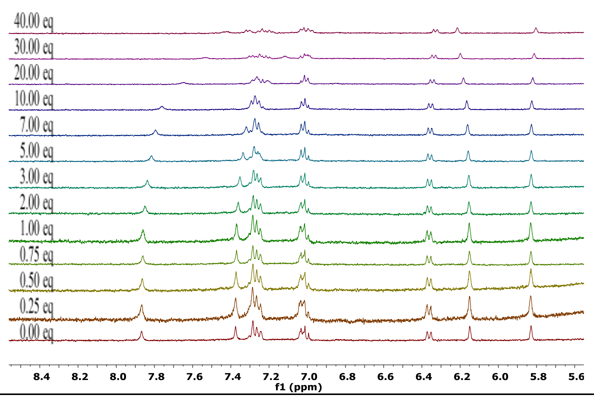

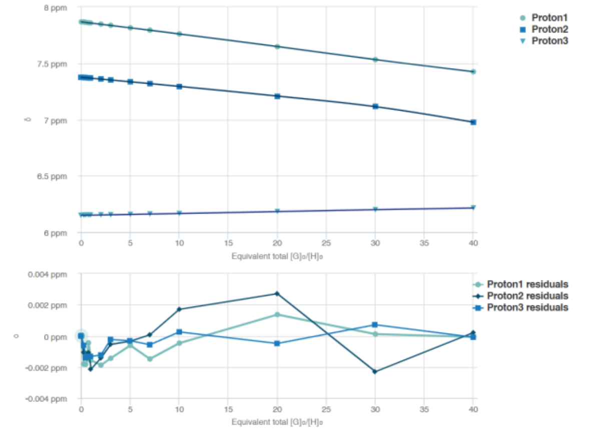	--weak binding--
**TP[3]COOH** with **L-γ-Glu-Phe-OH**	
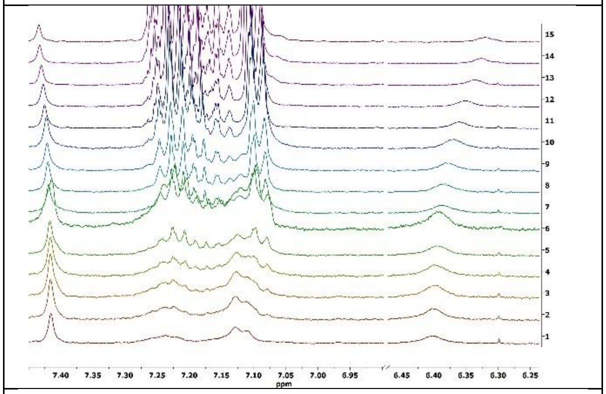	
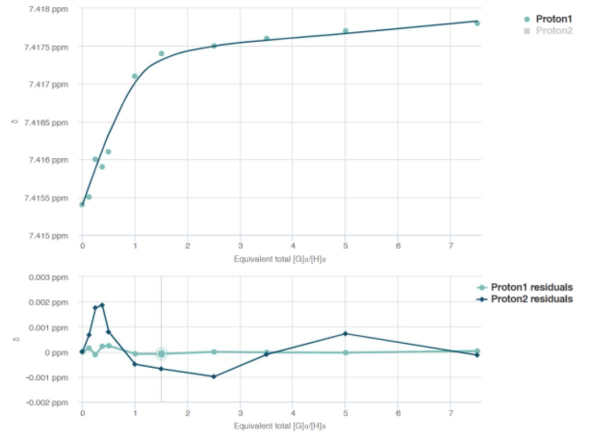	

6. Thioflavin T Assay

Thioflavin T (ThT) Assay with TP[n]COOH

All fluorescence measurements were carried out with the constant ThT (0.010mM) concentrations, while increasing the concentration of TP[n]COOH (1.0mM) up to 60eq. The following buffers and the setup parameters were used for fluorescence titration experiments:

a) **pH 2 Buffer:** 25mM HCl, 100 mM NaCl, 5%DMSO

b) **pH 7.4 Buffer**: 20mM SPB, 30mM NaCl, 5%DMSO

The resulting data were imported into the HypSpec program and fitted to obtain stability constants for the host and guest using a 1:1 and 2:1 binding model.

[SPB: sodium phosphate buffer]

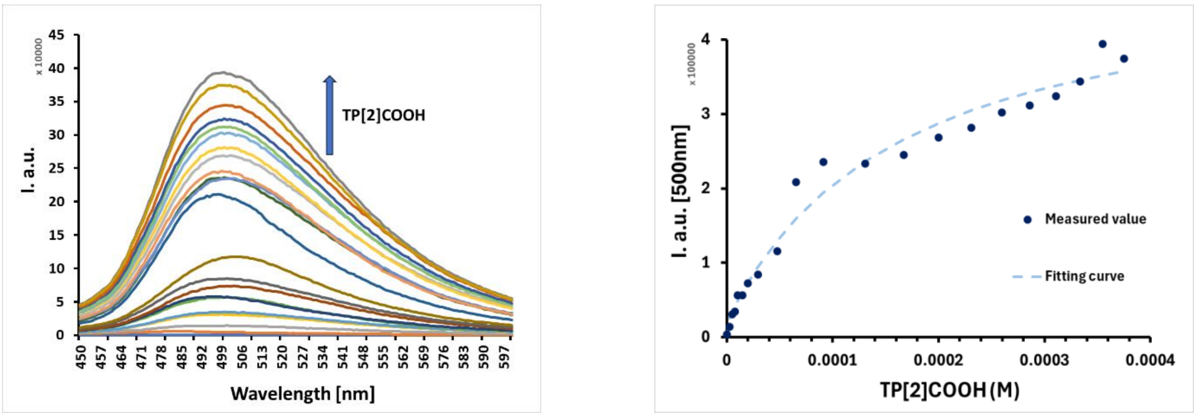

Figure S40. Fluorescence titration of TP[2]COOH (1.0mM) into ThT (0.010mM), λex 415nm, Slit 3-3 at pH 2.0 and the fitting curve.

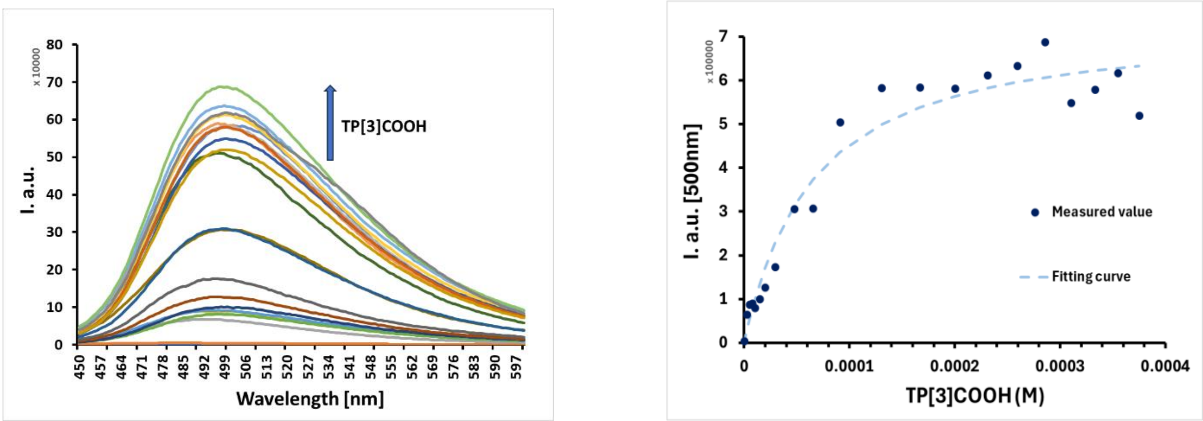

Figure S41. Fluorescence titration of TP[3]COOH (1.0mM) into ThT (0.010mM), λex 415nm, Slit 3-3 at pH 2.0 and the fitting curve.

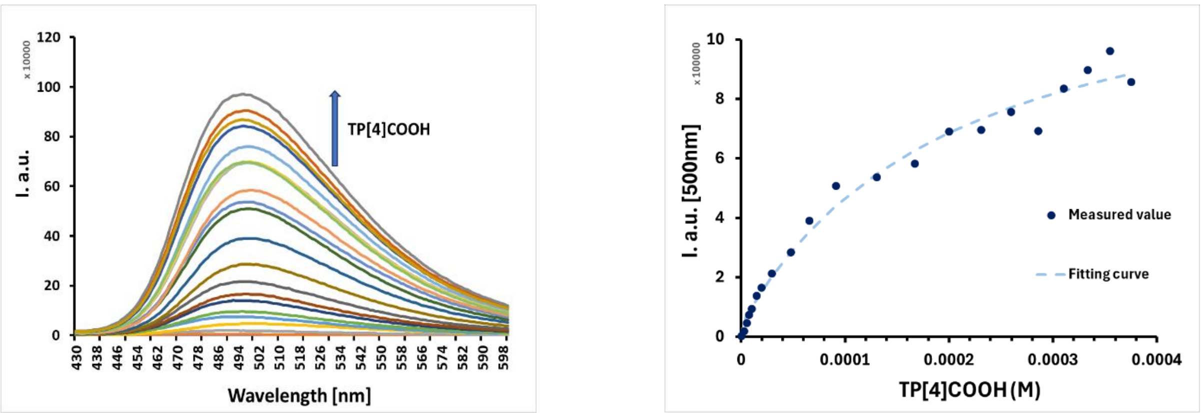

Figure S42. Fluorescence titration of TP[4]COOH (1.0mM) into ThT (0.010mM), λex 415nm, Slit 3-3 at pH 2.0 and the fitting curve.

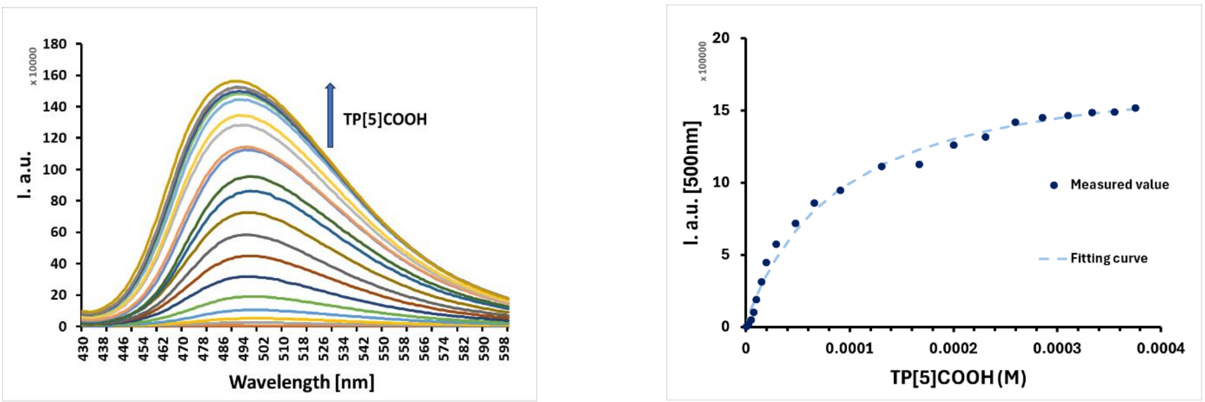

Figure S43. Fluorescence titration of TP[5]COOH (1.0mM) into ThT (0.010mM), λex 415nm, Slit 3-3 in at pH 2.0 and the fitting curve.

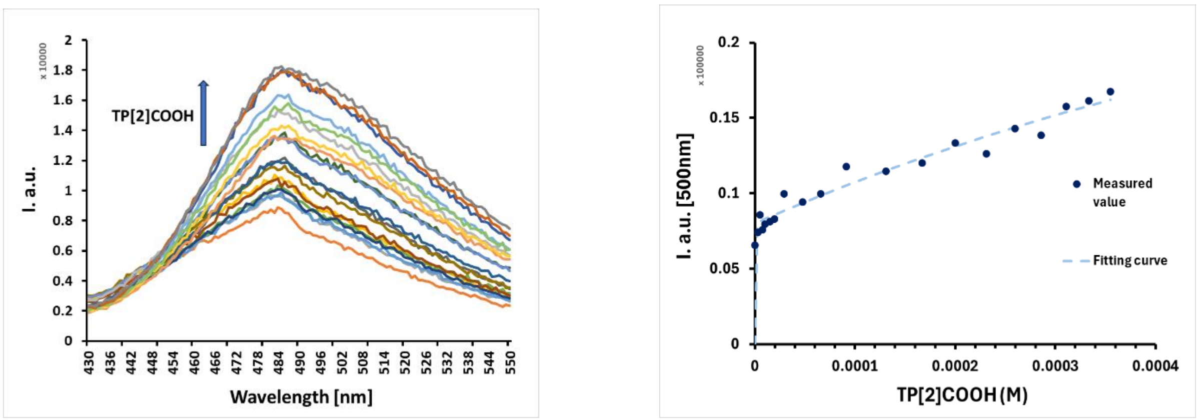

Figure S44. Fluorescence titration of TP[2]COOH (1.0mM) into ThT (0.010mM), λex 415nm, Slit 3-3 in SPB pH 7.4 and the fitting curve.

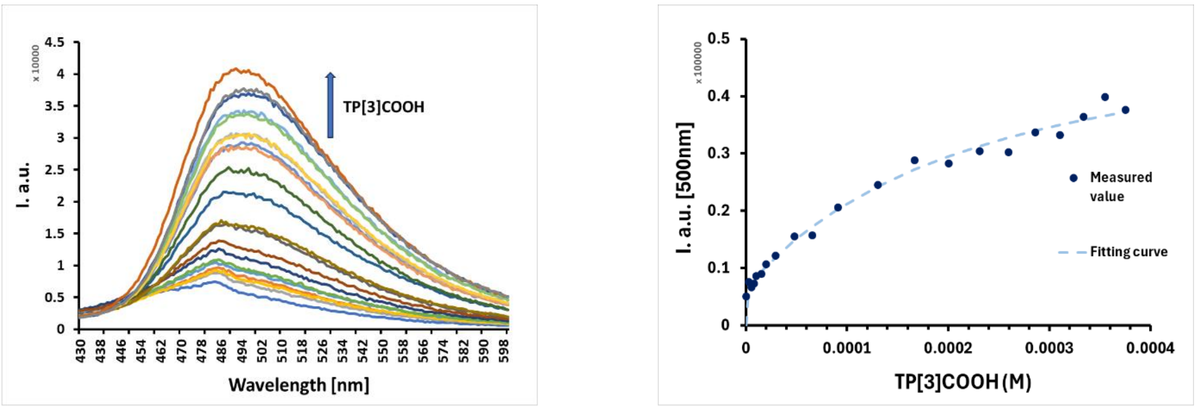

Figure S45. Fluorescence titration of TP[3]COOH (1.0mM) into ThT (0.010mM), λex 415nm, Slit 3-3 in SPB pH 7.4 and the fitting curve.

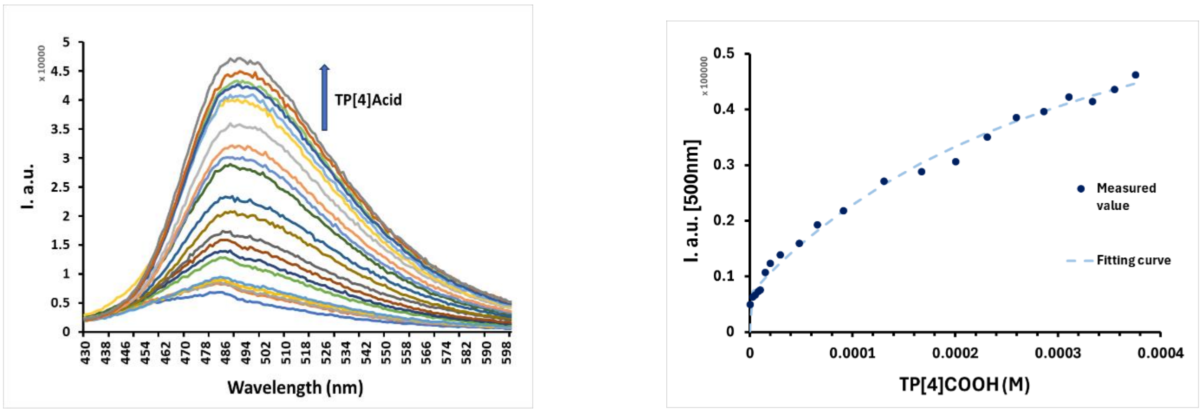

Figure S46. Fluorescence titration of TP[4]COOH (1.0mM) into ThT (0.010mM), λex 415nm, Slit 3-3 in SPB pH 7.4 and the fitting curve.

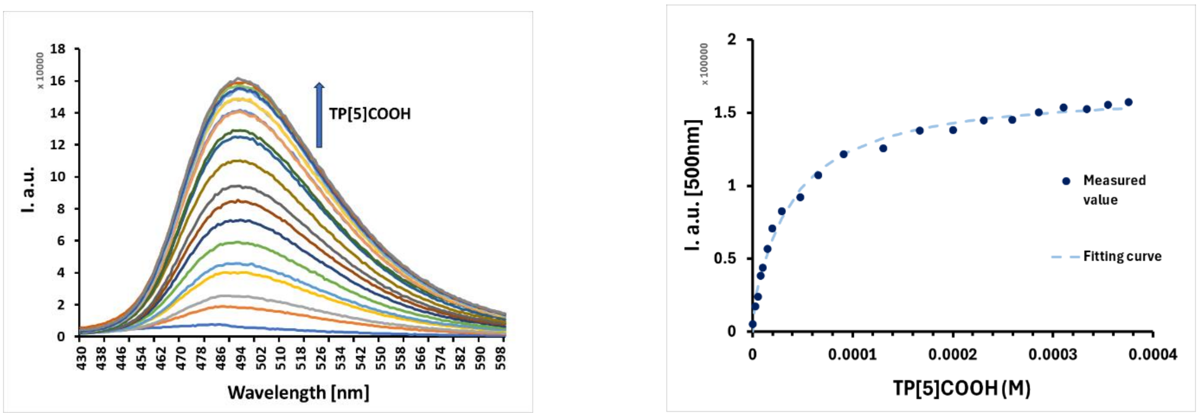

Figure S47. Fluorescence titration of TP[5]COOH (1.0mM) into ThT (0.010mM), λex 415nm, Slit 3-3 in SPB pH 7.4 and the fitting curve.

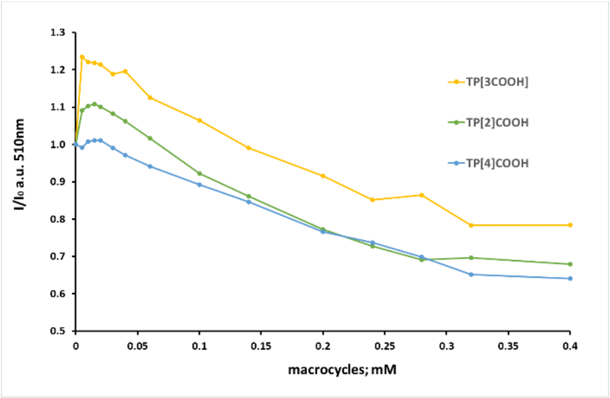

Figure S48. Displacement experiment: insulin (0.1 mM) was premixed with ThT (0.01 mM) at pH 2.0 and then titrated with macrocyclices. The emission of ThT was recorded.

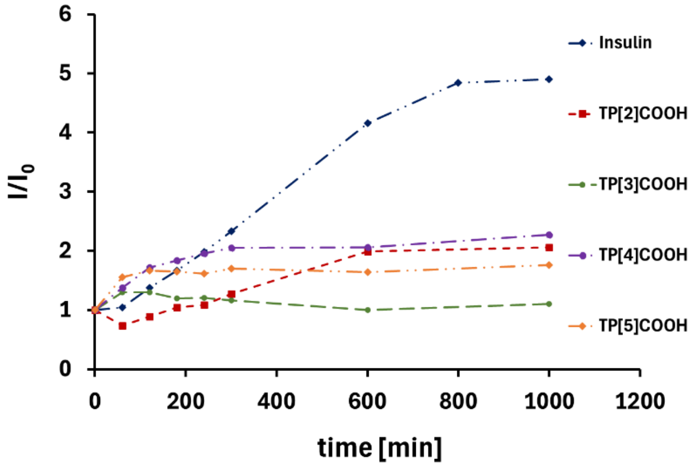

Figure S49. Aggregation kinetic of insulin (50 μM) in the presence of macrocycles (50 μM) as determined by fluorescence assay with ThT (1 mM) at 50°C. Conditions: phosphate buffer, pH 7.4, 0.1 M NaCl, 5% DMSO.

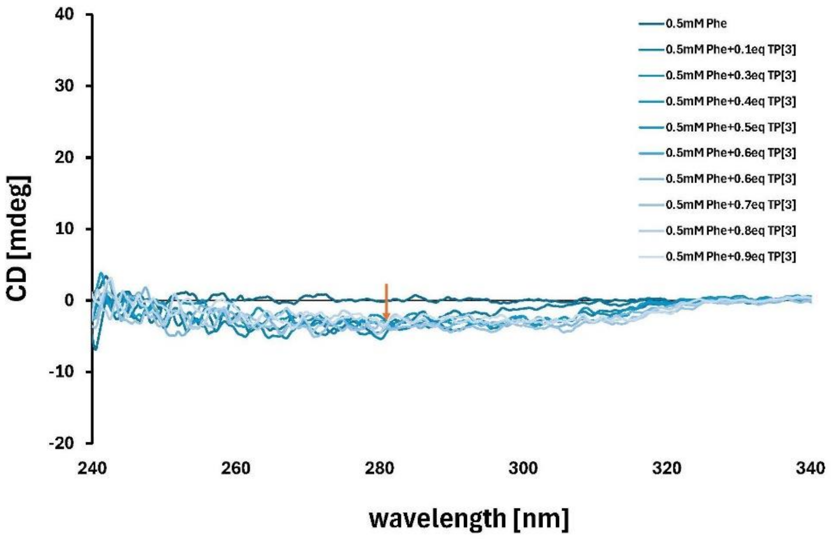

Figure S50. CD changes observed in the region of TP[3]COOH absorption during the stepwise addition of TP[3]COOH to the 0.5 mM solution of Phe in the phosphate buffer.

7. Isothermal Titration Calorimetry (ITC)

Titration experiments were carried out at 25 °C. The insulin was in the sample cell with volume 350 μL and TP[n]COOH was in the injection syringe with a volume containing 50 μL. Phosphate buffer (20 mM, pH 7.4) was used as a solvent containing 20% v/v DMSO. The titration schedule consisted of 20 consecutive injections of 2.46 μL with an interval of 200 s between injections. The respective first injections were deleted from the data sheet to eliminate the effect of titrant diffusion across the syringe tip during the equilibration process. Heat of dilution was subtracted from all samples investigated. Each experiment was performed in duplicate, and the experimental data were fitted to a theoretical titration curve using the

software AFFINImeter with ΔH (enthalpy change in kcal mol-1), K_a (association constant in M^{-1}) and n (stoichiometry), as adjustable parameters.

Thermodynamic parameters were calculated from equation:

$$\Delta G = \Delta H - T\Delta S = -RTlnK_a$$

TP[n]COOH	K_a (M^{-1})	ΔG[b] (kcal/mol)	ΔH[c] (kcal/mol)	-TΔS[d] (kcal/mol)
TP[3]COOH	K_{11} = 6.1 (±0.4)x10^3	-5.20 (±0.03)	-24.0 (±2)	18.8 (±2)
TP[4]COOH	K_{11} = 1.3 (±0.3)x10^3	-4.25 (±0.14)	-300 (±100)	295 (±100)
TP[5]COOH	K_{11} = 3.4 (±0.2)x10^4	-6.18 (±0.03)	-13.0 (±0.5)	6.82(±0.5)

[a] Mean value measured from at least three ITC experiments at 25 °C. Standard deviations are given in parentheses. [b] Gibbs free energy values calculated from K_a values. Standard deviations for ΔG values were calculated as the relative error observed in K_a, due to their relationship by a natural logarithm. [c] Enthalpy values measured by ITC. [d] Entropic contributions to ΔG calculated from Ka and ΔH values.

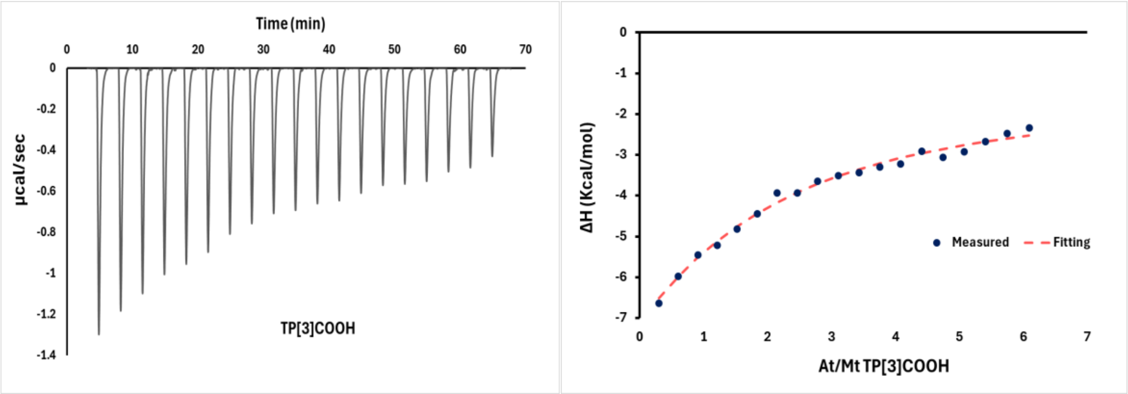

Figure S51: ITC titration of TP[3]COOH (2.14 mM) into Insulin (0.05 mM).

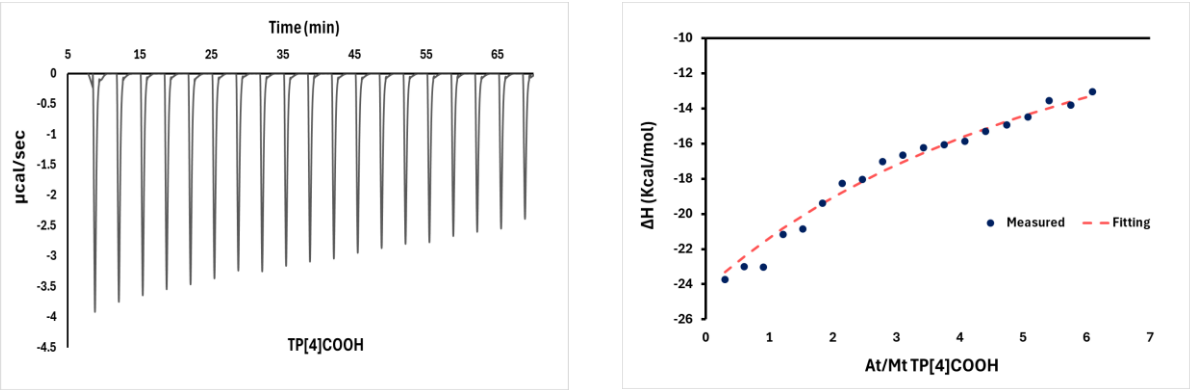

Figure S52: ITC titration of TP[4]COOH (2.14 mM) into Insulin (0.05 mM).

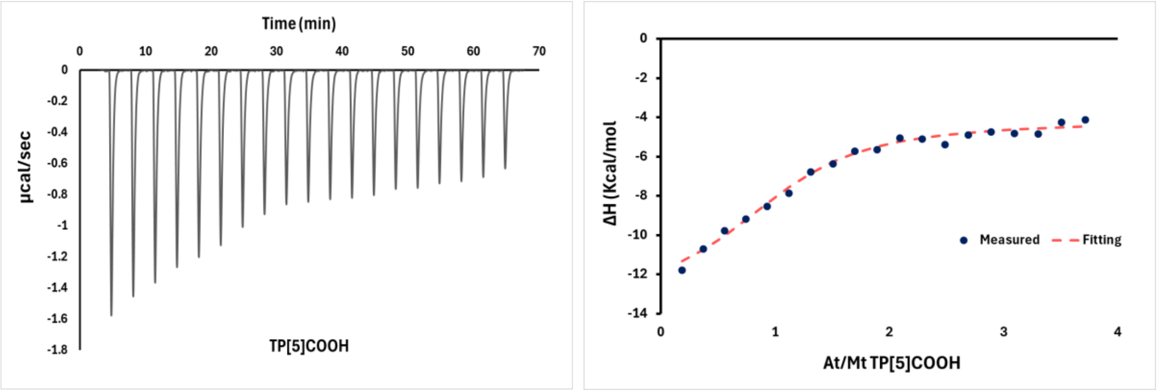

Figure S53: ITC titration of TP[5]COOH (1.30 mM) into Insulin (0.05 mM).

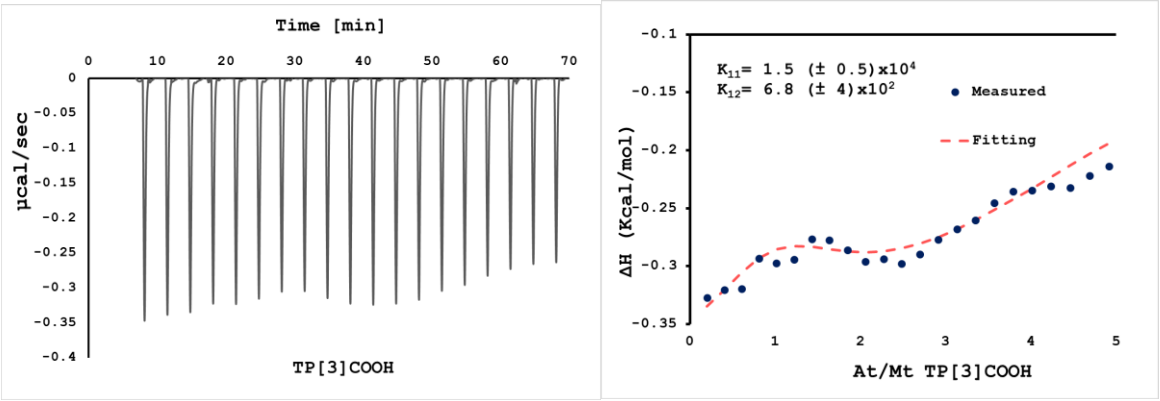

Figure S54. ITC titration of TP[3]COOH (0.3 mM) with Phe (10.5 mM).

8. Crystal Data and Experimental

Single clear light colourless block-shaped crystals of TP[2]COOEt recrystallised from a mixture of MeOH and diethylether by solvent layering. A suitable crystal with dimensions 0.21 × 0.13 × 0.10 mm^3 was selected and mounted on a mylar loop in per¬fluoro¬ether oil on a SuperNova, Dual, Cu at home/near, Atlas diffractometer. The crystal was kept at a steady T = 153.1(6) K during data collection. The structure was solved with the ShelXT 2018/2 (Sheldrick, G.M., Crystal structure refinement with ShelXL, Acta Cryst., (2015), C71, 3-8.) solution program using dual methods and by using Olex2 1.5 (O.V. Dolomanov and L.J. Bourhis and R.J. Gildea and J.A.K. Howard and H. Puschmann, Olex2: A complete structure solution, refinement and analysis program, J. Appl. Cryst., (2009), 42, 339-341.) as the graphical interface. The model was refined with ShelXL 2018/3 (Sheldrick, G.M., ShelXT-Integrated space-group and crystal-structure determination, Acta Cryst., (2015), A71, 3-8.) using full matrix least squares minimisation on F2. $C_{49}H_{44}O_9$, M_r = 776.84, triclinic, *P*-1 (No. 2), a = 8.8406(5) Å, b = 13.4188(10) Å, c = 17.8956(10) Å, ▯ = 83.230(5)°, ▯ = 78.672(5)°, ▯ = 75.828(6)°, *V* = 2012.8(2) Å^3, *T* = 153.1(6) K, *Z* = 2, *Z'* = 1, ▯(Cu K▯) = 0.712, 14210 reflections measured, 7588 unique (R_{int} = 0.0439) which were used in all calculations. The final wR_2 was 0.1338 (all data) and R_1 was 0.0485 (I≥2 ▯(I)).

Single clear light colourless prism-shaped crystals of TP[2]CH_3 recrystallised from a mixture of toluene and ethanol by solvent layering. A suitable crystal with dimensions 0.06 × 0.06 × 0.05 mm^3 was selected $C_{40}H_{32}$, M_r = 512.65, monoclinic, *P*2/*c* (No. 13), a = 16.6831(2) Å, b = 10.4430(2) Å, c = 16.9317(2) Å, ▯ = 110.063(2)°, ▯ = ▯ = 90°, *V* = 2770.86(8) Å^3, *T* = 100.00(10) K, *Z* = 4, *Z'* = 1, ▯(Cu K▯) = 0.522, 20445 reflections measured, 5404 unique (R_{int} = 0.0256) which were used in all calculations. The final wR_2 was 0.1238 (all data) and R_1 was 0.0443 (I≥2 ▯(I)).

Single clear light colourless block-shaped crystals of TP[3]CH_3 recrystallised from a mixture of DCM and n-hexane by solvent layering. A suitable crystal with dimensions 0.30 × 0.10 × 0.05 mm^3 was selected. $C_{63.6}H_{56.4}$, M_r = 820.68, monoclinic, *P*2$_1$/*n* (No. 14), a = 10.4812(3) Å, b = 18.3917(5) Å, c = 24.3647(9) Å, ▯ = 89.070(4)°, ▯ = ▯ = 90°, *V* = 4696.1(3) Å^3, *T* = 155(4) K, *Z* = 4, *Z'* = 1, ▯(Cu K▯) = 0.490, 11707 reflections measured, 6957 unique (R_{int} = 0.0393) which were used in all calculations. The final wR_2 was 0.2471 (all data) and R_1 was 0.0857 (I≥2 ▯(I)).

Single clear light colourless block-shaped crystals of TP[3]COOEt recrystallised from a mixture of MeOH and DCM/Et2O by solvent layering. A suitable crystal with dimensions 0.26 × 0.20 × 0.17 mm^3 was selected. $C_{72}H_{60}O_{12}$, M_r = 1117.20, monoclinic, *P*2$_1$/*n* (No. 14), a = 17.85910(10) Å, b = 17.95320(10) Å, c = 18.15320(10) Å, ▯ = 105.5520(10)°, ▯ = ▯ = 90°, *V* = 5607.32(6) Å^3, *T* = 100.00(10) K, *Z* = 4, *Z'* = 1, ▯(Cu K▯) = 0.725, 53762 reflections measured, 11192 unique (R_{int} = 0.0235) which were used in all calculations. The final wR_2 was 0.0968 (all data) and R_1 was 0.0386 (I≥2 ▯(I)).

Single clear light colourless plate-shaped crystals of 25Kat_SG01 recrystallised from a mixture of DCM/Hexane and MeOH by solvent layering. A suitable crystal with dimensions 0.22 × 0.19 × 0.04 mm^3 was selected. $C_{172}H_{156}Cl_4$, M_r = 2364.76, monoclinic, *P*2$_1$/*c* (No. 14), a = 21.0636(4) Å, b = 16.5000(2) Å, c = 20.1830(3) Å, ▯ = 107.2338(16)°, ▯ = ▯ = 90°, *V* = 6699.66(18) Å^3, *T* = 100.00(10) K, *Z* = 2, *Z'* = 0.5, ▯(Cu K▯) = 1.210, 63953 reflections measured, 13461 unique (R_{int} = 0.0561) which were used in all calculations. The final wR_2 was 0.2273 (all data) and R_1 was 0.0747 (I≥2 ▯(I)).

9. DFT Calculations

Molecular electrostatic potential mapped to the electron density of the complex TP[3]COO^- and phenylalanine. Isosurface value = 0.001 a.u.

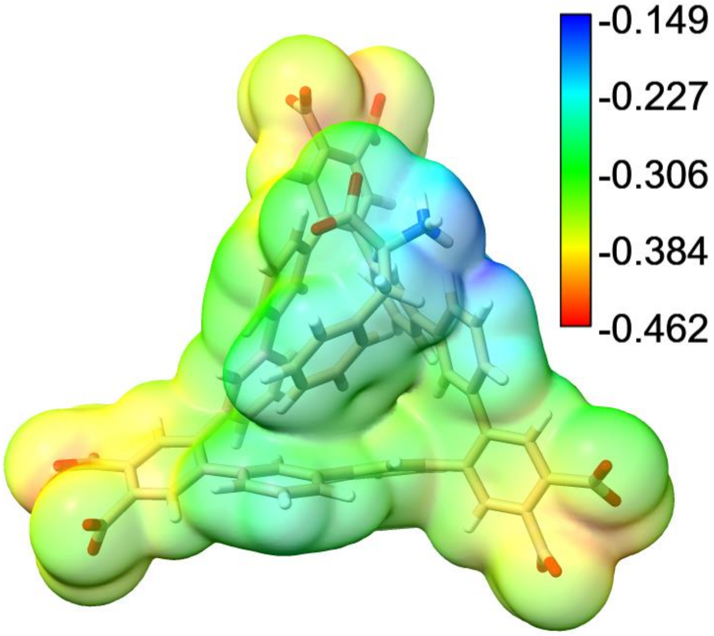

The geometry of the optimized complexes is given in xyz coordinates.

Optimized structure of TP[2]COO]$^{4-}$

Coordinates from ORCA-job E= -2138.482606201338

C	2.734040	-0.871004	1.515949
C	2.255621	0.011132	0.542709
H	2.065229	1.046339	0.810199
C	1.926750	-0.432413	-0.741647
C	2.154405	-1.773473	-1.076509
H	1.910377	-2.131364	-2.073174
C	2.692223	-2.643390	-0.135382
H	2.875395	-3.680708	-0.401286
C	2.965794	-2.204805	1.156839
H	3.327244	-2.908434	1.901765
C	2.944196	-0.440059	2.921157
C	4.128612	-0.828494	3.557875
H	4.856109	-1.410416	2.997027
C	4.419720	-0.484959	4.874707
C	3.495444	0.282742	5.597445
C	2.313585	0.672984	4.973240
H	1.592982	1.256724	5.540450
C	2.009785	0.333923	3.650292
C	5.697775	-1.010423	5.493645
O	6.782640	-0.725621	4.910080
O	5.577841	-1.734613	6.523003
C	3.773926	0.751316	7.009914
O	4.925703	1.220283	7.240196
O	2.828998	0.671135	7.847773
C	0.724321	0.814485	3.083539
C	0.357679	2.157876	3.232245
H	1.000890	2.833246	3.789920
C	-0.799599	2.642455	2.629241
H	-1.072085	3.687178	2.750283
C	-1.588819	1.808796	1.845712
H	-2.471475	2.201676	1.348327
C	-1.248070	0.458266	1.696926
C	-0.112506	-0.030846	2.349483
H	0.164159	-1.073146	2.220515
C	-2.784447	-0.797055	-1.511391
C	-2.295988	0.054335	-0.515783
H	-2.093076	1.093766	-0.756271
C	-1.972083	-0.425989	0.756677
C	-2.216324	-1.772014	1.057816
H	-1.976291	-2.157577	2.045072

C	-2.764718	-2.611258	0.095101
H	-2.960640	-3.652691	0.334496
C	-3.031897	-2.136815	-1.185559
H	-3.401145	-2.817608	-1.947777
C	-2.986181	-0.330821	-2.906574
C	-4.166456	-0.703424	-3.561003
H	-4.903298	-1.288101	-3.015641
C	-4.455071	-0.315744	-4.866125
C	-3.528370	0.474962	-5.560021
C	-2.350508	0.849666	-4.919911
H	-1.620609	1.435905	-5.472751
C	-2.046279	0.459638	-3.610609
C	-5.794988	-0.695457	-5.460228
O	-6.511005	0.246833	-5.905656
O	-6.114250	-1.918626	-5.430358
C	-3.732717	0.869074	-7.007412
O	-3.536036	2.081535	-7.309620
O	-4.049627	-0.052592	-7.813063
C	-0.753349	0.905501	-3.032823
C	-0.368487	2.247478	-3.145428
H	-1.002696	2.946783	-3.683681
C	0.795888	2.699759	-2.531481
H	1.082500	3.743532	-2.624757
C	1.574386	1.834502	-1.771733
H	2.462857	2.201567	-1.265039
C	1.215086	0.485441	-1.658636
C	0.072504	0.029375	-2.322756
H	-0.218034	-1.012170	-2.221171

Optimized structure of TP[3]COO]$^{6-}$

O	14.50129792783082294250	-1.58701880985098076060	12.16213896682964978879
O	12.96139498223221586670	-2.58798098952670718731	10.89169519886509895912
O	12.18751690342011606560	-2.17684618218410186330	15.34244481227468170914
O	10.64608965758123027001	-3.15651524468389999001	14.05969565805199117392
O	0.28212828326277311630	10.38077191603122884089	11.67899044408288844465
O	1.19458380739844893803	10.77760948235302329579	13.67629145625023312505
O	0.03709969859734264103	6.41402637261992492057	11.90722008659995267976
O	0.45575247362054510658	6.86833349923966718364	14.05355699076482878240
O	11.08911684812062325989	12.07267774439886132143	3.34383879577723019949
O	12.77680691217352304534	8.47811167576710822402	1.99153742039365266869
O	10.58802900104983635288	8.52988747980754524747	1.55671001061711944402
C	6.13263821247599594244	5.67438921837276577520	12.41382219831096911378
H	5.84658442918078780082	5.78916716217456261973	13.45023341411818584845
C	5.42635098596107745550	6.39853796428677323860	11.46080317736798548367
C	5.72740805438372735381	6.20564336783876235870	10.11321199244016888485
H	5.14541279048913846594	6.72631991050703526014	9.36383073312430802559
C	6.77004761713959979375	5.37832255561298921975	9.74373023131790105822
H	7.03485024171972561646	5.26578427496874112990	8.70071756687954334097
C	7.50435442228527449515	4.71371598778513689609	10.70689924154012473423
H	8.35092584408192450951	4.12399165683801527393	10.39727483644927374939
C	7.18182250135804878255	4.82032946281918395215	12.06035412269412532282
C	7.96854730942060918153	4.07048050081131140843	13.05947909435453091476
C	8.02992004905634715328	4.47688243716300693364	14.39409175844816424217
H	7.49576722559644625932	5.35846545075190672236	14.71820596156238103447
C	8.78906656994569779329	3.77161896745880964588	15.31038814072180542780
H	8.80828382018536260034	4.09461648495275820636	16.34642715973956583753
C	9.55342971446269828562	2.68847515457587071808	14.91686717138594531207
H	10.18037490971254399597	2.16948060695595090763	15.62983916019167551781
C	9.55381329914567700712	2.28223650114356813035	13.58378960274764324367
C	8.71534893038967695134	2.95075648788819000501	12.69341613550420788670
H	8.66265778207318248860	2.57824223973135335797	11.68209422716273060416
C	10.46878762741627966193	1.22220697409069467554	13.12156050281585528694
C	10.56095380794032934091	0.00144232010717039710	13.78506141836822962432
H	9.90508494538288886133	-0.17584702038832450421	14.63194990727277478015
C	11.45108555232678604341	-1.00068927240586202920	13.41274865443345376548
C	11.45124280262600535707	-2.25207610436600313975	14.34091999551577600869
C	12.26806097129738226670	-0.78450776193889404375	12.29457103489905911431
C	13.34214982129821613910	-1.77241975960288811187	11.74851468764477324669
C	12.16177107754266728534	0.42811999870120526213	11.61493163268845130176
H	12.81537143678470513919	0.60706082188722321202	10.76718551686595759520

C	11.30086919298006797874	1.44576635786413465468	12.01214441210979089192
C	11.33623211610445480346	2.80388801048395785998	11.42135929253090864677
C	11.67588294088727174369	3.85851859648560679972	12.27024352035883936196
H	11.97138556953603760746	3.63859484534521060439	13.28709110769292855991
C	11.60597757860819889686	5.16513077466215797529	11.83292691920395611760
H	11.84528674192151243005	5.97723471525567440210	12.50838735079112495896
C	11.18828897504206132396	5.44857964081533285849	10.54715735906236417918
H	11.05911921566878497458	6.47654183868635424659	10.24533046480416764723
C	10.88787975181667277980	4.41850209409653515991	9.65558373907759204258
C	10.98942551368948628010	3.09874104765067537315	10.10559530145959072911
H	10.76163537930277769306	2.28073311398655986793	9.43432768906340157855
C	10.37260448387111289037	4.77802667165540739802	8.31972196805908303929
C	9.40945700057772427272	3.99402160689731067222	7.68653407986646364236
H	9.07231388308377439955	3.07304815536937336873	8.14160365029969845807
C	8.85116561059160034119	4.40873672668760363536	6.48992110542038425081
H	8.09522675555985316009	3.79409059402825521445	6.01393280010255004697
C	9.22164267466609999246	5.60881792326510986157	5.91362858321230522307
H	8.75309066911030519975	5.94029339410500423213	4.99737963109184235577
C	10.19275314779694951994	6.41323592474857129275	6.51271163005086783926
C	10.76914724027838943243	5.96269643401648163916	7.69854205794999479906
H	11.56001912016227173297	6.54693516056097468692	8.14259949301268193267
C	10.59342682024538007113	7.68010952988428918786	5.87289926343500479078
C	10.85296869840222555581	7.70548439195517520517	4.49916275558810774271
H	10.75370169000733788778	6.77923400647315865797	3.94258893194227910683
C	11.28205233409151908575	8.83593045588679792957	3.81483847875938408833
C	11.58148178042396914122	8.62693881264773665407	2.30007808114544864964
C	11.43752680985078917786	10.02799100876999638388	4.53579505841621877238
C	11.16261637141492535363	10.01135631264354231007	5.89903098649983448354
H	11.28519400096770297637	10.93159280553234147249	6.46206481500079465974
C	10.74818445668297073325	8.87220241549491639432	6.58746275269469894198
C	10.59284066652706179923	8.98107538724827847432	8.06007863075608810277
C	11.77123279437430092287	8.98552012124926946512	8.80664618606238214227
H	12.71558655440668772485	8.87287496797603303378	8.29043197253727370821
C	11.73622102795357058369	9.11369495867790568866	10.18076103973789869883
H	12.65416885587533180058	9.07178655799570421436	10.75620948725633319043
C	10.52768500307412224970	9.30665105080929144776	10.82191306608251935018
H	10.49920575995314742102	9.40569727059756210963	11.89944073183731454435
C	9.33454705068659329470	9.37055443227410833629	10.09958564757581278570
C	9.37863067661768923244	9.16866404024765202507	8.72087326527218031913
H	8.46196136729004244614	9.19610518056378545282	8.14763518036926903676
C	8.12915375103774451304	9.75045978845939487201	10.87403676709500999209
C	8.27199539626592539321	10.84512899627075555031	11.72829346123843308192
H	9.19423117885717289255	11.41320655562186736631	11.72036367197902073656
C	7.24381432011800630022	11.20392537307328595375	12.57767783561301477846
H	7.36740472076265895396	12.04662592709248514211	13.25068247748722072288
C	6.07040878256596805329	10.47917681537721534824	12.58976209988289340913
H	5.29290926894532098856	10.73557721716167279169	13.29450676954384391593
C	5.86478565388874528708	9.40698719825228479863	11.71212242489011856605
C	6.91648165593113439797	9.06662687912743869845	10.85197714202463004085
H	6.81034410700291648055	8.24020971636142007810	10.16793082081633947666
C	4.54850138636976986106	8.74207232649110466127	11.80500858275669173736
C	3.42618742672783849201	9.54882286173217131875	12.03944764884544049721
H	3.54413744392820806439	10.62541962010921281490	11.97260022702465320776
C	2.16405178855954538619	9.06256035336899401500	12.34747659523681129201
C	1.08362930122092171459	10.15653757731214668070	12.60148109429296958695
C	1.99104974655379973747	7.67379796981962947200	12.42209837109561654245
C	0.68205946187846444584	6.93700247601395414421	12.83310320722409159089
C	3.08152970182856389414	6.86300685967041257385	12.11967534283695435704
H	2.94487431808566135771	5.78650132086053758940	12.13471266464243569771
C	4.34786351446118413122	7.35451964733168850330	11.80992257523068644787
O	13.07902229658742321305	11.72557708350101535189	4.29367487402178138467
C	11.92099924196674898269	11.39785655887932058761	3.97218773411576409060

Complex of $[TP[3]COO]^{6-}$ + PhenylalanineH^{+} (Coordinates from ORCA-job E - 3762.460100028929

O	13.51790503606565	-2.43898079674646	12.86193697716356
O	14.39403534135500	-0.81404216473183	14.15111362954030
O	9.92871578033489	-3.02143100153712	12.97907687964924
O	11.11185821593189	-2.56068143322231	14.83954352757954

O	0.83792014195513	10.59436401180924	13.22267015317206
O	1.62571866677583	11.38953537675489	15.17538840762358
O	1.69947854185146	8.52448729374509	16.40921856717330
O	3.51848011648439	9.39325118292378	17.41426036319906
O	13.18831770002821	12.15995741940557	3.57191675459800
O	12.90923071747319	8.87002227333825	2.29451513860514
O	14.84344844953300	9.53860053479027	3.23426466678593
C	6.65592205461733	6.40600496930535	12.98156453996373
H	5.70338902710367	5.98489557536400	12.67357881211072
C	6.66585841855067	7.69341555338789	13.51615103236832
C	7.86554012575330	8.23733194355541	13.98365231187918
H	7.87241723822973	9.23144074596918	14.42334142093156
C	9.04994231099072	7.52684691845597	13.82623376624139
H	9.99208163772635	7.96739460448400	14.14073446979172
C	9.04076727524913	6.25831185503340	13.25948535883184
H	9.98665206691150	5.74210910809433	13.12608607335226
C	7.83513458150093	5.65212247483082	12.87001880303845
C	7.81858048093271	4.25682093082420	12.36852488357026
C	6.73118255814271	3.73209470998478	11.65323530869487
H	5.86299699279944	4.34490006270025	11.43768955252394
C	6.76365307364170	2.42727543626637	11.17554821119585
H	5.91660967714849	2.04394165222678	10.61300901765822
C	7.87795707318911	1.62049815023380	11.38035732454450
H	7.90528407205339	0.61337070596826	10.97327624219384
C	8.96949578847616	2.11461790333639	12.10390931825568
C	8.91192228685371	3.41602132991257	12.60616138154132
H	9.73842858330952	3.76420931555570	13.21472214669988
C	10.14964791940289	1.26386588280381	12.37992735135705
C	9.94004418387404	-0.05779223833146	12.79172185671703
H	8.92002534632664	-0.42343674307719	12.88232728949564
C	10.98704996404153	-0.90057459040131	13.15318029576960
C	10.66076825930349	-2.27752600525693	13.69256492335267
C	12.30180856376969	-0.41610744102617	13.09686729993468
C	13.49389889211160	-1.29654849558512	13.40427532889500
C	12.51971724913108	0.89401187631689	12.67865349639361
H	13.54223559825104	1.25583168214494	12.60173699724968
C	11.47461670861814	1.74597055102680	12.30605866169380
C	11.83605346472501	3.10220028834495	11.81868401560353
C	12.68433565395677	3.91082500180092	12.58157956245243
H	13.03391369014394	3.56610117117637	13.55099652853671
C	13.06182109319317	5.16034292409796	12.10253868492898
H	13.71401831468237	5.79270458806440	12.69825266964554
C	12.62649914818589	5.60488325885331	10.86082528672366
H	12.97155347528656	6.57105428559746	10.50930834139295
C	11.78066091430922	4.81129694698980	10.06750517173493
C	11.38290659884367	3.56822019787680	10.58147068193508
H	10.71449162159605	2.92775157884409	10.01603681774015
C	11.35378975206462	5.25355937571520	8.71203972443013
C	10.73352813159236	4.36151586932326	7.82317951924996
H	10.54656395028725	3.33271451335487	8.11022723932720
C	10.35292563022249	4.77188372672485	6.55178076492886
H	9.85633711001820	4.06910777997399	5.88834067617301
C	10.61645255052598	6.06292626889053	6.11510153177052
H	10.32908691162206	6.36860995202180	5.11308738078961
C	11.27066376276348	6.96634431296901	6.96283401468024
C	11.60058695102659	6.55911616631018	8.25980283483058
H	12.09961948775268	7.27538563605838	8.90285551962868
C	11.73569700893658	8.27153388892758	6.43235980238544
C	12.33687676399723	8.26986412064806	5.16540123095570
H	12.39925954510213	7.33356730302340	4.61658908588832
C	12.91358424642597	9.40644027793812	4.60747563647357
C	13.60729833469723	9.27642877865895	3.26759423683501
C	12.90770693047990	10.60233606843376	5.33902099837480
C	12.30536862772684	10.61477277309226	6.59436342531527
H	12.26496436289415	11.55074365050022	7.14604965430966
C	11.70120849192730	9.48259449361450	7.15144350198477
C	11.00745784598164	9.67248051426546	8.45061817046887
C	11.71263961646542	10.18533412776451	9.54413951104480
H	12.78430872707086	10.34780433208446	9.46493199739244
C	11.04085793769350	10.48626859010195	10.72357455197808
H	11.58949698429169	10.88476999675427	11.57242842603800
C	9.66606972956079	10.31104761080435	10.81544002119401
H	9.15885284725318	10.60612774043473	11.72864472546765
C	8.93547235982004	9.79820495652143	9.73161977355427
C	9.63128357893114	9.45927979086554	8.56264551847069
H	9.09742424587903	9.04739182998506	7.71042346034968

C	7.46349538663063	9.64199405238771	9.81692023578846
C	6.65158652990442	9.65888526771752	8.67404041110672
H	7.08523124954248	9.82008021512835	7.69184250224087
C	5.27757723238859	9.47974670966587	8.79169727752614
H	4.65898982324049	9.48175114345197	7.89851590031679
C	4.68561813793674	9.28950502774554	10.03525480995103
H	3.61352249305821	9.12782899575900	10.10241582676670
C	5.46962613340254	9.29521894956151	11.19841578723660
C	6.84956034470489	9.47370828607243	11.06311887273141
H	7.46207639783091	9.48062812025026	11.95370488883549
C	4.83374513139838	9.17869284578709	12.53561046417206
C	3.61675286504733	9.84204670030482	12.74840189490500
H	3.18323742411914	10.41929268020815	11.93621523703425
C	2.96509044438887	9.84012166116856	13.97807306826488
C	1.70429610388952	10.66332150904097	14.14273203762809
C	3.53950642989613	9.14591141058049	15.05215667845985
C	2.85920370266714	9.02778838482210	16.39916091568583
C	4.74720798505450	8.48053311871716	14.85332126048665
H	5.18725480378536	7.92500485644461	15.67849734143831
C	5.40084116982944	8.47432621665338	13.61957431995973
O	14.14146164583304	12.62121898929060	5.55878172312437
C	13.46535061249312	11.89203010222992	4.77673536610423
C	5.61691633818749	2.43270969394060	7.26718408638089
C	6.01674407151551	2.43879747677774	5.78825556245240
C	5.70150644952579	1.08592994404922	5.11886673419640
N	5.27705028774227	3.53458572186645	5.07417450573106
O	4.78379389830630	1.06429692522947	4.25622642778104
O	6.39509370608678	0.11675761021220	5.52081402254499
C	5.89099243435431	3.74578654146942	7.95709130107565
H	6.19107586217718	1.62927127902831	7.74121123705728
H	4.55375749733682	2.17546454697724	7.35128722550867
H	7.08443788855441	2.65649341384020	5.68182762568760
H	5.58084552403580	3.59969740615209	4.10062665769728
H	5.42606265902010	4.43944405610996	5.53002869236705
H	4.27439184137547	3.32642523261282	5.06529307373765
C	7.20320284194517	4.21306939999166	8.08269928784838
C	7.46795107230129	5.42033543264475	8.71982311766849
C	6.42082393460451	6.17595832553476	9.24424787234210
C	5.11047328480898	5.72710743421105	9.11162829780503
C	4.84664010796458	4.51947982194853	8.46748849527055
H	8.02163292630209	3.61354588238846	7.69242919518885
H	8.49416737620248	5.76500055093257	8.82480955580129
H	6.62784941248444	7.10584144155190	9.76336974699762
H	4.29139124207072	6.31442202130193	9.51842231994356
H	3.82221933731710	4.16766765719643	8.36787437582547

Optimized structure of TP[4]COO^{8-}

Coordinates from ORCA-job E= -4276.986393933271

C	-3.45553873086157	4.97245918750392	-4.51106494778303
C	-4.55195922449465	4.28691501123421	-3.96784466648601
C	-4.32549460076342	3.15135824066321	-3.19091866075795
C	-3.03696325284635	2.68011165855375	-2.93315351843942
C	-1.93615630167854	3.34423972408711	-3.51474037772677
C	-2.16891999443420	4.49092320699834	-4.27911189968482
C	-3.62866947482534	6.17388928084102	-5.41612360339956
C	-5.96845951064767	4.79561259082123	-4.13015669138684
C	-0.55594593415419	2.82457618993223	-3.37228361783376
C	-2.84861239616999	1.52788159356774	-2.01499986647284
C	0.50244455342469	3.66144716172054	-3.00310030674117
C	1.78221991280556	3.13600153021342	-2.84558079852213
C	2.02301245089708	1.78087338495696	-3.04555236112683
C	0.97874636900819	0.92898601665497	-3.43460713248494
C	-0.29644882538150	1.47184028188706	-3.60704440310578
C	-3.39450809893452	0.27993710476387	-2.31970944255673
C	-3.16267362711574	-0.83027225025855	-1.49827884034518
C	-2.40605606848157	-0.65729521161194	-0.33034752901973
C	-1.88998532534349	0.59130255967226	-0.00302071769748
C	-2.09532653310694	1.67892591696588	-0.84485548472708
C	3.42915935606363	-0.08644906270041	6.97535392713113
C	4.52598811724835	-0.06205842671123	6.10136274658708
C	4.30785959037456	-0.20974784772511	4.73284369943613
C	3.02641488544640	-0.37704450093879	4.20528102087175
C	2.14925709638107	-0.26729188057614	6.45490127225966

C	3.60708353681141	-0.02180905668918	8.47779473206268
C	5.93424112180971	0.20472056981163	6.58820013527679
C	2.85007222510841	-0.45618448606907	2.73260282118951
C	-0.53258757535107	0.07179253585982	4.98199857514411
C	-1.80266687125159	-0.19958036821831	4.47986629860439
C	-2.00368652398658	-1.24586259864394	3.58597247796656
C	3.42058618570366	-1.50682252482654	2.01293512426731
C	3.20675448254278	-1.63928874103635	0.63597181153843
C	2.44885470610620	-0.66166024870130	-0.02439393483791
C	1.90608189661070	0.40589197799874	0.68173949224263
C	2.08867161257425	0.50426378596627	2.05733001039588
C	-4.03946250327120	-3.88388407117895	-3.55291906896714
C	-0.30846504487523	-4.32620845295117	0.24568061603723
C	-4.37329111175090	-4.80097343514322	-2.55937594134408
C	-4.33261776738520	-4.41919849248962	-1.21428163084516
C	-3.98554515666761	-3.10367020822182	-0.89669996403909
C	-3.62797650833070	-2.18053973120760	-1.88186398412005
C	-3.65605760748898	-2.58807213048576	-3.22371495166489
C	-0.10756593145425	-3.38447863430666	1.24866786804969
C	-1.10666238195057	-3.13576231250103	2.20020964441810
C	-2.29839983200193	-3.86759014137912	2.12981056299115
C	-2.51883147748150	-4.79011495256295	1.10601721516516
C	-1.51251621173566	-5.01707263235767	0.16040920102134
C	-3.81644316873805	-5.50623182235063	1.00462868967944
C	-4.64762607427787	-5.37683845748474	-0.12820851912135
C	-4.20744082072810	-6.35953058471726	2.03777944658580
C	-5.38429746647180	-7.10378565983602	1.97330412676457
C	-6.19564744907689	-7.00104973161698	0.83374651627759
C	-5.81854582195374	-6.13704888491928	-0.19237757344678
C	-5.77903469385319	-7.94809535012982	3.16641275303599
C	-7.44663611749846	-7.83870524910928	0.66705740694373
C	0.42601099925919	-2.79719672675869	-3.22659274576886
C	4.10810541473520	-5.20735384282364	-0.23379235695236
C	1.65204304643506	-3.29126237867872	-3.65929643348452
C	2.65457519268348	-2.40948200466224	-4.07859758494374
C	2.40870459429213	-1.03602798491762	-4.05949506707743
C	1.19312158388348	-0.52618448570521	-3.58738338196327
C	0.19898796851690	-1.42653069458630	-3.17825177360226
C	3.70846945995775	-4.09385089854960	0.49720871266418
C	3.69001831638117	-2.82577004508085	-0.10211214626578
C	4.07298971062420	-2.71385784453776	-1.44068998766571
C	4.44094158113880	-3.83217251739651	-2.19303968377150
C	4.47078250415979	-5.08545722644203	-1.57250861976281
C	3.97737097816545	-2.93425666873872	-4.50577046383550
C	4.79153087243220	-3.67533784176096	-3.62397226901120
C	4.40711161563364	-2.73494126542639	-5.81875289628864
C	5.59688886388324	-3.28454788879438	-6.29568758114602
C	6.38872473940225	-4.05247826634597	-5.42910763072740
C	5.97626084793996	-4.23433527700132	-4.11106699139702
C	7.72697005930833	-4.62130987445409	-5.85136627245442
C	5.95202224138740	-3.11259714446501	-7.75735454497673
O	-6.17161948342909	6.01645752161552	-3.86882912629038
O	-6.84814845063614	3.95828705036065	-4.48280317586330
O	-2.98161615997140	7.21900152189089	-5.11769599034237
O	-4.37529069277423	6.02923215559392	-6.42625300641960
O	-4.93380569060446	-8.78880457863620	3.58772403234175
O	-6.91260396375747	-7.71795873376772	3.67836949079626
O	-7.33576845422361	-9.08066360666429	0.87848450617072
O	-8.49917480879382	-7.24097750720153	0.29969608543641
O	6.27492617196554	-4.15723026321508	-8.39360076267029
O	8.57606473143845	-3.80664266570732	-6.31427260962403
O	7.91193250018279	-5.85885870521612	-5.66760100321769
O	5.86554458270293	-1.94862173210814	-8.24455737057180
O	6.83881307807624	-0.59118249094168	6.20348099648299
O	4.45642378583243	-0.80971096590967	8.98566554283421
O	2.87033156729767	0.78306634275219	9.11704491033059
O	6.10715383118592	1.22961955833524	7.30855638745608
C	1.92523788457530	-0.43311989070668	5.08485416252898
C	0.55612737443709	-0.71523410559025	4.59246719133136
C	-0.92838805933089	-2.05861921657324	3.19805097555329
C	0.33772847324056	-1.78592762795714	3.72127344023055
H	-5.17267874383585	2.64290802400879	-2.73627893627976
H	-1.32438842074228	4.99747647792923	-4.74070250422036
H	0.31707720651493	4.71508809292937	-2.81124353907325
H	2.59608869690826	3.78580744854489	-2.53612779396903
H	3.01698471989471	1.37806502687541	-2.87089349137966

H	-1.10805327453052	0.82981778870310	-3.93569874792935
H	-3.98092045240687	0.16760337929356	-3.22845123153721
H	-2.18479742251311	-1.51128199271999	0.30404182745242
H	-1.30366918378427	0.70917109105468	0.90511974968378
H	-1.66684215540481	2.64812175898824	-0.60295204843544
H	5.15462186511039	-0.15684451062173	4.05226443801570
H	1.30560252753134	-0.32882010798589	7.13850257392506
H	-0.37936597438290	0.91156510700530	5.65490675755062
H	-2.64068395580930	0.42895132788629	4.76809034679017
H	-2.99167849577208	-1.41539418538834	3.16657552788564
H	4.01141109220918	-2.24988457078213	2.54302894407773
H	2.24810677865127	-0.75960585182913	-1.08798807572893
H	1.31728233744612	1.15344506280315	0.15549330070300
H	1.63873070129316	1.32188518614480	2.61450414105069
H	-4.05146405093997	-4.19173546836020	-4.59475762654204
H	0.47320998762646	-4.50519289348159	-0.48858277575522
H	-4.63721523505095	-5.82150705406330	-2.82414691852241
H	-3.99969205898305	-2.79417523998104	0.14368601380180
H	-3.35250583383170	-1.89963413166720	-4.00765745453039
H	0.81348902423104	-2.80782238469714	1.26494151785307
H	-3.08358776239709	-3.69422701746806	2.86193962340328
H	-1.67960096967663	-5.73039660292404	-0.64249137480495
H	-3.56797886686849	-6.45405072724706	2.91253154785061
H	-6.46556531476285	-6.03709112054994	-1.06068082051185
H	-0.35260074460976	-3.48246152967293	-2.90053877327005
H	4.11086703113219	-6.18622568152457	0.23755735508297
H	1.84045960447719	-4.36170809686677	-3.66747856834972
H	3.19135222550129	-0.35509123688459	-4.38532236727394
H	-0.74026156269965	-1.05239714472418	-2.77984143139950
H	3.38271094433046	-4.21387448200821	1.52692851560310
H	4.09237018063346	-1.73488509137856	-1.90964296451670
H	4.74987370006173	-5.96561506497792	-2.14587035827622
H	3.77404000809046	-2.17130975036378	-6.50022839021678
H	6.61445555645495	-4.79287443344540	-3.43007009097482
H	1.17360945117409	-2.42355047479768	3.45020462744598

Optimized structure of TP[4]COO]$^{8-}$

Coordinates from ORCA-job E= -5346.234598754623

C	-4.78375575345211	3.16247846154250	-7.22833499242918
C	-4.70842198207075	2.10810664764211	-8.15057551454263
C	-4.32165164688709	0.84525212761619	-7.70620091846355
C	-4.02081064709229	0.59122677028112	-6.36605796904797
C	-4.13880373360962	1.64293002890106	-5.43040591676678
C	-4.50805610277955	2.91110075237331	-5.88578423598476
C	-5.09299754235304	4.58125954538800	-7.65832363238304
C	-5.08544191471135	2.29016321626492	-9.60569242284036
C	-3.93524508211572	1.42034005403601	-3.97878417435198
C	-3.52963551342754	-0.75045887321768	-5.97068686600017
C	-3.10049509705195	2.24826891723303	-3.22219396690200
C	-2.90648131119973	1.98836355480316	-1.86733425904173
C	-3.53754242797256	0.91245716833249	-1.25256870153758
C	-4.39630260061107	0.08358439494730	-1.98938892881972
C	-4.58700650411715	0.35890143005578	-3.34594817503322
C	-2.35195088129206	-0.85963280027820	-5.22766671831987
C	-1.87020078698777	-2.09695730568568	-4.79387406842670
C	-2.58865966204356	-3.25257464713821	-5.13430869877993
C	-3.74558855593780	-3.15612076136135	-5.89977432876539
C	-4.21885039351974	-1.91538478941374	-6.32094720890169
C	2.45615629009876	-9.35637465670886	0.46350853748473
C	1.08278288647250	-9.57411701250310	0.28887871354303
C	0.22115088512902	-8.48141680792852	0.30801680619476
C	0.67299230277415	-7.16709481477047	0.47607761130946
C	2.90786233889271	-8.05714488170512	0.67101462255131
C	0.49835889260124	-10.95900294084394	0.11524452297643
C	-0.33217832621594	-6.08356867619314	0.34910337291815
C	2.02879635263967	-4.65520247123105	1.72761611616681
C	2.64163651373516	-3.42798532836000	1.94360369553796
C	3.88928690504908	-3.14566695061552	1.39822076940967
C	-1.54342373430025	-6.17106075017623	1.04016605858503
C	-2.54357393890529	-5.20217373688884	0.88977864760844
C	-2.31227078148560	-4.13169150347509	0.01433675226476
C	-1.10543041977815	-4.03230408212658	-0.66947382888121
C	-0.11703132412387	-4.99435582024137	-0.50358515878573
C	-6.01639624134459	-3.29779287229935	-1.54027117512469

C	-5.60708739724841	-6.65133446853747	2.57181680031399
C	-6.47932890942895	-3.22063246660567	-0.23287531044247
C	-6.23431025472476	-2.07470572079679	0.53562119822644
C	-5.54855541503734	-1.00574763076686	-0.04755191690445
C	-5.09262840179743	-1.06581465860150	-1.37077364907751
C	-5.31959472965190	-2.23615171205684	-2.10743747516120
C	-4.38105945216603	-6.56720909540952	1.92276443973258
C	-3.82709398105181	-5.31514967402467	1.61545991155145
C	-4.53752990299836	-4.16584048726458	1.97325521790971
C	-5.79112044407452	-4.24122670102975	2.58527714402575
C	-6.31629738673673	-5.49943597677861	2.89922690517488
C	-6.55890992479523	-3.00922009456669	2.88082395612639
C	-6.72517282152852	-1.97440089763148	1.93280314493158
C	-7.12693311908357	-2.85579884838036	4.14929432937576
C	-7.84133516824147	-1.71721667496558	4.51263001875013
C	-7.98181479169499	-0.67759572654024	3.58185912388293
C	-7.42825110895180	-0.82641645535051	2.31281799985746
C	-8.50252451075560	-1.67015809496095	5.87362681286347
C	-8.67139996698696	0.62443565820953	3.93090092974391
C	6.10309108693948	-1.28700692418631	-4.22865937900322
C	5.45837347494100	-0.63375212519346	-5.28503329450178
C	4.25588001165414	-1.12664827126503	-5.78045046981045
C	3.69124595902811	-2.27572812685454	-5.24201203499859
C	4.32283396797571	-2.95152241947439	-4.18766455134049
C	5.52313207852000	-2.44104382376887	-3.68506817814070
C	2.37816498497033	-4.42149149743568	-3.50000020243429
C	3.76211240638549	-4.24111759828302	-3.71388796278691
C	1.89082227179861	-5.71521767906764	-3.32316044381606
C	2.72425472592691	-6.83301426329608	-3.25170908662784
C	4.11196391848524	-6.63457258376147	-3.33709087526256
C	4.60090484248528	-5.35332031889425	-3.60274774961334
C	5.14128421933403	-7.72981993772052	-3.13532067140080
C	2.09804099200513	-8.21198797026531	-3.14203256810324
O	-6.19108431522311	2.85753455103844	-9.84255289695138
O	-4.28901945561684	1.83097703305277	-10.47416665398769
O	-6.00620778211165	5.19312955293611	-7.03400657441165
O	-4.38069361535125	5.06099094285900	-8.58695464088816
O	-7.79449356660926	-1.97323873375847	6.87676487398170
O	-9.73118650527075	-1.37036452284813	5.89985229753698
O	-9.56337223812641	1.03984255792955	3.13618495665178
O	-8.27511257840127	1.21969745492624	4.97429566843913
O	2.85766062259526	-9.21376288367789	-3.28115350666176
O	5.34779675670834	-8.09822285675267	-1.94291451814514
O	5.77671825749646	-8.12407645220851	-4.15333445994980
O	0.84654224890770	-8.27067851016906	-2.95763000158400
O	-0.15386267402647	-11.18170680154431	-0.94484195390353
O	0.67182583258932	-11.77542781207464	1.06498582216161
C	2.05492072660740	-6.94638446366430	0.67317043357571
C	2.65443074742163	-5.61245686217856	0.91819360654596
C	4.56817994606003	-4.10561021432406	0.63145134301207
C	3.91280889586719	-5.31938584073477	0.38308174537814
C	10.08140897559934	0.95915241451320	1.51090449749464
C	9.62033223742762	-0.02466546130654	2.39781221053479
C	8.92933800758950	-1.12010675736751	1.88371628508183
C	8.68335888797961	-1.27497790640109	0.51770999161557
C	9.82640680470188	0.81923057815197	0.14949325592791
C	7.96662574385028	-2.49304944540882	0.06443554317759
C	9.88284422524077	0.05117314948971	-2.73938896884627
C	9.63995240696511	0.02721493761375	-4.10918606681129
C	8.40940183950326	-0.39190954254998	-4.60102341274055
C	6.68666961314269	-2.75560376203509	0.55194638106404
C	5.94274926601479	-3.86416017292974	0.12073258713107
C	6.55177966173333	-4.73664153049078	-0.79440804571328
C	7.83903665906691	-4.49712874779546	-1.26273038659054
C	8.54849896363440	-3.37651476317988	-0.84981345014002
C	9.13674420392070	-0.28055852674346	-0.37582554141830
C	8.88412042257979	-0.33376242553326	-1.83558472761489
C	7.39902935391202	-0.79086442815685	-3.71543000680522
C	7.64423227061075	-0.74123878366722	-2.34192891807305
C	1.44307152868386	-3.27881409976457	-3.37967763810349
C	0.25579243636463	-3.23393529015794	-4.11268817176755
C	-0.63494599793172	-2.16093443817355	-3.98411433140620
C	-0.32940842888711	-1.13686801131213	-3.07619809879004
C	0.84319248444969	-1.18503642917183	-2.32998168364305
C	1.73123258232171	-2.24321679024212	-2.48140717230691
C	9.80156120754133	0.09450719552951	3.89611737056758

C	10.90723134286957	2.14234026820922	1.97189436475675
O	10.48255227825773	3.29615591239466	1.67824881220659
O	10.14126868403700	-0.95059503565735	4.52348531977442
O	9.56910811658841	1.22357303776144	4.41718476268648
O	11.98705717968783	1.88057322786471	2.57609665561426
C	3.47199884018599	-10.47501852392088	0.36683554895496
O	3.31722095335873	-11.31149564163177	-0.56927086183165
O	4.41824872247278	-10.47548786766090	1.20718270131353
H	-4.23228210504117	0.03691653775827	-8.42812174893491
H	-4.59860657798598	3.72288586055338	-5.16773878545542
H	-2.58042636660441	3.07463623269876	-3.69964873814265
H	-2.23742117940329	2.61999734189416	-1.28961911736995
H	-3.34860389201232	0.70384138822768	-0.20310663502017
H	-5.26751321933043	-0.25786932476593	-3.92581332560233
H	-1.79581694343488	0.04343602398733	-4.99330479634763
H	-2.25262474249138	-4.22443752980774	-4.78299776726174
H	-4.29878741586822	-4.05638696970010	-6.15266057936116
H	-5.13888401627835	-1.84719798211116	-6.89564257372138
H	-0.83985821329962	-8.64915182639037	0.13799728332374
H	3.96855946893230	-7.90725349739475	0.84905148444095
H	1.07151882285721	-4.87224790993710	2.19061257120053
H	2.14601858372720	-2.68045708871454	2.55672797999612
H	4.32701781741809	-2.17191952285596	1.58777103075346
H	-1.69372501668050	-6.99555980735737	1.73203424466860
H	-3.08566926991823	-3.38657484999045	-0.15564170099423
H	-0.94152877655596	-3.20170628233745	-1.35063386887329
H	0.82025470361436	-4.90806094580581	-1.04295561220975
H	-6.19778004326300	-4.19588830409447	-2.12422849599773
H	-6.02911940283810	-7.62632241579996	2.79907940909555
H	-7.03375222875746	-4.05025223740250	0.19600395025684
H	-5.37560958687789	-0.10638931155863	0.53796126309055
H	-4.94122947329821	-2.32258578966090	-3.12297150385428
H	-3.86571810381059	-7.47735405088337	1.62988684379925
H	-4.11191455556898	-3.18757994015764	1.77082396905434
H	-7.29315793454268	-5.57552510771617	3.36921440512786
H	-6.99768560763268	-3.64936419499399	4.88130552309108
H	-7.56816619657347	-0.03072346275669	1.58500660986135
H	5.89328622843744	0.26761470845338	-5.70842433206497
H	3.75870454439355	-0.61558889590267	-6.60010413108231
H	2.76835806126704	-2.66934553321902	-5.65771524875504
H	6.02980287661933	-2.96171648896019	-2.87528857369526
H	0.82096698958794	-5.86217209265648	-3.21088036341461
H	5.66922448288468	-5.22228927142301	-3.76173530286599
H	4.38814972854088	-6.07279204130249	-0.23515233696699
H	8.57875090204332	-1.88895201244437	2.56787360477022
H	10.16146423020016	1.60241498392027	-0.52614315891532
H	10.85939538976182	0.35126128816084	-2.36960368404825
H	10.42640835653680	0.31789266609378	-4.80000464504635
H	8.23841202706427	-0.43937047560785	-5.67306674713815
H	6.26856507231860	-2.05530346502355	1.26759836137625
H	6.02781867019715	-5.61039882551123	-1.16453164847033
H	8.28595228844746	-5.19120694143273	-1.96948401336451
H	9.54761919667135	-3.18323090688258	-1.23090225603947
H	6.84568952647931	-1.00803978653034	-1.65687845858124
H	0.04818946694263	-4.02422235670435	-4.82993903679078
H	-1.02488784686947	-0.31397204860666	-2.93076364577043
H	1.05896504266245	-0.39687853611277	-1.61389458473661
H	2.64458676484095	-2.28319130103784	-1.89229182257289

8. References

1) René Liedtke, Marcel Harhausen, Roland Fröhlich, Gerald Kehr, and Gerhard Erker, *Org. Lett. 2012, 14(6), 1448–1451.*
2) Koji Yamamoto, Akuto Takagi, Miyako Hada, Ryosuke Taniwaki, Tadashi Mizutani, Yoshifumi Kimura, Yuko Takao, Kazuyuki Moriwaki, Fukashi Matsumoto, Takatoshi Ito, Toshiyuki Iwai, Koichi Hida, Takumi Mizuno, Toshinobu Ohno, *Tetrahedron, 2016, 72(32), 4918-4924*.

3) Dennis Cao, Michael Hong, Anthea K. Blackburn, Zhichang Liu, James M. Holcroft and J. Fraser Stoddart, *Chem. Sci., 2014, 5, 4242–4248.*
